# Node persistence from topological data analysis reveals changes in brain functional connectivity

**DOI:** 10.1016/j.patter.2025.101427

**Published:** 2025-12-03

**Authors:** Madhumita Mondal, Yasharth Yadav, Jürgen Jost, Areejit Samal

**Affiliations:** 1 The Institute of Mathematical Sciences (IMSc), Chennai, Tamil Nadu 600113, India; 2 Homi Bhabha National Institute (HBNI), Mumbai, Maharashtra 400094, India; 3 School of Physical and Mathematical Sciences, Nanyang Technological University, Singapore 637371, Singapore; 4 Max Planck Institute for Mathematics in the Sciences, Leipzig, Saxony 04103, Germany; 5 Max Planck Institute for Human Cognitive and Brain Sciences, Leipzig, Saxony 04103, Germany; 6 Center for Scalable Data Analytics and Artificial Intelligence Dresden/Leipzig, Leipzig, Saxony 04107, Germany; 7 Santa Fe Institute for the Sciences of Complexity, Santa Fe, NM 87501, USA

**Keywords:** topological data analysis, persistent homology, healthy aging, autism spectrum disorder, resting-state fMRI, functional connectivity, non-invasive brain stimulation

## Abstract

Large-scale analyses of brain functional connectivity can uncover disruptions in regional activity and connectivity that are commonly associated with neurological disorders or cognitive decline associated with healthy aging. In our study, we employ persistent homology (PH), a prominent tool in topological data analysis, to investigate changes in resting-state functional connectivity in healthy aging and autism spectrum disorder (ASD). We analyze functional connectivity changes across three distinct scales: (1) global scale (brain-wide changes), (2) mesoscopic scale (resting-state-network-level changes), and (3) local scale (region-level changes). At the local scale, we introduce node persistence, a scalable PH-based measure that detects brain regions with significant differences in healthy aging or ASD. Notably, these regions overlap with regions whose non-invasive stimulation improves motor function in the elderly or alleviates ASD symptoms, suggesting the utility of node persistence in identifying clinically relevant brain regions affected by aging and ASD.

## Highlights

• TDA-based investigation of functional connectivity changes in healthy aging and ASD
• Persistent homology characterizes connectivity at global, mesoscopic, and local scales
• Node persistence, a new scalable measure, detects local functional connectivity changes
• Node persistence highlights clinically relevant regions linked to aging and ASD

## The bigger picture

Understanding how the human brain’s functional connectivity changes with age or in neurological conditions such as autism spectrum disorder (ASD) is crucial for both neuroscience research and potential clinical applications. Large-scale analyses of brain networks can reveal disruptions in regional activity and connectivity patterns that are often associated with cognitive decline, behavioral differences, or neurological disorders. In this study, we provide a comprehensive multiscale view of how brain networks are affected. Importantly, this study introduces a local measure that identifies brain regions with significant differences in connectivity for both healthy aging and ASD. Together, these findings highlight the sensitivity of node persistence to connectivity changes associated with aging and ASD. The broader importance of this research lies in its ability to link mathematical tools with clinically meaningful patterns in brain function. In the long run, the identified regions from the proposed methods may help guide more targeted therapeutic approaches such as non-invasive brain stimulation by highlighting areas most affected by aging or ASD.

## Graphical abstract

**Figure:**
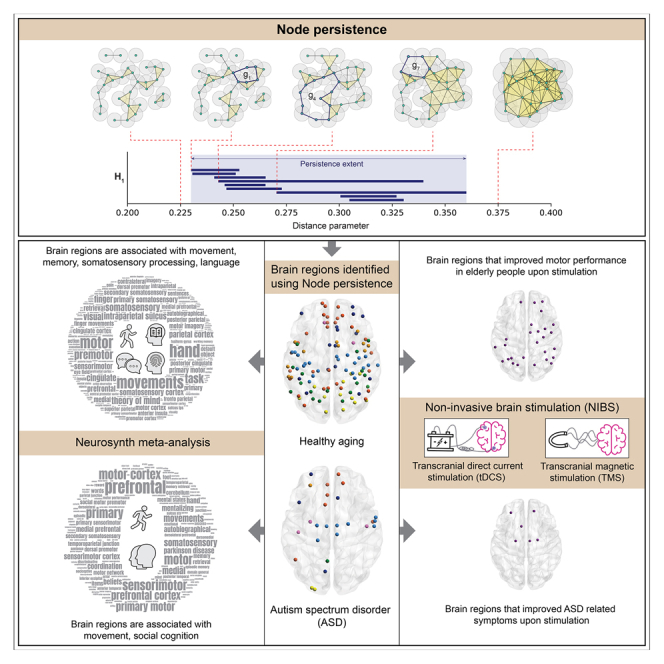

## Teaser

This study employs persistent homology to investigate alterations in brain functional connectivity associated with healthy aging and autism spectrum disorder (ASD). Node persistence, a scalable local measure based on persistent homology introduced in this study, identifies brain regions linked to these conditions, including those with clinical evidence from non-invasive brain stimulation.

## Introduction

Brain functional connectivity analysis is a crucial aspect of neuroscience that investigates how different brain regions interact and communicate. Specifically, it examines temporal correlations between neuronal activity across spatially distinct brain regions, revealing activation patterns that occur during cognitive tasks or at rest.^1^ Such analyses typically employ neuroimaging techniques such as functional magnetic resonance imaging (fMRI), electroencephalography, or magnetoencephalography. Among these, fMRI is particularly prominent due to its ability to capture spontaneous neuronal activity by measuring fluctuations in blood-oxygen-level-dependent (BOLD) signals. Pairwise correlations between BOLD signal time series across different brain regions^2^^,^^3^^,^^4^ can reveal functional connectivity networks underlying the brain. Interestingly, spatially distant brain regions demonstrate synchronized activity even in the absence of external cognitive tasks. This phenomenon is known as resting-state functional connectivity and is estimated from resting-state fMRI (rs-fMRI) data.^5^^,^^6^^,^^7^ Coherent patterns of this activity across anatomically distinct regions are organized into resting-state networks (RSNs), which reflect the intrinsic functional architecture of the brain. These intrinsic functional networks provide a foundation for understanding large-scale brain organization in both health and disease.^5^^,^^7^^,^^8^^,^^9^^,^^10^ In this study, we focus on studying functional connectivity alterations in two distinct biological processes: age-related cognitive decline and atypical neurodevelopment associated with autism spectrum disorder (ASD).

As the global population ages, uncovering the neural correlates of age-related cognitive decline has become increasingly important. Neuroimaging techniques such as fMRI have been instrumental in advancing our understanding of healthy brain aging.^11^^,^^12^ Many studies have identified functional changes in regions such as the prefrontal, medial temporal, and parietal cortices, which are essential for maintaining cognitive performance in elderly individuals.^11^^,^^13^ In parallel, neuroimaging tools have been applied to investigate neurodevelopmental disorders, most notably ASD. A substantial amount of research has investigated the pathophysiology and neurobiology of ASD, providing critical insights into the structural and functional development in ASD relative to typical development.^14^^,^^15^ ASD refers to a broad range of neurodevelopmental conditions,^16^ typically characterized by difficulties in social interaction, verbal or non-verbal communication, and restrictive or repetitive behaviors,^15^^,^^16^ often accompanied by varying levels of cognitive, motor, or memory impairments.^17^^,^^18^^,^^19^ The prevalence of ASD is increasing worldwide^20^^,^^21^ and, while early diagnosis is crucial for effective intervention, it remains equally important to ensure diagnostic reliability and accuracy.^22^ Together, these findings underscore the role of neuroimaging techniques, particularly fMRI, in elucidating the neural mechanisms underlying both healthy aging and ASD.^23^^,^^24^^,^^25^ However, the high complexity and dimensionality of fMRI data have driven the need for advanced analytical tools capable of capturing altered functional connectivity beyond conventional methods. Topological data analysis (TDA) offers a robust multiscale framework for extracting meaningful features from such high-dimensional datasets.^26^

By incorporating principles from algebraic topology and computational geometry, TDA characterizes the inherent shape of high-dimensional datasets.^27^^,^^28^^,^^29^ In this study, we focus on persistent homology (PH), a central tool in TDA that captures topological features or “holes” in the dataset at multiple scales. These features include connected components (*H*_0_), loops (*H*_1_), and voids (*H*_2_), which correspond to zero-, one-, and two-dimensional holes, respectively. These topological features can then be compactly summarized using representations such as persistence barcodes, persistence diagrams, or persistence landscapes, each capturing the evolution of features across multiple scales.^30^^,^^31^^,^^32^^,^^33^ Unlike traditional graph-theoretical or network-based methods, which often rely on ad hoc threshold parameters to define connectivity, PH operates across multiple scales, eliminating the need for arbitrary parameter selection. This multiscale framework enables a more robust and unbiased characterization of topological features in complex datasets.^32^^,^^34^ PH has found widespread application across diverse scientific domains, including biology,^35^^,^^36^^,^^37^^,^^38^ finance,^39^^,^^40^^,^^41^^,^^42^ physics,^43^^,^^44^^,^^45^ and machine learning,^46^^,^^47^^,^^48^ as well as image and signal processing.^49^^,^^50^ In particular, PH has found prominent applications in neuroscience due to its ability to encapsulate the multiscale structure of brain connectivity.^51^^,^^52^^,^^53^^,^^54^^,^^55^^,^^56^^,^^57^^,^^58^^,^^59^^,^^60^^,^^61^^,^^62^^,^^63^ It has been shown to effectively distinguish typical and clinically impaired brain states, providing valuable insights into neurological conditions like ASD,^57^^,^^58^ Parkinson’s disease,^60^ Alzheimer’s disease,^56^^,^^59^ and age-related cognitive impairment.^61^

While PH is well suited for summarizing the global topological structure of complex datasets, it lacks the ability to provide local topological information.^31^^,^^32^ This limitation arises because homology groups are computed on simplicial complexes constructed from the entire dataset, thereby obscuring node-level details. Furthermore, the representatives of homology classes, that is, cycles or holes, are inherently non-unique.^32^^,^^64^ Nevertheless, understanding how specific brain regions influence global topological changes is crucial in functional connectivity analysis, especially in clinical applications where local alterations often contribute to global functional disruptions.^5^^,^^8^^,^^10^ To address this limitation, several studies have investigated the potential for extracting local topological information from PH.^48^^,^^51^^,^^52^^,^^65^^,^^66^^,^^67^ In particular, Lord et al.^52^ showed that in healthy individuals, topologically central nodes in the persistence scaffold, which summarize one-dimensional holes, may support functional integration across brain modules. Additionally, Liang et al.^57^ constructed simplicial complexes at specific thresholds and found significantly fewer one-dimensional holes within certain brain regions in the ASD group compared to the typically developing (TD) controls. However, as data size and dimensionality increase, these methods tend to become increasingly computationally expensive. As a result, local techniques based on PH have seen limited applications in large-scale neuroimaging datasets, highlighting the need for more scalable and efficient methodological advancements.

**Figure 1:**
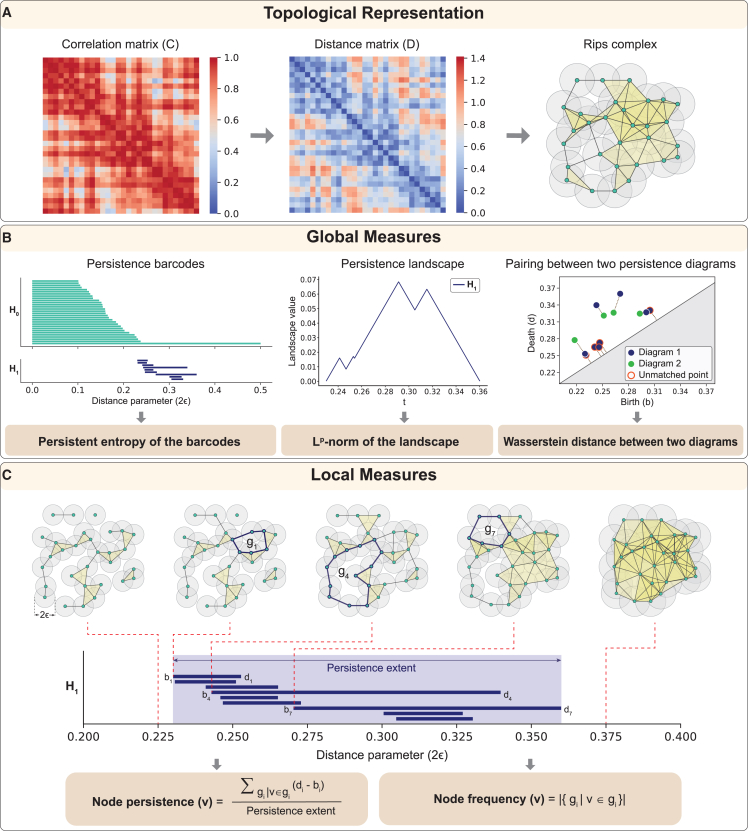
Schematic overview of persistent-homology-based characterization of functional connectivity (A) Topological representation: from the functional connectivity matrix, which is a type of correlation matrix, the distance matrix ($D=\sqrt{2\left(1-C\right)}$) was constructed. Next, this distance matrix was used to build a Rips complex. Note that the Rips complex shown may not reflect the exact spatial embedding of the nodes from the distance matrix. (B) Global measures: we computed persistent entropy, which quantifies the Shannon entropy of the persistence barcodes, and the *L*^1^-norm and *L*^2^-norm, which capture the overall magnitude of the persistence landscape, to investigate brain-wide and resting-state network-level changes in functional connectivity. Next, we compared the 1-Wasserstein, 2-Wasserstein, and bottleneck distances between intra-group and inter-group persistence diagrams. To compute these distances, points in the diagrams are matched pairwise or with the diagonal if no suitable pairing is possible, and the distances are then calculated based on the lengths of the connecting lines. (C) Local measures: we computed node persistence and node frequency to characterize region-level changes in functional connectivity. These measures quantify the influence of a node (*v*) on one-dimensional homological features (*H*_1_). Rips complexes corresponding to five different distance parameters (2*ϵ*) are shown, where *ϵ* denotes the radius of the balls centered at each point, and *g*_*i*_ denotes a representative cycle in *H*_1_ with birth *b*_*i*_ and death *d*_*i*_.

The primary goal of our work is to develop efficiently computable PH-based metrics and utilize them to identify alterations in the brain functional connectivity in healthy aging and ASD and to link them with cognition and behavior. To this end, we utilized functional connectivity (FC) matrices of 225 subjects from the MPI-LEMON dataset^68^^,^^69^ and 820 subjects from the ABIDE-I dataset.^70^^,^^71^ First, we computed PH-based measures at three distinct scales, namely global, mesoscopic, and local, to compare healthy young and healthy elderly individuals in the MPI-LEMON dataset and ASD and TD individuals in the ABIDE-I dataset. At the local or region-level scale, we introduced two PH-based metrics, node persistence and node frequency, which quantify the contribution of a node to one-dimensional homological features. These measures are computationally more efficient than existing local PH-based measures, making them suitable for large-scale brain connectivity analysis. Figure 1 presents an overview of PH-based measures. Second, we utilized Neurosynth meta-analysis^72^^,^^73^ to identify the cognitive domains associated with those brain regions that exhibit significant group differences in our proposed local topological measures. Third, we conducted a correlation analysis to examine the relationship between PH-based measures and phenotypic test scores of individuals in the MPI-LEMON dataset as well as clinical symptom severity scores for individuals with ASD. Fourth, we compared the regions with significant between-group differences in local topological measures against clinically relevant regions documented in existing non-invasive brain stimulation (NIBS) studies. Fifth, we evaluated the effectiveness of our proposed metrics by comparing them with an existing PH-based method for detecting region-level topological changes. Finally, we performed a robustness analysis of the proposed metrics to evaluate their sensitivity to different choices of representative cycles of one-dimensional holes.

## Results

In this study, we applied TDA to investigate changes in resting-state functional connectivity associated with healthy aging and ASD. These changes were characterized using topological measures based on PH across three spatial scales: (1) global or brain-wide changes, (2) mesoscopic or RSN-level changes, and (3) local or region of interest (ROI)-level changes. We acquired FC matrices from the MPI-LEMON dataset for the healthy aging investigation and the ABIDE-I dataset for the ASD investigation. The FC matrices in the MPI-LEMON dataset correspond to 153 healthy young and 72 healthy elderly individuals, while those in the ABIDE-I dataset correspond to 395 individuals with ASD and 425 TD individuals. These FC matrices were derived in earlier studies conducted by some of us^69^^,^^71^ from publicly available resting-state fMRI (rs-fMRI) scans. Each FC matrix is a 200 × 200 square matrix representing pairwise Pearson correlations between the 200 ROIs, as specified by the Schaefer atlas.^74^ Next, we filtered the FC matrices to retain only positive correlations and converted them to ultrametric distance matrices.^75^ Our analysis focused on positive correlations, as they have a primary and central role in the structure and organization of brain functional connectivity networks.^76^ Finally, we constructed a filtration of Vietoris-Rips (Rips) complexes on each distance matrix and computed PH-based measures to extract multiscale topological information from the FC matrices (Figure 1A). A detailed description of the FC matrices, Rips complex construction, and topological measures is provided in methods and supplemental information.

### Brain-wide differences in functional connectivity

We determined brain-wide changes in brain functional connectivity between the groups of individuals using three global topological measures derived from PH, namely persistent entropy,^77^ *L*^1^-norm, and *L*^2^-norm^78^ (Figure 1B). To evaluate statistical differences between the groups, a two-tailed two-sample *t* test was conducted.^79^

**Figure 2:**
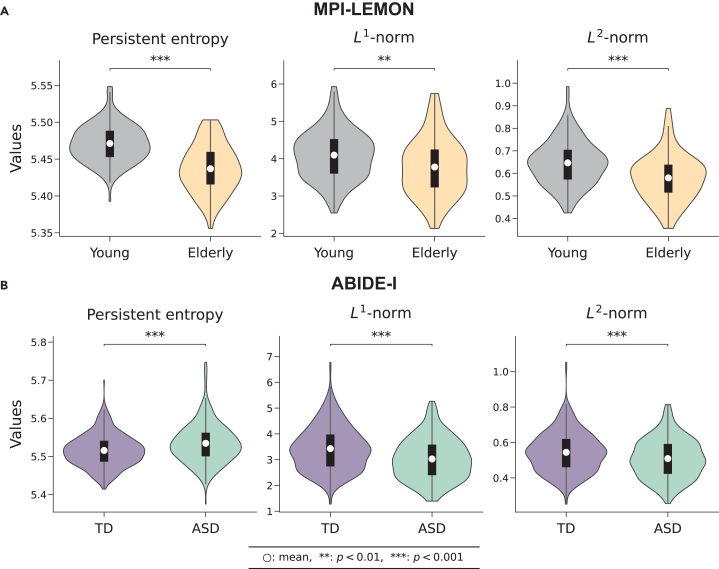
Brain-wide differences between the groups as identified by three global measures: Persistent entropy of the persistence barcodes and ***L***^1^-norm and ***L***^2^-norm of the persistent landscape (A) MPI-LEMON dataset: violin plots corresponding to 153 young and 72 elderly individuals across three global measures. The mean values of all three measures are significantly higher (*p* < 0.01) in the young group compared to the elderly group. (B) ABIDE-I dataset: violin plots corresponding to 425 typically developing (TD) individuals and 395 individuals with autism spectrum disorder (ASD) across three global measures. Average persistent entropy is significantly higher (*p* < 0.001) in the ASD group than in the TD group; however, average *L*^1^-norm and *L*^2^-norm are significantly lower (*p* < 0.001) in the ASD group.

Figure 2 shows violin plots comparing three global measures between the groups for both MPI-LEMON and ABIDE-I datasets. In the MPI-LEMON dataset, the mean persistent entropy of the young group is significantly higher (*p* < 0.001), suggesting that the persistence of its features is more evenly distributed than the elderly group (Table S1). Furthermore, we found that the young group shows a higher mean *L*^1^-norm (*p* < 0.01) and *L*^2^-norm (*p* < 0.001) of the persistent landscape compared to the elderly group, indicating that one-dimensional holes remain more persistent in the young group. In the ABIDE-I dataset, we found that the mean persistent entropy is significantly higher in the ASD group compared to the TD group (*p* < 0.001). In contrast, the mean *L*^1^-norm and *L*^2^-norm are significantly lower in the ASD group compared to the TD group (*p* < 0.001 for both).

Furthermore, to examine the relationships among the three measures, we performed a Spearman correlation analysis. The analysis was conducted separately for each group, namely young and elderly individuals in the MPI-LEMON dataset and TD and ASD individuals in the ABIDE-I dataset, since each measure showed significant differences between the groups across both datasets. The results revealed a strong positive correlation (*ρ* ≥ 0.93) between the *L*^1^-norm and *L*^2^-norm across all groups. In contrast, persistent entropy showed weak positive correlation with either the *L*^1^-norm or *L*^2^-norm. This suggests that the *L*^1^-norm and *L*^2^-norm are largely capturing similar properties, while persistent entropy provides complementary information about the participants. The correlation coefficients, along with the corresponding *p* values, are provided in Table S2.

The group-wise comparisons presented in Figure 2 correspond to Rips complexes constructed from FC matrices with positive correlations only. We also constructed Rips complexes from FC matrices containing the full range of Pearson correlations and found that the group differences in the three global measures remained consistent (*p* < 0.05) with those obtained using only positive correlations in both the MPI-LEMON and ABIDE-I datasets. Figure S1 displays violin plots comparing the three global measures across groups in the MPI-LEMON and ABIDE-I datasets, considering the full range of Pearson correlations from the FC matrices. The findings imply that positive correlations significantly impact overall brain functional connectivity, as the global outcomes using all correlations closely resemble those obtained solely from positive correlations. The result also highlights that positive correlations are sufficient to reflect the overall functional organization of the brain.

Additionally, we compared 1-Wasserstein, 2-Wasserstein, and bottleneck distances between intra-group and inter-group persistence diagrams for both datasets^34^ and conducted a one-tailed two-sample *t* test to evaluate statistical differences. Figures S2 and S3 illustrate the corresponding violin plots based on positive correlations and all correlations, respectively. For all three distance measures, inter-group distances are found to be significantly higher than intra-group distances across both datasets. Table S1 reports group-wise averages and *p* values for persistent entropy, *L*^1^-norm, and *L*^2^-norm, as well as intra-group and inter-group comparisons for Wasserstein and bottleneck distances, based on both positive and all correlations from the FC matrices.

### RSN-level differences in functional connectivity

RSNs are critical for understanding the intrinsic functional organization of the brain without the influence of external tasks.^10^ In this study, we focused on seven well-known RSNs defined according to the Schaefer atlas, specifically visual network (29 ROIs), somatomotor network (35 ROIs), dorsal attention network (26 ROIs), salience/ventral attention network (22 ROIs), limbic network (12 ROIs), control network (30 ROIs), and default network (46 ROIs).^10^^,^^74^ We examined each of the seven RSNs separately for each individual and assessed RSN-level changes in brain functional connectivity between groups by applying persistent entropy, *L*^1^-norm, and *L*^2^-norm. A two-tailed two-sample *t* test was conducted to evaluate statistical differences between the groups, and false discovery rate (FDR) correction was applied separately for each measure.

**Figure 3:**
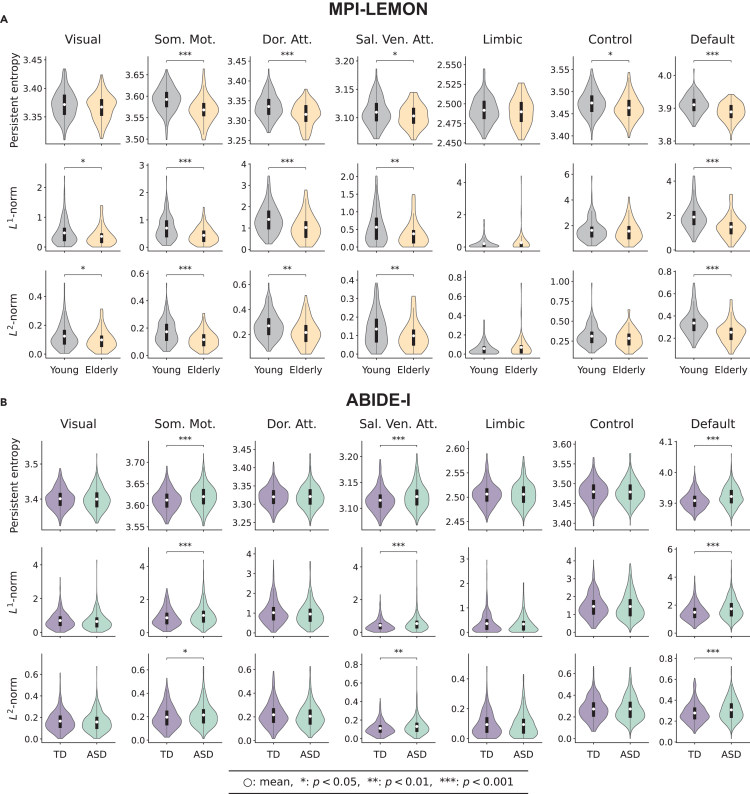
Resting-state-network-level differences between the groups as identified by three global measures: Persistent entropy of the persistence barcodes and ***L***^1^-norm and ***L***^2^-norm of the persistent landscape Each row corresponds to a given measure, and each column corresponds to a given resting-state network (RSN). (A) MPI-LEMON dataset: violin plots corresponding to 153 young and 72 elderly individuals across seven RSNs for each global measure are shown. For the somatomotor (Som. Mot.), dorsal attention (Dor. Att.), salience/ventral attention (Sal. Ven. Att.), and default networks, the mean values of all the three measures are significantly higher (*p* < 0.05, FDR corrected) in the young group compared to the elderly group. For the visual network, only the mean values of the *L*^1^-norm and *L*^2^-norm are significantly higher (*p* < 0.05, FDR corrected) in the young group. For the control network, only the mean of persistent entropy in the young group exhibits a significantly higher (*p* < 0.05, FDR corrected) value than the elderly group. No significant differences are observed between the groups in the limbic network for any of the three measures. (B) ABIDE-I dataset: violin plots corresponding to 425 typically developing (TD) individuals and 395 individuals with autism spectrum disorder (ASD) across seven RSNs for each global measure are shown. For the somatomotor (Som. Mot.), salience/ventral attention (Sal. Ven. Att.), and default networks, the mean values of all three measures are significantly lower (*p* < 0.05, FDR corrected) in the ASD group compared to the TD group. For visual, dorsal attention (Dor. Att.), limbic, and control networks, none of the three measures exhibit significant differences between the TD and ASD groups.

For the MPI-LEMON dataset, violin plots of persistent entropy, *L*^1^-norm, and *L*^2^-norm across RSNs for young and elderly groups are presented in Figure 3A. In the somatomotor, dorsal attention, salience/ventral attention, and default networks, all three measures show significant between-group differences (*p* < 0.05, FDR corrected). Only the *L*^1^-norm and *L*^2^-norm in the visual network, and persistent entropy in the control network, differ significantly. No differences are observed in the limbic network. Figure 3B presents results for ABIDE-I dataset, where significant differences (*p* < 0.05, FDR corrected) across all three measures are found in the somatomotor, salience/ventral attention, and default networks; no differences are observed in the other RSNs. We also performed Spearman correlation analysis between the three measures separately for each RSN. Similar to the global-scale results, the *L*^1^-norm and *L*^2^-norm showed a strong positive correlation (*ρ* ≥ 0.95) across all four groups within each RSN. In contrast, persistent entropy exhibited moderate to high positive correlations with the *L*^1^-norm and *L*^2^-norm. Table S2 provides the detailed correlation coefficients and corresponding *p* values across all the RSNs.

Additionally, we compared 1-Wasserstein, 2-Wasserstein, and bottleneck distances between intra-group and inter-group persistence diagrams for both datasets using one-tailed two-sample *t* tests. Figure S4 presents violin plots comparing intra-group and inter-group distances across both datasets. Table S3 provides group-wise averages; FDR-corrected *p* values for persistent entropy, *L*^1^-norm, and *L*^2^-norm; and intra-group and inter-group comparisons of distance measures across RSNs.

### ROI-level differences in the functional connectivity

In the first subsection, using whole-brain FC matrices, we showed that global topological measures based on PH can effectively differentiate between young and elderly groups in the MPI-LEMON dataset as well as ASD and TD groups in the ABIDE-I dataset. In the second subsection, we extended this analysis to the RSN level by applying the same topological measures to RSN-specific FC matrices. This enabled us to identify the RSNs where group-level differences in functional connectivity are concentrated. In this subsection, we focus on the 200 regions, as defined by the Schaefer atlas, to investigate ROI-level differences in brain functional connectivity. To achieve this, we proposed two topology-based local measures: node persistence and node frequency (Figure 1C and methods). Node persistence quantifies the topological importance of a node by evaluating the duration of its involvement in one-dimensional holes during the filtration process. A higher node persistence value indicates that the node is consistently involved in more persistent one-dimensional holes, suggesting a key role in maintaining the loop structures of the simplicial complex. On the other hand, node frequency measures how many distinct one-dimensional holes a node belongs to, irrespective of their persistence duration throughout the filtration. We computed node persistence and node frequency across the 200 ROIs corresponding to each subject. A two-tailed two-sample *t* test was used to evaluate group-level differences, and FDR correction was applied to adjust for multiple comparisons.

**Figure 4:**
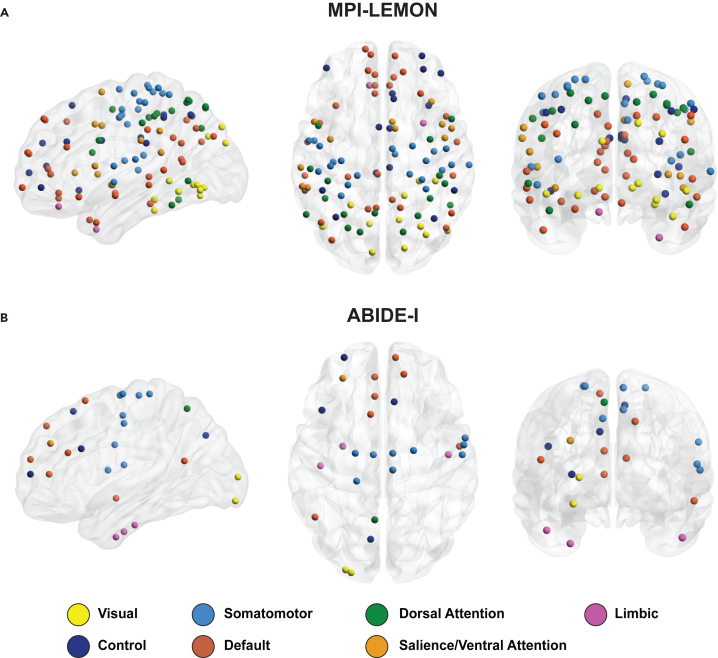
Visual representation of brain regions with significant between-group differences in node persistence (A) MPI-LEMON dataset: 108 regions with significant differences (*p* < 0.05, FDR corrected) in node persistence between the healthy young and healthy elderly groups. For every region, young individuals show higher node persistence compared to the elderly individuals, except for one region in the limbic network (RH_Limbic_TempPole_1). (B) ABIDE-I dataset: 27 regions with significant differences (*p* < 0.05, FDR corrected) in node persistence between the autism spectrum disorder (ASD) and typically developing (TD) groups. All regions reveal higher node persistence for individuals with ASD relative to TD individuals. Each brain region is assigned to one of the seven resting-state networks (RSNs) as defined by the Schaefer atlas. The regions are colored according to their respective RSNs, as shown at the bottom. The visualization was generated using BrainNet Viewer.^80^Table S4 lists the significantly different regions of interest identified via node persistence across both datasets.

Based on node persistence, we identified several ROIs with significant between-group differences in both datasets. In the MPI-LEMON dataset, 108 ROIs exhibit significant differences (*p* < 0.05, FDR corrected) between the young and elderly groups. Among the seven RSNs, the ROIs are categorized as visual (12), somatomotor (22), dorsal attention (17), salience/ventral attention (11), limbic (2), control (15), and default (29) networks. All of these ROIs, except for one in the limbic network (RH_Limbic_TempPole_1), show higher node persistence values in young individuals compared to elderly individuals. In the ABIDE-I dataset, 27 ROIs exhibit significant differences (*p* < 0.05, FDR corrected) between the ASD and TD groups. The ROIs are distributed as visual (2), somatomotor (9), dorsal attention (1), salience/ventral attention (1), limbic (3), control (4), and default (7) networks. All the ROIs show higher node persistence values for individuals with ASD. Figure 4 depicts the ROIs with statistically significant between-group differences (*p* < 0.05, FDR corrected) based on node persistence for both datasets.

In contrast, node frequency reveals 39 and 35 ROIs with significant between-group differences (*p* < 0.05, FDR corrected) for the MPI-LEMON and ABIDE-I datasets, respectively (Figure S5). In the MPI-LEMON dataset, 39 ROIs are distributed as follows: visual (3), somatomotor (6), dorsal attention (9), salience/ventral attention (4), limbic (2), control (3), and default (12) networks. Moreover, the ROIs identified through node frequency are a subset of those identified by node persistence. In the ABIDE-I dataset, 35 ROIs are distributed in six RSNs as follows: visual (5), somatomotor (11), dorsal attention (2), salience/ventral attention (5), control (5), and default (7) networks. Moreover, 21 out of these 35 ROIs overlap with those found via node persistence. Table S4 lists ROIs with significant between-group differences identified by both node persistence and node frequency. For each of the 200 ROIs, group-wise averages of node persistence and node frequency are provided in Table S5, along with FDR-corrected *p* values, for both the MPI-LEMON and ABIDE-I datasets.

### Behavioral and cognitive relevance of ROI-level differences

In the preceding subsection, we found that node persistence can identify 108 ROIs with significant differences between young and elderly individuals in the MPI-LEMON dataset and 27 ROIs with significant differences between ASD and TD groups within the ABIDE-I dataset. These ROIs are distributed across multiple RSNs defined by the Schaefer atlas. Subsequently, to identify the cognitive domains related to these significant ROIs, we performed a Neurosynth meta-analysis (see methods) for each RSN. Only the ROIs exhibiting significant between-group differences in node persistence were considered in this analysis.

In the MPI-LEMON dataset, our analysis primarily focused on somatomotor, dorsal attention, salience/ventral attention, and default networks, as these RSNs not only exhibit significant differences across three global measures (persistent entropy, *L*^1^-norm, and *L*^2^-norm) but also contain the majority of ROIs with significant differences in node persistence. For the ABIDE-I dataset, we focused on the somatomotor and default networks, as they exhibit significant group differences at the RSN level and also contain the majority of ROIs with significant differences identified via node persistence. Although the salience/ventral attention network also showed significant group differences at the RSN level in ABIDE-I, it is excluded here, as only one region is identified using node persistence.

**Figure 5:**
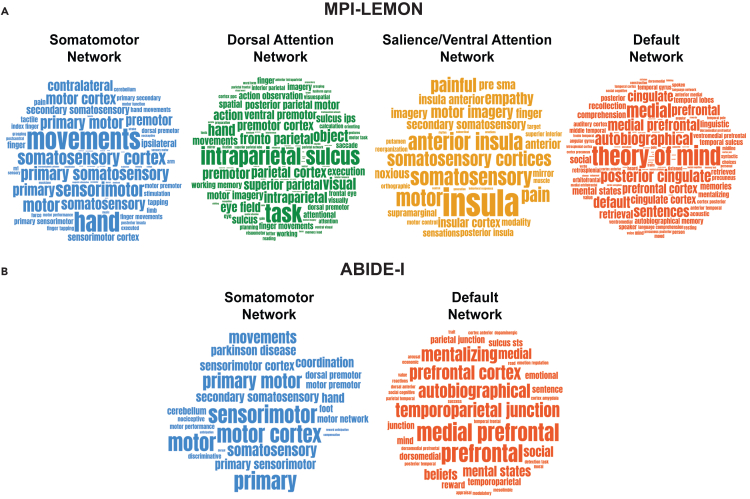
Behavioral and cognitive relevance of ROI-level changes using Neurosynth meta-analysis decoding (A) MPI-LEMON dataset: word clouds highlighting cognitive and behavioral terms related to significantly different age-related brain regions identified via node persistence in four RSNs: somatomotor, dorsal attention, salience/ventral attention, and default networks. (B) ABIDE-I dataset: word clouds highlighting cognitive and behavioral terms related to significantly different brain regions (*p* < 0.05, FDR corrected) between individuals with autism spectrum disorder (ASD) and typically developing (TD) individuals, as identified via node persistence, in two RSNs: somatomotor and default networks. The words are scaled according to their frequency of occurrence. However, the scaling is performed independently for each word cloud. In other words, frequencies are not visually comparable across different word clouds. The word clouds are created using the online tool https://www.wordclouds.com.

Figures 5A and 5B show word clouds illustrating the behavioral relevance of key brain regions in four RSNs for the MPI-LEMON dataset and two RSNs for the ABIDE-I dataset, respectively. We found that ROIs with age-related changes in node persistence are associated with movement in the somatomotor and dorsal attention networks; somatosensory and affective processing in the salience/ventral attention network; and language, social cognition, and memory in the default network. We found that ROIs with ASD-related differences in node persistence are associated with movement in the somatomotor network and social cognition in the default network. The terms linked to significantly different ROIs across all seven RSNs for both datasets are summarized in Table S6.

### Correlation of topological measures with phenotypic and clinical scores

We conducted a correlation analysis to examine the relationship of PH-based metrics with phenotypic test scores for the MPI-LEMON dataset and clinical scores of symptom severity for individuals with ASD in the ABIDE-I dataset. In the MPI-LEMON dataset, significant positive correlations were found between PH-based measures and Trierer Inventar zum Chronischen Stress (TICS) scores at both the brain-wide and RSN levels, particularly within the visual, dorsal attention, and salience/ventral attention networks (Table S7). These correlations suggest that higher PH-based measures are linked to higher levels of chronic stress. At the ROI level, correlations indicated that age-related differences in node persistence within only two brain regions were related to chronic stress (Table S8). In the ABIDE-I dataset, no significant correlations were identified between topology-based measures and clinical scores of ASD symptom severity at the brain-wide, RSN, or ROI levels (Tables S8 and S9). Our findings suggest that PH-based measures capture latent or compensatory mechanisms that reliably distinguish ASD from TD.^81^^,^^82^ However, symptom severity in ASD likely reflects more heterogeneous influences,^83^ including genetic, metabolic,^84^^,^^85^ and developmental factors,^86^^,^^87^ which may not be directly represented in functional connectivity. This could be a reason why PH-based measures fail to show strong correlations with symptom severity. A detailed description of the correlation analysis is provided in Note S1.

### Linking ROI-level differences in topological measures with NIBS outcomes

Alongside the meta-analysis decoding, we conducted an additional analysis to evaluate the relevance of our findings with the existing literature on NIBS in healthy elderly individuals and individuals with ASD. We focused on three commonly used NIBS techniques, namely transcranial direct current stimulation (tDCS), transcranial alternating current stimulation (tACS), and transcranial magnetic stimulation (TMS). For the MPI-LEMON dataset, we investigated the overlap between brain regions exhibiting differences in PH-based measures and those where NIBS has been shown to enhance motor performance in healthy elderly individuals. For the ABIDE-I dataset, we investigated the overlap with brain regions where NIBS has indicated reductions in ASD-related symptoms. The sets of brain regions where NIBS interventions have shown beneficial outcomes were curated in two prior studies conducted by some of us.^69^^,^^71^

**Figure 6:**
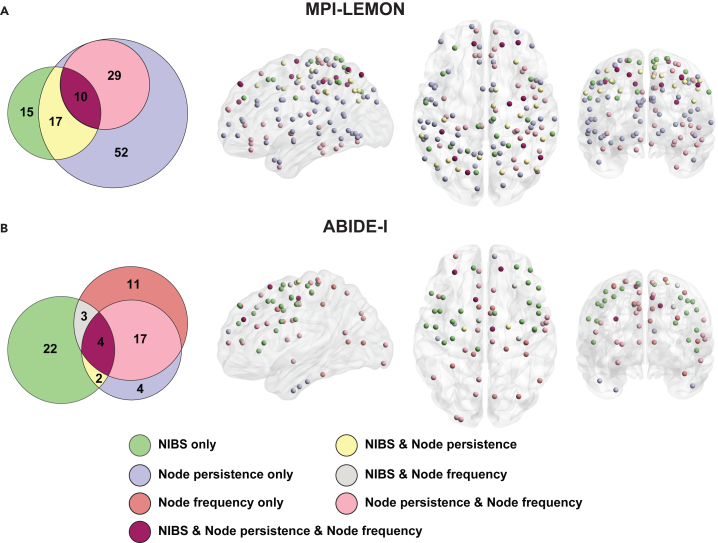
Visual representation of brain regions identified via non-invasive brain stimulation and local persistent-homology-based measures (A) MPI-LEMON dataset: Euler diagram depicting overlaps between brain regions identified by non-invasive brain stimulation (NIBS) (42), node persistence (108), and node frequency (39), along with unique regions in each set. Accompanying this, the brain regions corresponding to each partition of the Euler diagram are visually represented. Within NIBS, 27 of 42 ROIs are identified considering node persistence, node frequency, or both. Seventeen ROIs are uniquely identified using node persistence. Ten ROIs identified via node frequency form a subset of ROIs detected by node persistence. (B) ABIDE-I dataset: Euler diagram depicting overlaps between brain regions identified by NIBS (31), node persistence (27), and node frequency (35), along with unique regions in each set. Accompanying this, the brain regions corresponding to each partition of the Euler diagram are visually represented. Within the 31 ROIs in NIBS, nine ROIs are identified considering either node persistence, node frequency, or both. Two and three ROIs are uniquely identified considering node persistence and node frequency, respectively.

The data derived from previous NIBS studies in healthy elderly individuals revealed four cortical target regions with evidence for improvement in motor function, namely primary motor cortex, dorsolateral prefrontal cortex, posterior parietal cortex, and right supplementary motor area. These target regions are mapped to 42 ROIs in the Schaefer atlas across five RSNs: somatomotor (11), dorsal attention (12), salience/ventral attention (4), control (8), and default (7) networks. The Euler diagram in Figure 6A illustrates the overlaps among 42 ROIs exhibiting improvements in motor function following NIBS, along with the ROIs identified by node persistence and node frequency. Additionally, it highlights both the overlaps and unique ROIs identified across NIBS studies, node persistence, and node frequency. Among these 42 clinically relevant ROIs, 27 are detected using node persistence and ten are detected using node frequency. The clinically relevant ROIs detected by node persistence and node frequency are distributed across five and four RSNs, respectively. No clinically significant ROIs are found within the visual and limbic networks. Further, the ten clinically relevant ROIs detected by node frequency are a subset of those detected by node persistence (Figure 6A and Table S10). In other words, node persistence uniquely identified 17 clinically relevant ROIs.

The data derived from previous NIBS studies in individuals with ASD revealed five cortical target regions that show evidence for improving behavioral or cognitive symptoms associated with ASD, namely premotor cortex, dorsolateral prefrontal cortex, pars triangularis, pars opercularis, and left primary motor cortex. These target regions encompass 31 Schaefer ROIs and are distributed across five RSNs: somatomotor (10), dorsal attention (5), salience/ventral attention (4), control (5), and default (7) networks. The Euler diagram in Figure 6B shows the overlaps among the 31 ROIs exhibiting ASD-related improvements following NIBS and ROIs identified by PH-based local measures. It also illustrates both the overlaps and unique ROIs identified across NIBS studies, node persistence, and node frequency. Among these 31 clinically relevant ROIs, six are detected using node persistence and seven are detected considering node frequency. These clinically relevant ROIs are distributed across three RSNs: somatomotor, salience/ventral attention, and default networks. Both the local measures collectively identified 9 out of 31 clinically relevant ROIs, with two uniquely identified by node persistence and three uniquely detected by node frequency (Figure 6B and Table S10).

### Comparison with existing local measures based on PH

Petri et al.^51^ introduced the concept of homological scaffolds, which are secondary networks that summarize one-dimensional holes (cycles) captured by PH. Building on this idea, nodal persistence scaffold strength (nodal PSS) was proposed as a node-level centrality measure that quantifies the contribution of a region to these cycles.^52^ Specifically, nodal PSS considers the edges involved in representative *H*_1_ cycles extracted from PH via JavaPlex^88^ and assigns strength scores to nodes accordingly. Other local topological measures, such as local PH, can also be defined at the level of individual nodes in a simplicial complex.^66^ In this study, we included nodal PSS as a reference method to compare with our proposed local topological measures of node persistence and node frequency, since all these measures characterize the structure of one-dimensional holes. Further, nodal PSS has also been employed in the context of fMRI data analysis.^52^

Notably, both node persistence and node frequency are less computationally expensive than the construction of homological scaffolds. The latter is significantly more time consuming, particularly during the filtration process, where building the simplicial complex becomes increasingly intensive for larger point sets. This makes node persistence and node frequency better suited for node-level PH analyses on larger simplicial complexes compared to nodal PSS. In particular, when applied to FC matrices with 200 regions, we found that the scalability of homological scaffold construction significantly diminishes. Therefore, to identify ROI-level changes in functional connectivity using nodal PSS, we computed nodal PSS by considering FC matrices at the level of RSNs (RSNs to local). Specifically, the homological scaffolds were constructed independently for each RSN, and nodal PSS was computed for each ROI within these networks. To ensure a consistent comparison with our proposed measures, we also recomputed node persistence and node frequency using FC matrices restricted to individual RSNs. In other words, our comparative analysis focused solely on intra-RSN topological patterns by excluding inter-RSN connections, thereby enabling the detection of ROI-level differences within each RSN. A two-tailed two-sample *t* test was utilized to detect statistical differences between the groups, and FDR correction was applied independently for each RSN.

In the MPI-LEMON dataset, node persistence, node frequency, and nodal PSS identify age-related differences in 59, 40, and 58 ROIs, respectively (*p* < 0.05, FDR corrected). In the ABIDE-I dataset, these three measures reveal significant ASD-related differences in 53, 35, and 45 ROIs, respectively (*p* < 0.05, FDR corrected). The overlaps among these sets and their distribution across RSNs are provided in Note S2. All significant ROIs identified using node persistence, node frequency, and nodal PSS for both datasets are listed in Table S11. Additionally, Table S12 provides the group-wise averages of node persistence, node frequency, and nodal PSS for each of the 200 ROIs across both datasets, along with the corresponding FDR-corrected *p* values.

Finally, we investigated the overlap between our RSN-to-local-level analysis results and clinically validated ROIs from the NIBS literature. The UpSet plots^89^ in Figure S6 illustrate the number of ROIs associated with clinical improvement and how many of these were identified using node persistence, node frequency, and nodal PSS, along with their intersections, for both MPI-LEMON and ABIDE-I datasets. In the MPI-LEMON dataset, of the 42 clinically relevant regions identified through NIBS, node persistence, node frequency, and nodal PSS detected 15, 13, and 18 ROIs, respectively. Similarly, in the ABIDE-I dataset, out of the 31 clinically relevant ROIs identified by NIBS, the three measures identified nine, three, and seven ROIs, respectively. Further details on the overlap between regions identified by local PH measures and NIBS-targeted regions are provided in Note S3 and Table S10.

To summarize, when examining ROI-level differences in functional connectivity during healthy aging and ASD using RSN-level FC matrices, node persistence identifies more brain regions than nodal PSS. In determining clinically relevant regions, nodal PSS detects more regions than node persistence in healthy aging, whereas in autism, node persistence identifies more regions than nodal PSS. However, both node persistence and nodal PSS can uniquely identify certain clinically relevant ROIs across both datasets.

### Robustness of PH-based local measures

The two PH-based local measures proposed in this study are defined with respect to the representative cycle of each one-dimensional hole. Since PH does not guarantee unique representative cycles, the particular choice of cycle may influence the resulting node-level measures. Therefore, it is essential to evaluate the robustness of the proposed measures under different representative cycle selections. In JavaPlex, the representative cycle obtained from a given distance matrix is deterministic for a fixed node order. However, altering the node order can lead to different representative cycles for the same one-dimensional hole. Therefore, to address the robustness of the ROI-based measures, we generated 20 different permutations of node orderings and rearranged the FC matrix accordingly. Next, we computed node persistence and node frequency for all of the subjects from their permuted FC matrices. This procedure provides alternative representations of one-dimensional holes, allowing us to assess the robustness of the proposed measures. Note that this analysis is performed only for the MPI-LEMON dataset.

Since the distributions of node persistence and node frequency were found to be skewed, we adopted robust variability indices: median absolute deviation (MAD) and its normalization with respect to the median (MAD/M).^90^^,^^91^ This index provides a scale-independent measure of dispersion that is less affected by skewness or outliers. Small values of MAD and MAD/M indicate that the measures remain stable across different cycle choices, whereas larger values reflect greater sensitivity of the node-level measures to the specific representative cycles. These indices allowed us to quantify the robustness of the proposed measures under different representative cycle choices. The variability index was calculated separately for each subject and each node. For each node, Table S13 reports the average, first quartile (Q1), median (Q2), and third quartile (Q3) of MAD and MAD/M for the node measures, summarized across individuals in young and elderly groups of the MPI-LEMON dataset.

First, we examined the extent to which node persistence and node frequency values vary across individual FC matrices when different representative cycles are considered. Our results indicate that, for both measures, some sensitivity is present but remains relatively small in magnitude (Note S4). For instance, in the case of node frequency, most nodes exhibited an average MAD value below 1. Given that node frequency can only take integer values, this finding suggests that, although the choice of representative cycles may vary, the number of cycles to which a node belongs remains fairly stable. Overall, variability in node-level measures due to different representative cycle choices was minimal for most nodes in both the young and elderly groups, supporting the robustness of the proposed measures. The summary statistics of both measures across both young and elderly groups are presented in Table S13.

Second, we examined whether the ranking of the nodes based on node persistence and node frequency remains consistent across different cycle choices. To evaluate this, we computed Spearman correlations between all pairs of the 20 node permutations for each subject, separately for both measures. The correlations were then averaged across individuals within each group, yielding four 20 × 20 correlation matrices corresponding to the two measures in the young and elderly groups. We observed that, across all comparisons, the Spearman correlations were consistently high (*ρ* ≥ 0.8) for both measures. This indicates that the node rankings remain largely unchanged across different choices of representative cycles of one-dimensional holes. Figure S7 presents the pairwise correlations across 20 random permutations for node persistence and node frequency in both the young and elderly groups.

Third, we examined whether the group-level differences obtained by node persistence and node frequency depend on the cycle choice. To address this and assess the consistency of our findings, we performed a two-tailed two-sample *t* test to identify significantly different regions or nodes between the young and elderly groups across each of the 20 node permutations. Using node persistence, the number of significantly different regions across permutations ranged from 99 to 116, with an average of 107. In our analysis, we followed the canonical node order provided by Schaefer et al.^74^ and identified 108 regions in total. Of these, on average, 91 regions overlapped with those obtained across different node permutations. Similar results were observed for node frequency (Note S5). Across 20 randomizations, the number of significantly different regions ranged from 38 to 53, with an average of 45. Additionally, we evaluated the extent to which regions identified across different node permutations coincided with NIBS-identified regions. In this case as well, the number of overlapping regions remained within a small range. Table S14 summarizes the results for the PH-based local measures across 20 permutations of node orderings.

Finally, we extended these analyses to the RSN-to-local level, considering node persistence, node frequency, and nodal PSS. The main findings were consistent across all measures. Variability indices showed only minimal fluctuations across nodes, indicating the stability of these metrics at the RSN-to-local level (Note S4 and Table S15). Moreover, both the number of significantly different nodes and their overlap with NIBS-identified regions remained within a narrow range (Note S5 and Table S14). In summary, although the PH-based local measures differ slightly at some nodes depending on the different choices of representative cycles, the overall number of significantly different regions between the groups remains largely within a short interval.

## Discussion

This study attempts to apply PH, a key tool in TDA, to analyze brain functional connectivity in individuals undergoing healthy aging and those with ASD at three different scales: (1) global (brain-wide changes), (2) mesoscopic (RSN-level changes), and (3) local (ROI-level changes). In addition, we introduce a scalable and computationally efficient method for extracting local topological insights from brain functional connectivity using PH.

In this study, we acquired FC matrices derived from the MPI-LEMON and ABIDE-I datasets to investigate changes in brain functional connectivity associated with healthy aging and autism, respectively. The FC matrix, representing pairwise Pearson correlations among 200 brain regions as specified by the Schaefer atlas, served as the initial input for our PH-based analysis. We focused on functionally meaningful interactions by retaining only positive correlations, which were subsequently transformed into ultrametric distance matrices. This transformation allowed us to compute PH-based measures via Rips filtration. In particular, we employed persistent entropy of the persistence barcodes, *L*^1^-norm and *L*^2^-norm of the persistence landscape, for both brain-wide and RSN-level investigations. For the ROI-level analysis, we employed our proposed metrics, node persistence and node frequency, to identify specific ROIs responsible for functional connectivity differences.

At the global scale, our analysis demonstrated significant differences in topological measures between the groups. A higher persistent entropy indicates a more uniform distribution of the persistence of features across the filtration, suggesting the absence of dominant topological structures. Conversely, a lower persistent entropy reflects a concentration of longer-lived features, implying the presence of prominent topological structures within the data.^77^ Higher *L*^1^-norm and *L*^2^-norm of the persistence landscape indicate that one-dimensional topological features persist longer, capturing their dominance and longevity across the filtration. Additionally, 1-Wasserstein, 2-Wasserstein, and bottleneck distances revealed higher inter-group distances compared to intra-group distances for both the MPI-LEMON and ABIDE-I datasets, indicating a greater topological similarity within groups than between groups.

Mesoscale analysis revealed that, although significant topological differences were observed at the global level, they do not arise uniformly across all RSNs. In other words, only specific RSNs contribute to the observed effects. At the local scale, significantly different nodes are mostly concentrated within the RSNs that also exhibited significant group differences during our mesoscale analysis. This highlights the consistency of our findings across different scales. Notably, the healthy aging study indicates a strong agreement between the two measures. In contrast, the ASD study indicates that the two measures may capture distinct aspects of region-level topological differences. Remarkably, our methodology enabled the extraction of multiscale topological features from FC matrices, revealing brain regions responsible for alterations in resting-state functional connectivity associated with healthy aging and ASD.

Next, we used Neurosynth meta-analysis decoding to determine the behavioral and cognitive relevance of the changes arising due to healthy aging and ASD, as identified by node persistence. Our analysis revealed that the brain regions exhibiting age-related differences in node persistence are mainly related to movement, language, social cognition, memory, somatosensory, and affective processing. Previous research suggests that aging impacts various domains of motor performance,^92^^,^^93^ including coordination,^94^ movement variability,^95^ and speed,^96^ as well as language production^97^ and affective processing.^98^ It also leads to diminished somatosensory functions such as sensitivity to warmth, touch, and vibration,^99^ collectively highlighting the physical and emotional changes associated with aging.^98^ Our analysis revealed that the brain regions exhibiting ASD-related differences in node persistence are mainly related to movement and social cognition. Previous meta-analysis decoding suggests that ASD influences the cognitive domains.^17^^,^^18^^,^^19^^,^^100^^,^^101^^,^^102^^,^^103^^,^^104^^,^^105^^,^^106^ Hence, our findings indicate that regions exhibiting differences in node persistence in both healthy aging and ASD are associated with cognitive domains and abilities that are commonly reported to undergo age-related or ASD-related changes.

Following the meta-analysis, we assessed the alignment between our findings and prior tDCS, tACS, and TMS studies. Our analysis revealed that brain regions exhibiting altered PH-based local measures in both healthy elderly individuals and those with ASD overlap with regions previously shown to benefit from non-invasive stimulation, either through improved motor performance in aging or reduction in symptom severity in ASD. Furthermore, in determining clinically significant brain regions, node persistence proved superior in detecting a larger number of clinically relevant brain regions in the context of healthy aging, while for autism, node persistence and node frequency revealed a comparable number of clinically relevant brain regions. Notably, this study also makes an attempt to validate PH-based analyses of brain functional connectivity networks using evidence from non-invasive brain stimulation (tDCS/tACS/TMS) studies.

In aging, postural adaptability typically declines, reflected in reduced center of pressure (COP) complexity due to weakened integration of visual, vestibular, and proprioceptive systems. Prior research^107^^,^^108^ has shown that tDCS over the left prefrontal and primary motor cortices enhances COP complexity and improves balance, indicating that stimulation of these regions can acutely boost adaptability under cognitive load. Similarly, in ASD, tDCS/TMS applied over the left and right prefrontal motor areas and dorsolateral prefrontal cortex has improved Autism Treatment Evaluation Checklist (ATEC) scores, particularly in social relating behaviors and overall functioning.^109^^,^^110^ Consistent with these findings, our node persistence analysis identified regions associated with aging (e.g., 7Networks_LH_Default_PFC_9, 7Networks_LH_SomMot_6, and 7Networks_LH_SomMot_10) and ASD (e.g., 7Networks_LH_SomMot_14 and 7Networks_LH_SalVentAttn_PFCl_1), which are part of the prefrontal and primary motor cortices and overlap with previously reported tDCS or TMS targets. This convergence underscores the potential of node-persistence-identified regions as biomarkers of functional adaptability, while also highlighting the broader utility of PH-based measures in generating hypotheses for clinically relevant stimulation targets, which can subsequently be evaluated through non-invasive stimulation protocols. Although further validation in larger and clinically diverse cohorts is required, these findings suggest a possible bridge between topological analyses and translational neuroscience applications.

We compared our proposed local topological measures with nodal PSS, an existing method that also characterizes one-dimensional holes in functional connectivity networks. Node persistence was found to capture more brain regions with significant group differences compared to nodal PSS in both the MPI-LEMON and ABIDE-I datasets. However, when aligning our findings with non-invasive stimulation outcomes, we found that the regions highlighted by node persistence do not entirely overlap with those identified by nodal PSS. This suggests that different local topological measures may offer complementary insights into clinically relevant brain regions. Due to the higher computational complexity of nodal PSS, these comparisons were conducted using FC matrices restricted to individual RSNs. In contrast, our proposed measures, node persistence and node frequency, are scalable and were applied to FC matrices involving all ROIs in the main analyses reported in this study. This scalability and methodological flexibility make our approach well suited for neuroimaging studies involving large populations.

Finally, as the ROI-based measures are directly related to the representative cycles of one-dimensional holes and such cycles are not unique, we conducted a robustness analysis to evaluate their sensitivity to different cycle choices. Our findings indicate that ROIs exhibit varying degrees of robustness: while some nodes remain highly stable irrespective of cycle selection, some are more sensitive to these choices. Nevertheless, the majority of nodes demonstrate moderate to high robustness. Importantly, although the values for most ROIs differ slightly across different cycles, their relative rankings remain largely preserved. Moreover, when comparing between groups, the majority of ROIs that displayed significant differences remained consistent across different representative cycle choices, highlighting the robustness of the group-level findings.

Although PH-based measures provide rigorous characterizations of higher-order brain network structure, their neurobiological interpretation remains abstract. For instance, a one-dimensional loop may capture functionally related yet indirectly connected regions, although its precise mechanistic relationship to brain physiology remains to be fully elucidated. Thus, PH features are best regarded as descriptive markers of network organization rather than direct mechanistic indicators.^61^^,^^111^ One a priori mechanistic framework that links brain network organization to behavior and cognition is provided by the functional segregation and integration hypothesis.^112^ Functional segregation refers to the specialized processing of information within locally interconnected groups shaped by development and activity-dependent selection. On the other hand, integration refers to coordinated communication across distinct brain regions via a short chain of synaptic pathways. Current evidence indicates that both properties coexist in brain connectivity networks. In other words, information processing in the brain is both localized and distributed, and deviation in either property could lead to atypical behavior or cognitive and motor impairment. PH is fundamentally a global measure, as it tracks topological features such as connected components and cycles across multiple scales. It can therefore be considered to capture the functional integration of brain connectivity. Recent studies have highlighted this perspective,^61^ and future work could explore it more systematically.

In conclusion, we found that PH facilitates the identification of age-related as well as ASD-related changes in functional connectivity at multiple spatial scales. Further, we introduced two local PH-based measures, namely node persistence and node frequency, to detect topology-influenced variations at the level of individual brain regions. Moreover, to support the clinical relevance of our findings for non-invasive brain stimulation, we demonstrated that local measures based on PH can effectively identify brain regions impacted by healthy aging and ASD. One limitation of our approach is that the node-level measures currently consider only one-dimensional topological features such as loops or cycles, potentially overlooking more complex structures. Future research could extend this framework to incorporate higher-dimensional features, such as voids, for a more comprehensive characterization of network topology. Furthermore, node persistence could be utilized to examine local characteristics of brain functional connectivity networks beyond aging and ASD and could potentially be extended to other types of networks, such as task-based fMRI networks. While our analyses focused on assessing group-level differences, validation of the findings, and robustness of PH-based nodal measures, an important next step should be to evaluate their predictive utility. In particular, future work can focus on examining whether node persistence and node frequency can improve out-of-sample prediction of age or clinical status compared with both existing PH-based metrics and standard graph-theoretical measures.

## Methods

In this study, we utilize PH-based metrics to investigate changes in resting-state functional connectivity in individuals undergoing healthy aging and those with ASD. First, we acquired FC matrices obtained from rs-fMRI images of subjects from the MPI-LEMON^68^ and ABIDE-I datasets.^70^ Second, we constructed a sequence of nested Rips complexes^113^ on the distance matrix derived from each FC matrix. Third, we computed topological invariants based on PH to identify the changes in functional connectivity at three different scales: (1) global scale (brain-wide changes), (2) mesoscopic scale (RSN-level changes), and (3) local scale (ROI-level changes).

### FC matrices

The FC matrices used in this study are obtained from preprocessed rs-fMRI images of subjects from the MPI-LEMON and ABIDE-I datasets. These FC matrices were previously generated by some of us using the CONN functional connectivity toolbox^114^ and are available at https://github.com/asamallab/Curvature-FCN-Aging and https://github.com/asamallab/Curvature-FCN-ASD. The FC matrix of each subject is a 200 × 200 square matrix representing the pairwise correlations among the 200 ROIs as outlined in the Schaefer atlas.^74^ Each matrix entry (*i,j*) contains a numerical value representing the Pearson correlation between the time series of ROI *i* and ROI *j*. The time series for each ROI was computed by averaging the time series of all voxels located within that region from the preprocessed fMRI images. For a comprehensive overview of the preprocessing pipeline and FC matrix generation, readers are referred to Yadav et al.^69^^,^^71^ and Elumalai et al.^71^

In addition to assigning each voxel to one of 200 ROIs, the Schaefer atlas^74^ also associates each ROI with one of seven RSNs. These RSNs include the visual network (29 ROIs), somatomotor network (35 ROIs), dorsal attention network (26 ROIs), salience/ventral attention network (22 ROIs), limbic network (12 ROIs), control network (30 ROIs), and default network (46 ROIs). This parcellation method allows for examining brain functional connectivity at the local scale (ROI level) as well as at the mesoscopic scale (RSN level).

A summary of the datasets obtained for this study is as follows.

(1) MPI-LEMON: the FC matrices of 225 subjects from the Max Planck Institute Leipzig Study for Mind-Body-Emotion Interactions (MPI-LEMON) dataset^68^ consists of 153 healthy young (age range 20–35 years) and 72 healthy elderly individuals (age range: 59–77 years).^69^ Notably, the MPI-LEMON dataset does not include any middle-aged individuals.
(2) ABIDE-I: the FC matrices of 820 subjects (age range: 7–64 years) from the ABIDE-I project^70^ consists of 395 individuals with ASD and 425 age-matched TD individuals.^71^

### Construction of Rips complex from FC matrix and PH

Given an FC matrix (***C***), the ultrametric distance matrix^75^ ***D*** is constructed using the distance measure given by

(Equation 1) $$D_{ij}=\sqrt{2\left(1-C_{ij}\right)}\text{,}$$

where ***C***_*ij*_ is the Pearson correlation between the brain regions *i* and *j*. The ultrametric distance in our analyses is defined based solely on positive correlations in the FC matrix, reflecting the dominating role of positive connectivity in supporting overall brain function.^76^^,^^115^ As a result, each value in the distance matrix ranges between 0 and $\sqrt{2}$. These distance matrices are utilized to construct the Rips complex^113^ and to compute PH on the filtration of Rips complexes.

A Rips complex is a type of simplicial complex constructed from a set of points *S* in a metric space. A simplicial complex *K* is a higher-dimensional generalization of a graph, constructed from simplices such as vertices, edges, triangles, tetrahedra, and their higher-dimensional counterparts. For a given radius *ϵ* (>0), the Rips complex is a simplicial complex made up of vertices from *S*, with simplices formed from finite subsets of *S*, where each pair of points in a given simplex has a distance at most 2*ϵ*.^34^^,^^113^ The radius *ϵ* has been chosen as the filtration parameter because it is standard in PH and represents the actual underlying metric distances that directly correspond to correlations between brain regions. Therefore, this choice captures the underlying topological structure of the data. Given a simplicial complex *K*, a filtration of length *n* is a nested sequence of subcomplexes, where each subcomplex is contained within the next. Formally, it is defined as

$$K_{0}\subseteq K_{1}\subseteq K_{2}\subseteq \ldots \subseteq K_{n}=K\text{.}$$

In this sequence, *K*_*i*_ represents a simplicial complex at the *i* th step. Unique values from the distance matrix serve as a filtration parameter of the resulting sequence of Rips complexes. Further details regarding simplicial complexes, Rips complexes, and filtrations can be found in the supplemental information.

Homology is a fundamental concept in algebraic topology that studies the shape of a space by identifying its topological features, often known as “holes” of different dimensions. These features include the zeroth homology group *H*_0_, representing connected components, the first homology group *H*_1_, representing loops or one-dimensional holes, the second homology group *H*_2_, representing voids or two-dimensional holes, and so forth in the simplicial complex.^34^^,^^113^ It provides a way to characterize topological features that remain invariant under continuous transformations like stretching, bending, or squishing. PH extends this idea by analyzing the span of important topological features of a simplicial complex throughout the filtration process.^27^^,^^116^^,^^117^ Given a filtered simplicial complex built from data, PH tracks the birth and death of the features, capturing their persistence. Detailed mathematical expressions of homology and PH are included in the supplemental information.

### Topological measures

#### Persistent entropy

The span of topological features is effectively visualized using persistence barcodes, which distill complex high-dimensional data into a straightforward interpretable format. In the filtration of a finite simplicial complex *K*, the *p*th barcode diagram represents the birth and death of *p* holes during the filtration process.^30^ In particular, for a Rips filtration, a barcode spanning from *b* to *d* on the *x* axis depicts a *p* hole in *K*, with *b* and *d* representing its birth and death filtration values, respectively.

Persistent entropy is a summary statistic of the persistence barcodes that quantifies the spans of the topological features from a sequence of birth-death information.^77^^,^^118^^,^^119^ In particular, it provides the Shannon entropy of the persistence barcodes. Consider a persistence diagram *PD* = {(*b*_*i*_,*d*_*i*_)}, where (*b*_*i*_,*d*_*i*_) represents the birth and death filtration values, with each *d*_*i*_<+*∞*. The persistent entropy associated with *PD* is then defined as

$$PE\left(PD\right)=-\sum_{i=1}^{n}e_{i}\;\log\left(e_{i}\right)\text{,}$$

where

$$e_{i}=\frac{\left(d_{i}-b_{i}\right)}{L}\;\text{and}\;L=\sum_{i=1}^{n}\left(d_{i}-b_{i}\right)\text{,}$$

with *n* denoting the total number of bars and 0 ≤ *PE*(*PD*) ≤ log(*n*). Persistent entropy shows how the spans of the features are spread out. A higher entropy value indicates that the spans of features are more evenly distributed.

#### *L*^*p*^-norm of persistence landscape

Like barcodes, a persistence diagram also visually represents the span of the features. It represents a multiset of points {(*b*_*i*_,*d*_*i*_)} in $R^{2}$, indicating the birth and death pairs for each features.^34^ To perform statistical analysis, a persistence diagram is transformed into a sequence of real-valued functions $\Lambda_{i}:R\to R$ for *i* ≥ 1, known as the persistence landscape,^120^ aggregating essential information contained in persistence diagrams. The *k* th largest value among the set {Λ_*i*_} defines the persistence landscape *λ*_*k*_(*t*). This study considers *k* = 1, as *λ*_1_(*t*) captures the most prominent topological features for each t∈R. As a subset of Banach space, the persistence landscape enables the calculation of *L*^*p*^-norms (1 ≤ *p* ≤ ∞), unlike persistence diagrams. The *L*^*p*^-norms of persistence landscape *λ*_1_(*t*) are defined as^78^

$${\left\|\lambda_{1}\right\|}_{p}={\left(\int_{-\infty }^{\infty }{\left|\lambda_{1}\left(t\right)\right|}^{p}\;dt\right)}^{1/p}\text{.}$$

Moreover, we calculate the 1-Wasserstein, 2-Wasserstein, and bottleneck distances to measure the difference between two persistence diagrams.^34^ Details on persistence landscape construction and Wasserstein distance computation are provided in the supplemental information.

#### Node persistence and node frequency

Although PH captures multiscale topological information, it remains a global measure and limits its ability to characterize individual components’ topology. In brain connectivity analysis, measures such as persistent entropy and landscapes reveal global patterns but cannot localize specific regions responsible for altered connectivity. In this section, we propose two approaches to extract node-level information from the first homology group *H*_1_, namely node persistence and node frequency.

A one-dimensional hole is defined by its one-dimensional boundary, which is a collection of 1-simplices (or edges) that form a cycle. However, this boundary is not unique. Multiple cycles in the simplicial complex might describe the same one-dimensional hole.^31^ Therefore, it is crucial to consider a representative cycle for each class. In our work, we use the representative cycles defined by JavaPlex,^88^ an open-source library designed for computational topology and TDA, which is integrated into our workflow via the Jython interface.

Let $G=\left\{g_{1},g_{2},\ldots ,g_{\beta_{1}}\right\}$ be the set of all one-dimensional holes, where *β*_1_ denotes the number of one-dimensional holes and the *i*th one-dimensional hole is associated with the birth-death pair (*b*_*i*_,*d*_*i*_). The representative cycle of *g*_*i*_ is defined by the set of nodes $\left\{v_{i}^{1},v_{i}^{2},v_{i}^{3},\ldots ,v_{i}^{m}\right\}$. Furthermore, we use the term “persistence extent” to characterize the overall spread or range of persistence barcodes.

The node persistence of a node *v* is defined by aggregating the spans of all cycles that include the node over the persistence extent of the one-dimensional holes. Mathematically, it can be expressed as

$$NP\left(v\right)=\frac{\sum_{g_{i}|v\in g_{i}}\left(d_{i}-b_{i}\right)}{\max\left\{d_{i}\;|1\leq i\leq \beta_{1}\right\}-\min\left\{b_{i}|1\leq i\leq \beta_{1}\right\}}\text{.}$$

The node frequency of a node *v* provides the number of distinct cycles containing the node. Mathematically, it can be expressed as

$$NF\left(v\right)=\left|\left\{g_{i}|v\in g_{i}\right\}\right|\text{.}$$

The two measures, node persistence and node frequency, yield node-level or local insights into PH while taking the one-dimensional holes into account.

### PH-based analysis of brain functional connectivity

We consider FC matrices from two datasets to conduct a PH-based analysis of brain connectivity, namely MPI-LEMON comprising 225 subjects and ABIDE-I comprising 820 subjects. In particular, we employed topological measures derived from PH to compare resting-state functional connectivity between young and elderly groups in the MPI-LEMON dataset and between individuals with ASD and TD controls in the ABIDE-I dataset.

#### Brain-wide analysis

To identify brain-wide or global changes in resting-state functional connectivity, we utilized the 200 × 200 FC matrix for each subject. Each FC matrix was transformed into a distance matrix according to the procedure outlined in the section “construction of Rips complex from FC matrix and PH.” Subsequently, we constructed a filtration of Rips complexes over each distance matrix, indexed by the set of unique pairwise distances serving as the filtration parameters. Our analysis focused on the evolution of topological features as distance varies, captured by PH. We calculated six global topological measures: (1) persistent entropy from the persistence barcodes associated with *H*_0_, *H*_1_, and *H*_2_^77^; (2) *L*^1^-norm of the persistence landscape associated with *H*_1_^120^; (3) *L*^2^-norm of the persistence landscape associated with *H*_1_; (4) the 1-Wasserstein distance; (5) the 2-Wasserstein distance; and (6) the bottleneck distance between the persistence diagrams associated with *H*_1_. We utilized the open-source library Gudhi^121^ to compute these global topological measures.

#### RSN-level analysis

As mentioned previously, the Schaefer atlas^74^ consists of 200 ROIs or nodes partitioned across seven RSNs. To assess RSN-level changes in functional connectivity, we extracted submatrices from the FC matrix containing connections between nodes within each RSN and converted them into distance matrices using Equation 1. Consequently, for each subject, this yielded seven distance matrices, each corresponding to one of the seven RSNs. This results in 225 × 7 = 1,575 and 820 × 7 = 5,740 distance matrices for the MPI-LEMON and ABIDE-I datasets, respectively. The size of each distance matrix varies based on the number of nodes present in the respective RSN. Finally, Rips filtrations for each RSN were constructed using only intra-RSN connections, following the same approach as the brain-wide analysis. The six topological measures listed in brain-wide analysis were also calculated to capture RSN-level functional connectivity changes. These include persistent entropy, *L*^1^-norm, *L*^2^-norm, and 1-Wasserstein, 2-Wasserstein, and bottleneck distances.

#### ROI-level analysis

To identify ROI-level or local changes in resting-state functional connectivity, we utilized the filtration of Rips complexes generated during the brain-wide analysis. Subsequently, we calculated three topological measures based on PH: (1) node persistence, (2) node frequency, and (3) nodal PSS. These measures characterize node-level topological information by extracting the representative cycles of the first homology group *H*_1_, which corresponds to one-dimensional holes or cycles within the Rips complex. Node persistence and node frequency were computed via JavaPlex,^88^ while nodal PSS was computed using the Python package Holes.^51^^,^^122^

### Neurosynth meta-analysis decoding

Using the ROI-level analysis described in the preceding section, we identified specific brain regions associated with altered resting-state functional connectivity in healthy aging (based on the MPI-LEMON dataset) as well as during ASD (based on the ABIDE-I dataset). The cognitive and behavioral implications of these findings were then examined through Neurosynth meta-analysis decoding.^72^^,^^73^ First, we used the Neurosynth meta-analysis tool to identify the terms that are related to behavior, cognition, and perception based on the centroid coordinates of the 200 ROIs in the Schaefer atlas. Second, we focused on nodes exhibiting significant inter-group differences in node persistence values. Each ROI or node belongs to one of the seven RSNs, as specified by the Schaefer atlas. Third, we computed the frequency of terms associated with significant nodes within each RSN. The statistical significance of these frequency counts was assessed using a procedure described in the following subsection.

### Statistical analyses

We performed two-tailed two-sample *t* tests^79^ to examine the statistical significance of the differences in the means of the measured values between (1) young and elderly groups in the MPI-LEMON dataset and (2) ASD and TD groups in the ABIDE-I dataset. To evaluate group-level differences at global and mesoscopic scales, comparisons were performed across three measures: persistent entropy, *L*^1^-norm, and *L*^2^-norm. For 1-Wasserstein, 2-Wasserstein, and bottleneck distances, one-tailed two-sample *t* tests were used to test whether intra-group distances were smaller than inter-group distances. Furthermore, to evaluate group-level differences at the local scale, two-tailed two-sample *t* tests were performed across all 200 nodes for node persistence, node frequency, and nodal PSS.

To assess the statistical significance of the frequency of terms in the Neurosynth meta-analysis decoding, we compared the occurrences of the same terms within an equal number of randomly chosen nodes (proxy ROIs). For instance, considering node persistence, we obtained 108 nodes that exhibit significant differences between the young and elderly groups in the MPI-LEMON dataset. Therefore, from the 200 nodes in the Schaefer atlas, we randomly considered 108 nodes. This allowed us to establish a null distribution for term occurrences by generating 1,000 sets of these randomly selected nodes. We then determined the *Z* score for each term’s frequency in the original set of ROIs. Thereafter, we converted these *Z* scores into *p* values, assuming a normal distribution.^69^^,^^71^^,^^73^

To account for multiple comparisons and reduce the likelihood of false positives, we implemented an FDR correction^123^ to adjust the *p* values. We conducted all statistical tests using the SciPy^124^ and statsmodels^125^ packages in Python. Moreover, the analyses were performed without incorporating any covariates such as gender or age.

### Estimating overlap between brain regions identified by ROI-level analysis and non-invasive stimulation studies

We systematically compared ROIs showing significant between-group differences in local topological measures with those identified in NIBS studies, focusing on three NIBS modalities: tDCS, tACS, and TMS. To compare with brain regions exhibiting functional connectivity changes, we focused on NIBS target regions that improved motor performance in healthy elderly individuals for age-related changes and alleviated ASD symptoms for ASD-related changes. We evaluated the overlap of these target regions with those identified by node persistence, node frequency, and nodal PSS. These overlaps allowed us to assess whether local topological measures of resting-state functional connectivity could identify brain regions whose non-invasive stimulation yields functional benefits in healthy aging and ASD.

The target regions of NIBS experiments were acquired based on a systematic literature review carried out in earlier studies by some of us.^69^^,^^71^ These target regions, which originally correspond to regions in the Brodmann atlas,^126^ were mapped to regions in the Schaefer atlas. This mapping is available at https://github.com/asamallab/Curvature-FCN-ASD.

## Resource availability

### Lead contact

Requests for further information and resources should be directed to and will be fulfilled by the lead contact, Areejit Samal (asamal@imsc.res.in).

### Materials availability

This study did not generate new materials.

### Data and code availability

• The functional connectivity matrices considered in our study were downloaded directly from two GitHub repositories and are publicly available at https://github.com/asamallab/Curvature-FCN-Aging^69^ and https://github.com/asamallab/Curvature-FCN-ASD.^71^
• The code for computing node persistence and node frequency, as well as the code for the analysis and to reproduce the results in this paper, is publicly accessible through the GitHub repository (https://github.com/asamallab/NodePersistence_PH_FCM) and has been archived at Zenodo.^127^
• Any additional information required to reanalyze the data reported in this paper is available from the lead contact upon request.

## Acknowledgments

The authors thank Kelin Xia for insightful discussions. A.S. acknowledges funding from the 10.13039/501100001502Department of Atomic Energy, Government of India (via the Apex project to The 10.13039/100013441Institute of Mathematical Sciences [IMSc], Chennai) and funding from the 10.13039/501100004189Max Planck Society, Germany (through the award of a Max Planck Partner Group).

## Author contributions

Conceptualization, M.M., Y.Y., J.J., and A.S.; methodology, M.M., Y.Y., J.J., and A.S.; investigation, M.M., Y.Y., J.J., and A.S.; visualization, M.M.; writing – original draft, M.M., Y.Y., J.J., and A.S.; writing – review & editing, M.M., Y.Y., J.J., and A.S.; funding acquisition, A.S.; resources, M.M., Y.Y., J.J., and A.S.; supervision, J.J. and A.S.

## Declaration of interests

The authors declare no competing interests.

## Supplemental information

Document S1. Figures S1–S7, Notes S1–S5, and supplemental methods

Table S1. The values of the group-wise average of the topology-based global measures Topology-based measures: persistent entropy, *L*^1^-norm, *L*^2^-norm of the FC matrices for the two groups (young versus elderly) in the MPI-LEMON dataset, and two groups (TD versus ASD) in the ABIDE-I dataset are presented for both considering (1) only the positive Pearson correlations and (2) all the Pearson correlations from FC matrices. Additionally, 1-Wasserstein (W1), 2-Wasserstein (W2), and bottleneck distances of the persistent diagrams for the intra-group and inter-group across both MPI-LEMON and ABIDE-I datasets are presented for considering both (1) only the positive Pearson correlations and (2) all the Pearson correlations from FC matrices. We evaluated the differences between the groups by using a one-tailed two-sample *t* test. This table is organized into three columns for each of the two datasets, with the first two columns containing the group-wise average and their standard error value (up to 3 decimal places). The third column denotes the *p* values, and the color of each cell in this column signifies the presence of significant differences between the two groups (*p* < 0.05). Column B contains the topology-based measures. Visualization of these data is provided in Figures 2 and S1–S3.

Table S2. Correlation between the persistent-homology-based global measures Spearman correlation between persistent entropy and *L*^1^-norm, persistent entropy and *L*^1^-norm, and *L*^1^-norm and *L*^2^-norm are provided along with the corresponding *p* values for both the MPI-LEMON and ABIDE-I datasets. The color of each cell in the Spearman correlation column signifies that the correlation is greater than 0.9. The correlations are performed for both global and resting-state network labels and separately for each group in the MPI-LEMON and ABIDE-I datasets.

Table S3. The values of the group-wise average of the topology-based measures at the resting-state network level Topology-based measures: persistent entropy, *L*^1^-norm, and *L*^1^-norm of the FC matrices for the two groups (young versus elderly) in the MPI-LEMON dataset and two groups (TD versus ASD) in the ABIDE-I dataset are presented, considering only the positive Pearson correlations of the FC matrices. Additionally, 1-Wasserstein (W1), 2-Wasserstein (W2), and bottleneck distances of the persistent diagrams for intra-group and inter-group across both MPI-LEMON and ABIDE-I datasets are presented, considering only the positive Pearson correlations of the FC matrices. We evaluated the differences between the intra-group and inter-group by using a one-tailed two-sample *t* test and corrected for multiple comparisons using FDR with alpha set to 0.05 corresponding to each RSN. This table is organized into three columns for each of the two datasets, with the first two columns containing the group-wise average and its standard error value (up to 3 decimal places). The third column denotes the FDR-corrected *p* values, and the color of each cell in this column signifies the presence of significant differences between the two groups (FDR-corrected *p* < 0.05). Column A contains the topology-based measures, and column B contains the seven RSNs. Visualization of these data is provided in Figures 3 and S4.

Table S4. Brain regions that show significant between-group differences based on the topology-based local measures node persistence and node frequency, considering 200 × 200 FC matrices for the PH analysis To evaluate the between-group differences in each of the 200 brain regions, their group-wise average was compared using a two-tailed two-sample *t* test and corrected for multiple comparisons using FDR with alpha set to 0.05. Note that these regions of interest in the brain were defined by the Schaefer atlas, which parcels the brain into 200 regions that belong to one of the seven RSNs. All brain regions have a common prefix “7networks_,” which has been removed for convenience. Visualization of these data is provided in Figures 4 and S5.

Table S5. Brain regions with values of the group-wise average based on the topology-based local measures node persistence and node frequency, considering 200 × 200 FC matrices for the PH analysis To evaluate the between-group differences in each of the 200 brain regions, their group-wise average was compared using a two-tailed two-sample *t* test and corrected for multiple comparisons using FDR with alpha set to 0.05. This table is organized into four columns for each measure and each dataset, with the first two columns containing the group-wise average and its standard error value (up to 3 decimal places). The third column denotes the *p* values. The fourth column represents the FDR-corrected *p* values, and the color of each cell in this column signifies the presence of significant differences between the two groups (FDR-corrected *p* < 0.05). Column A contains the node IDs, and column B contains the region names provided in the Schaefer atlas.

Table S6. The terms associated with behavior, cognition, and perception, obtained from the Neurosynth tool The terms are obtained for the set of brain regions belonging to the seven RSNs with significant between-group differences in node persistence (global to local) for both MPI-LEMON and ABIDE-I datasets. The frequency of occurrence of the terms associated with an RSN was tested for statistical significance by comparing with the frequencies obtained from random surrogate brain regions and further subjected to FDR correction with alpha equal to 0.05 (see methods). The terms that survived the FDR correction are presented in this table along with their frequencies. The terms and their frequencies in this table are visualized as word clouds in Figure 5.

Table S7. Correlation of persistent-homology-based measures persistent entropy, *L*^1^-norm, and *L*^2^-norm with cognitive and behavioral scores for the MPI-LEMON dataset Correlation of persistent entropy, *L*^1^-norm, and *L*^2^-norm with 52 subtest scores from eight cognitive and behavioral test scores, corresponding to global and RSN level. Spearman correlations and FDR-corrected *p* values are provided for each measure and each test score for both global and RSN level. The FDR correction is performed with alpha = 0.05 corresponding to each RSN. Columns A and B contain the eight tests and 52 subtests, respectively.

Table S8. Correlation of persistent-homology-based local measure node persistence, with cognitive and behavioral scores for the MPI-LEMON and ABIDE-I dataset Spearman correlation of node persistence of those nodes that have significant between-group differences with subtest scores from eight cognitive and behavioral test scores, corresponding to global and RSN level for MPI-LEMON dataset, and with two ASD-related clinical scores corresponding to ABIDE-I dataset. The FDR correction is performed with alpha = 0.05 corresponding to each RSN. Columns A, B, C, D, E, and F contain the RSNs (number of significantly different node persistence regions across groups), tests, number of subtests, total number of correlations, node IDs that have p_value < 0.05, and node IDs with FDR-corrected p_value < 0.05, respectively. Total number of correlations is basically the number of regions with significant between-group differences, using node persistence times total number of subtests that one particular test has.

Table S9. Correlation of persistent-homology-based measures persistent entropy, *L*^1^-norm, and *L*^2^-norm with ASD-related clinical scores for the ABIDE-I dataset Correlation of persistent entropy, *L*^1^-norm, and *L*^2^-norm with two ASD-related clinical scores, corresponding to global and RSN level. Spearman correlations and *p* values are provided for each measure and each score for both global and RSN level. Column B contains the names of the clinical scores.

Table S10. Mapping between the 200 brain regions defined by the Schaefer atlas and the corresponding Brodmann areas, along with results from NIBS and local PH-based measures A Schaefer ROI was mapped to a Brodmann area if the MNI centroid coordinate of the Schaefer ROI lay within the Brodmann area. The mapping and the identification of the brain regions whose stimulation with NIBS methods (TMS, tDCS, tACS) of individuals resulted in positive behavioral and cognitive outcomes that were performed in two previous studies (see methods). The set of Schaefer ROIs that were mapped to these target regions is indicated, along with the topology-based local measures node persistence, node frequency, and nodal PSS that identify the Schaefer ROIs to show significant between-group differences. Visualization of these data is provided in Figures 6 and S6.

Table S11. Brain regions that show significant between-group differences based on the topology-based local measures node persistence, node frequency, and nodal PSS, with RSNs to local To evaluate the between-group differences in each of the 200 brain regions, their group-wise average was compared using a two-tailed two-sample *t* test. The *p* values are corrected for multiple comparisons using FDR with alpha set to 0.05 corresponding to each RSN. Note that these regions of interest in the brain were defined by the Schaefer atlas, which parcels the brain into 200 regions that belong to one of the seven RSNs. All brain regions have a common prefix “7networks_,” which has been removed for convenience. A cell contains the value “No ROIs” if a measure has not identified a brain region belonging to the RSN.

Table S12. Brain regions with values of the group-wise average based on the topology-based local measures node persistence, node frequency, and nodal PSS, with RSNs to local To evaluate the between-group differences in each of the 200 brain regions, their group-wise average was compared using a two-tailed two-sample *t* test. The *p* values are corrected for multiple comparisons using FDR with alpha set to 0.05 corresponding to each RSN. This table is organized into four columns for each measure and each dataset, with the first two columns containing the group-wise average and its standard error value (up to 3 decimal places). The third column denotes the *p* values. The fourth column denotes the FDR-corrected *p* values, and the color of each cell in this column signifies the presence of significant differences between the two groups (FDR-corrected *p* < 0.05). Columns A, B, and C contain the RSNs, node IDs, and region names provided in the Schaefer atlas, respectively.

Table S13. Variability of topology-based local measures node persistence and node frequency across brain regions, quantified using median absolute deviation and median absolute deviation normalized by the median across 20 permutations of node ordering These values are reported for the young and elderly groups in the MPI-LEMON dataset, based on the topology-based local measures node persistence and node frequency, using 200 × 200 FC matrices for the PH analysis. This table is organized into four columns for each variability index with its average, first quartile (Q1), median (Q2), and third quartile (Q3). Column A contains the node IDs of the brain regions provided in the Schaefer atlas.

Table S14. Number of significantly different regions in the MPI-LEMON dataset across 20 permutations of node ordering, compared to the canonical node ordering in the Schaefer atlas The number of regions and the subset that coincide with NIBS-identified regions are presented separately for 200 × 200 FC-matrix-to-local and RSN-to-local analyses. For the 200 × 200 FC-matrix-to-local analysis, values are provided for node persistence and node frequency. For RSN-to-local analysis, values are given for node persistence, node frequency, and nodal PSS. Numbers in parentheses indicate how many regions from a given node permutation are also identified using the canonical node ordering in the Schaefer atlas.

Table S15. Variability of topology-based local measures node persistence, node frequency, and nodal PSS across brain regions with RSN to local, quantified using median absolute deviation and median absolute deviation normalized by the median across 20 permutations of node ordering These values are reported for the young and elderly groups in the MPI-LEMON dataset, based on the three topology-based local measures node persistence, node frequency, and nodal PSS from RSN-to-local-level analysis. This table is organized into three columns for each variability index with its average, median (Q2), and third quartile (Q3). Column A indicates the RSNs, and column B contains node IDs of the brain regions provided in the Schaefer atlas.

Document S2. Article plus supplemental information
