# Supplementary material for: Node persistence from topological data analysis reveals changes in brain functional connectivity
Source: Patterns (N Y). 2025 Dec 3;7(3):101427. doi: 10.1016/j.patter.2025.101427 (PMC13100682; doi:10.1016/j.patter.2025.101427)
Supplement: Document S1. Figures S1–S7, Notes S1–S5, and supplemental methods [file mmc1.pdf]

Patterns, Volume *7*

Supplemental information

Node persistence from topological data analysis reveals changes in brain functional connectivity

Madhumita Mondal, Yasharth Yadav, Jürgen Jost, and Areejit Samal

Supplemental figures

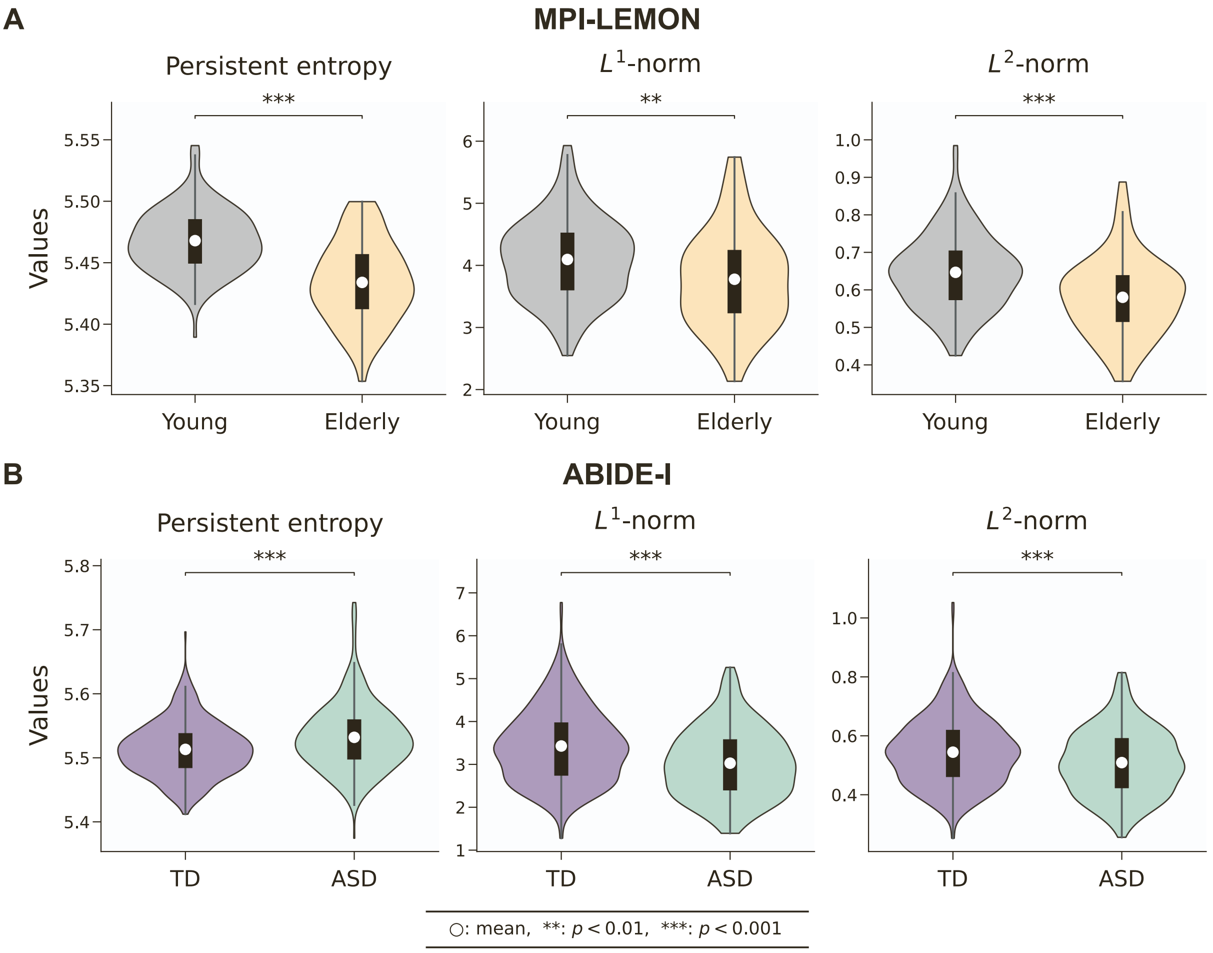

Figure S1: **Brain-wide differences between the groups as identified by three global measures: persistent entropy of the persistence barcodes, L^1-norm, and L^2-norm of the persistent landscape, considering all the correlations from functional connectivity matrices.** (A) MPI-LEMON dataset: violin plots corresponding to 153 young and 72 elderly individuals across three global measures. The mean values of all three measures are significantly higher ($p < 0.01$) in the young group compared to the elderly group. (B) ABIDE-I dataset: violin plots corresponding to 425 typically developing (TD) individuals and 395 individuals with autism spectrum disorder (ASD) across three global measures. Average persistent entropy is significantly higher ($p < 0.001$) in the ASD group than the TD group; however, average L^1-norm and L^2-norm are significantly lower ($p < 0.001$) in the ASD group.

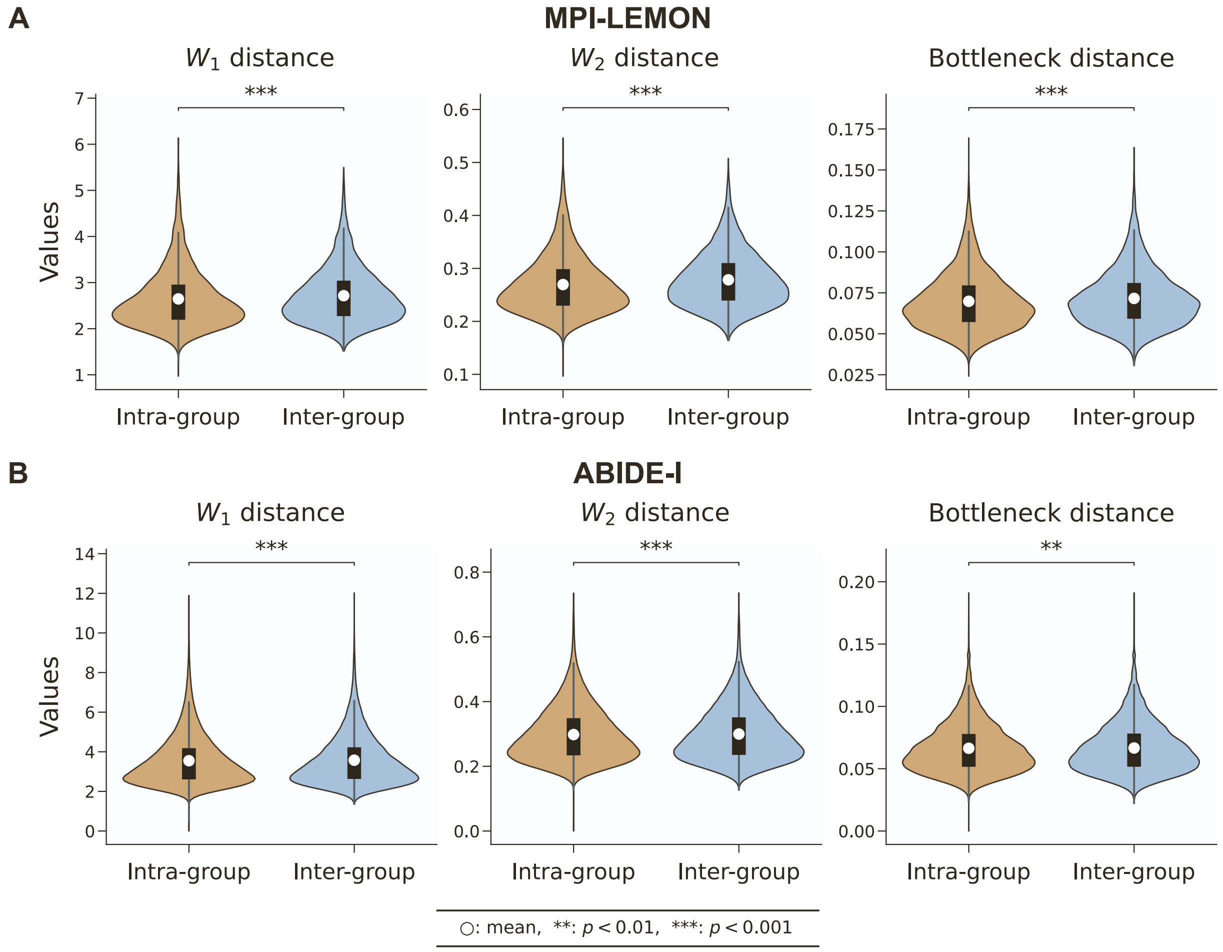

Figure S2: **Brain-wide differences between the intra-group and inter-group distances of persistent diagrams as identified by three global measures: 1-Wasserstein (W_1), 2-Wasserstein (W_2), and bottleneck distances, considering only the positive correlations of the functional connectivity matrices.** (A) MPI-LEMON dataset: violin plots corresponding to intra-group (young-young or elderly-elderly pairs) and inter-group (young-elderly pairs) distances across three measures. The mean values of inter-group distances are significantly higher ($p < 0.001$) than the intra-group distances. (B) ABIDE-I dataset: violin plots corresponding to intra-group (ASD-ASD or TD-TD pairs) and inter-group (ASD-TD pairs) distances across three global measures. The mean values of inter-group distances are significantly higher ($p < 0.01$) than the intra-group distances.

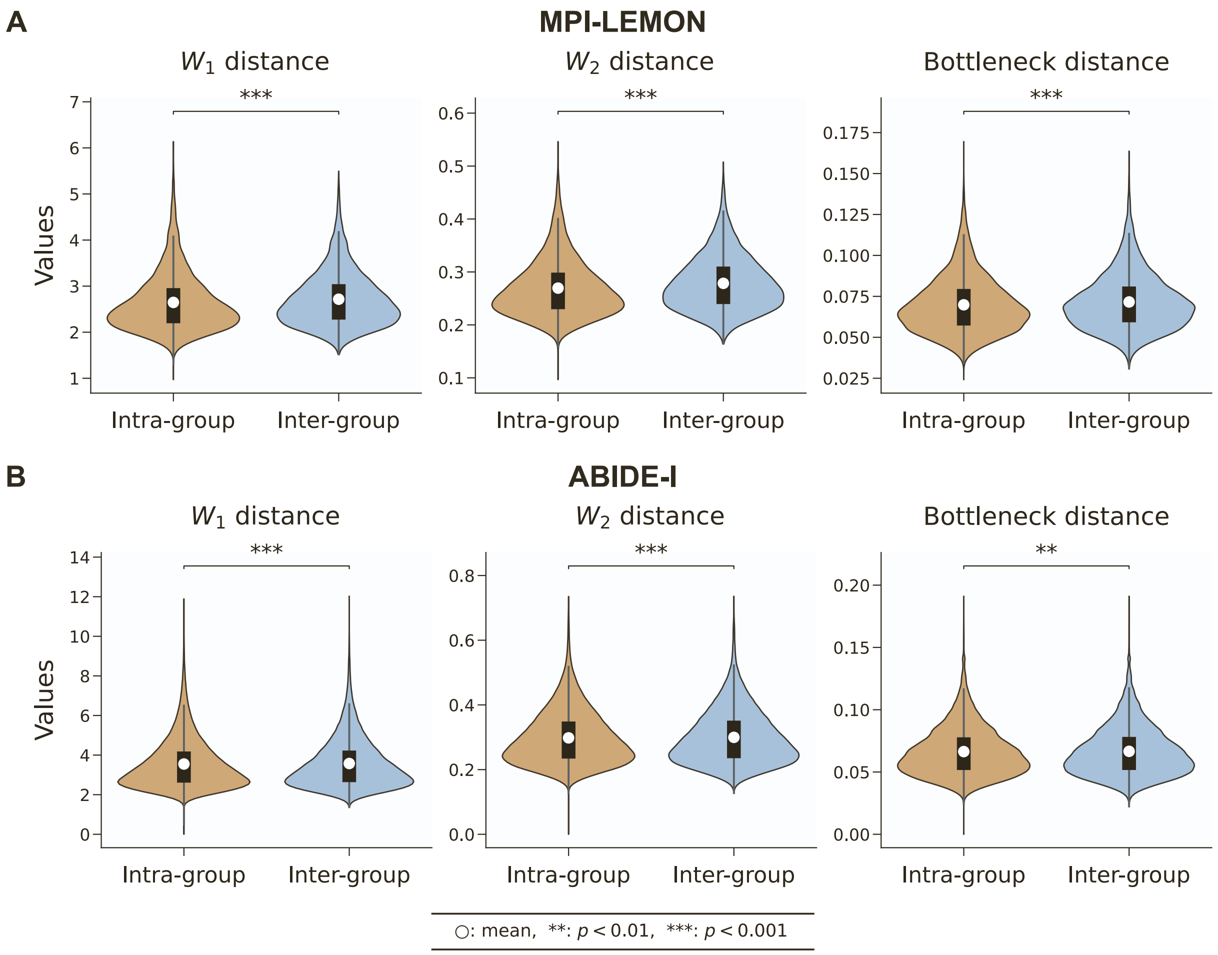

Figure S3: **Brain-wide differences between the intra-group and inter-group distances of persistent diagrams as identified by three global measures: 1-Wasserstein (W_1), 2-Wasserstein (W_2), and bottleneck distances, considering all the correlations of the functional connectivity matrices.** (A) MPI-LEMON dataset: violin plots corresponding to intra-group (young-young or elderly-elderly pairs) and inter-group (young-elderly pairs) distances across three measures. The mean values of inter-group distances are significantly higher ($p < 0.001$) than the intra-group distances. (B) ABIDE-I dataset: violin plots corresponding to intra-group (ASD-ASD or TD-TD pairs) and inter-group (ASD-TD pairs) distances across three global measures. The mean values of inter-group distances are significantly higher ($p < 0.01$) than the intra-group distances.

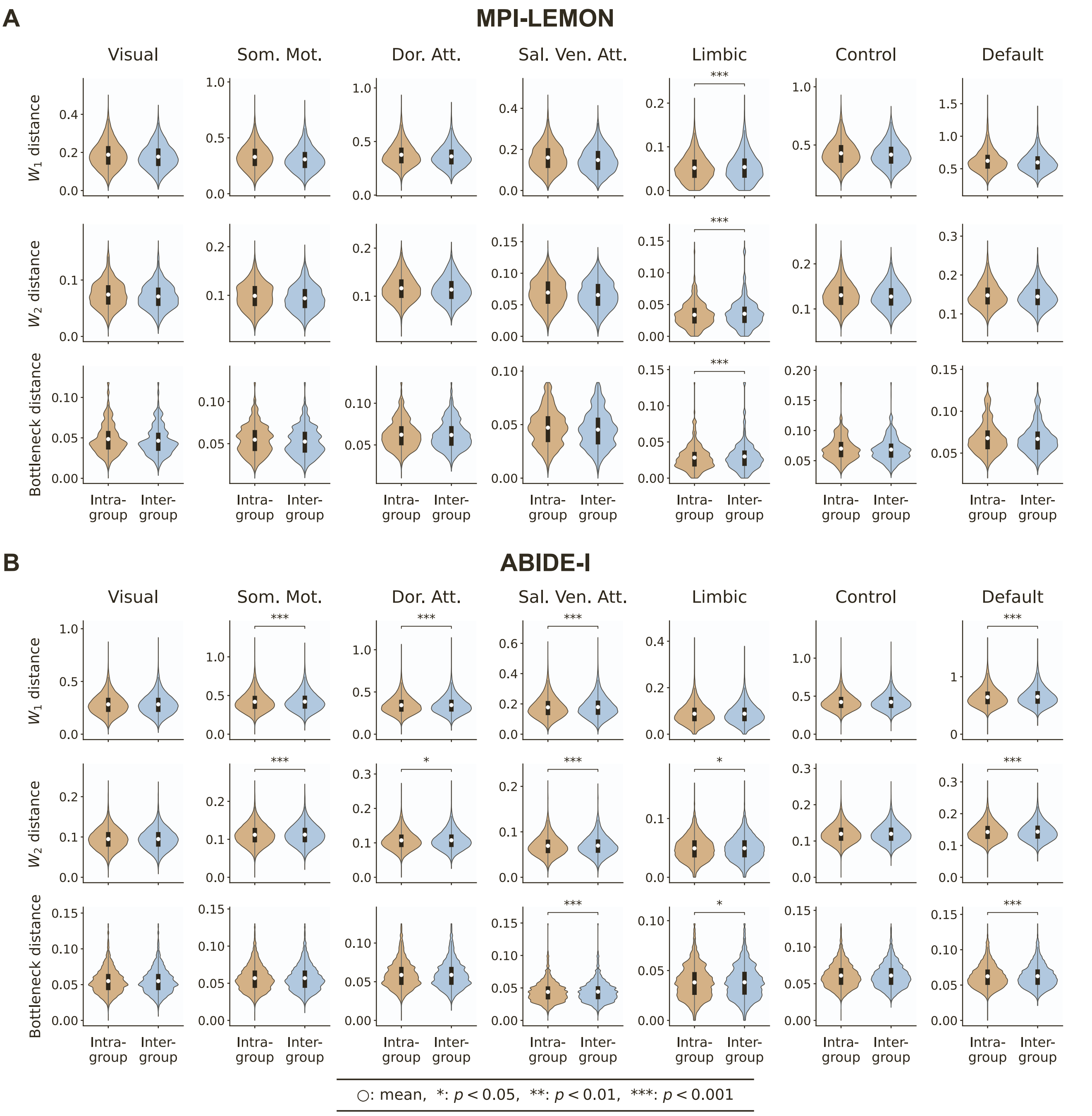

Figure S4: **RSN-level differences between the intra-group and inter-group distances of the persistent diagrams as identified by three global measures: 1-Wasserstein (W_1), 2-Wasserstein (W_2), and bottleneck distances, considering only the positive correlations of the functional connectivity matrices.** Each row corresponds to a given measure, and each column corresponds to a given RSN. (A) MPI-LEMON dataset: violin plots corresponding to intra-group (young-young or elderly-elderly pairs) and inter-group (young-elderly pairs) distances. Inter-group distances are significantly higher ($p < 0.001$) than the intra-group distances only for the limbic network corresponding to all three measures. (B) ABIDE-I dataset: violin plots corresponding to intra-group (ASD-ASD or TD-TD pairs) and inter-group (ASD-TD pairs) distances. For the somatomotor (Som. Mot.) and dorsal attention (Dor. Att.) networks, the inter-group distances are significantly higher ($p < 0.05$) than the intra-group distances for 1-Wasserstein and 2-Wasserstein distances. In the salience/ventral attention (Sal. Ven. Att.) and default networks, inter-group distances are significantly higher ($p < 0.001$) than the intra-group distances for the three measures: 1-Wasserstein, 2-Wasserstein and bottleneck distances. In the limbic network, inter-group distances are significantly higher ($p < 0.05$) than the intra-group distances for 2-Wasserstein and bottleneck distances.

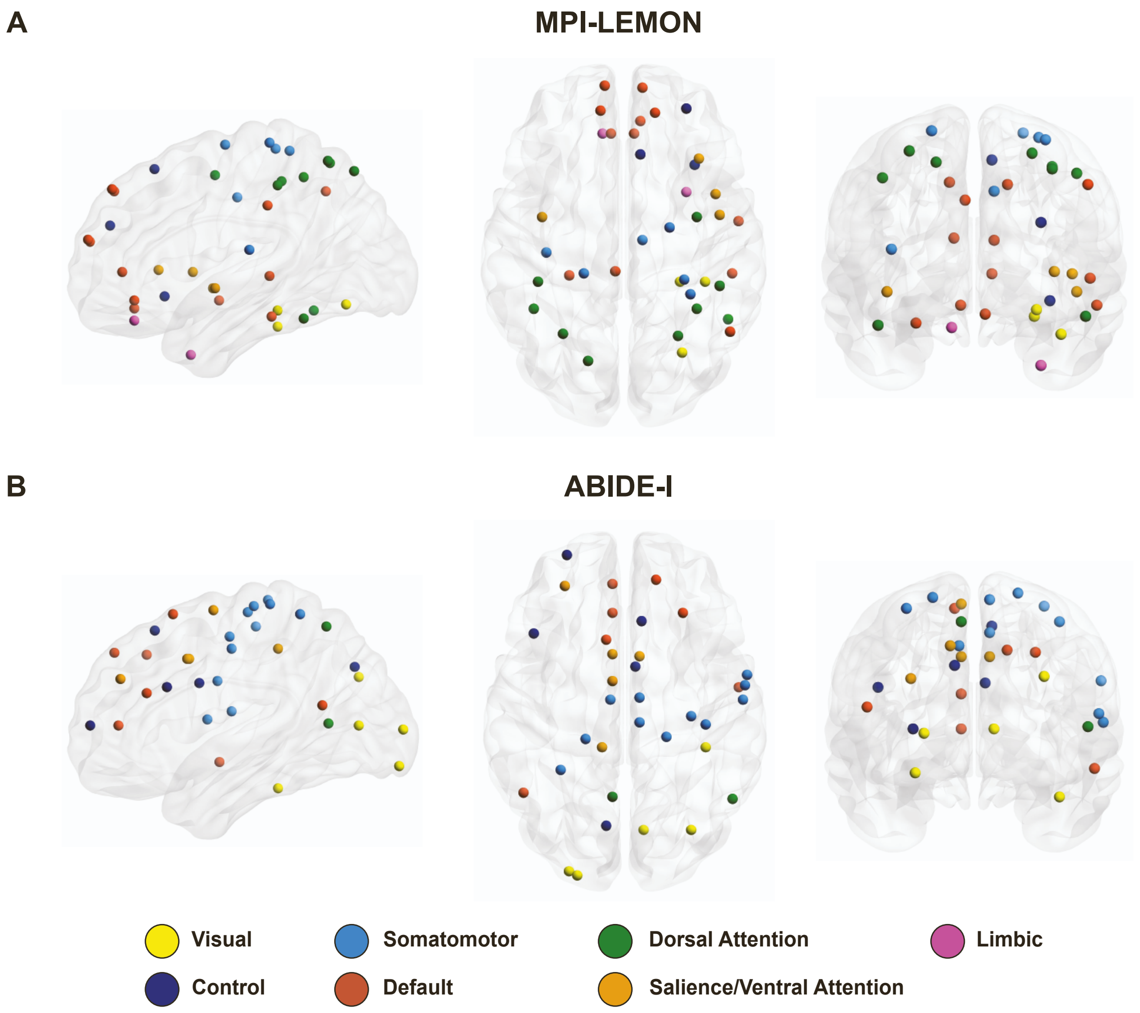

Figure S5: **Visual representation of brain regions with significant between-group differences in node frequency ($p < 0.05$, FDR-corrected).** (A) MPI-LEMON dataset: 39 regions with significant differences in node frequency between the healthy young and healthy elderly groups. For every region, young participants show higher node frequency compared to the elderly participants, except one region RH_Limbic_TempPole_1. (B) ABIDE-I dataset: 35 regions with significant differences in node frequency between the autism spectrum disorder (ASD) and typically developing (TD) groups. All regions reveal increased node frequency for ASD participants relative to TD participants. Each brain region is assigned to one of the seven resting-state networks (RSNs) as defined by the Schaefer atlas. The regions are colored according to their respective RSNs, as detailed in the figure legend. The visualization was generated using BrainNet Viewer. Table S4 lists the significantly different ROIs identified via node frequency across both datasets.

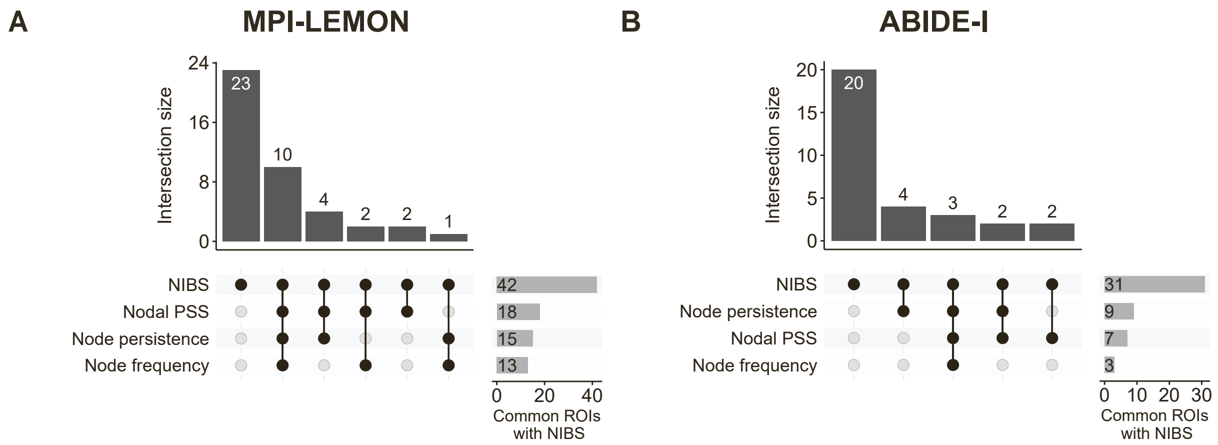

Figure S6: **UpSet plots illustrating the intersection of ROIs identified via non-invasive brain stimulation (NIBS), and local persistent homology-based measures from RSN-to-local level analysis.** Each subplot highlights the combinations of node-based measures, namely node persistence, node frequency, and nodal PSS, with a focus on NIBS-identified regions. The connected cells in the matrix indicate the sets of ROIs involved in each intersection, and the bars aligned with the columns represent the size of each intersection, providing a visual representation of how the sets overlap, particularly emphasizing the role of NIBS in these intersections. The right horizontal bars indicate the cardinality of the intersection of the set of ROIs identified via NIBS and corresponding local persistent homology-based measures. (A) MPI-LEMON dataset: within NIBS, 19 of 42 ROIs are identified considering all three measures collectively, and 10 ROIs are identified when all three are considered simultaneously. Two ROIs are uniquely identified using nodal PSS. (B) ABIDE-I dataset: within the 31 ROIs in NIBS, 11 ROIs are identified considering all three measures collectively, and three ROIs are common across all the measures. Four and two ROIs are uniquely identified considering node persistence and nodal PSS, respectively.

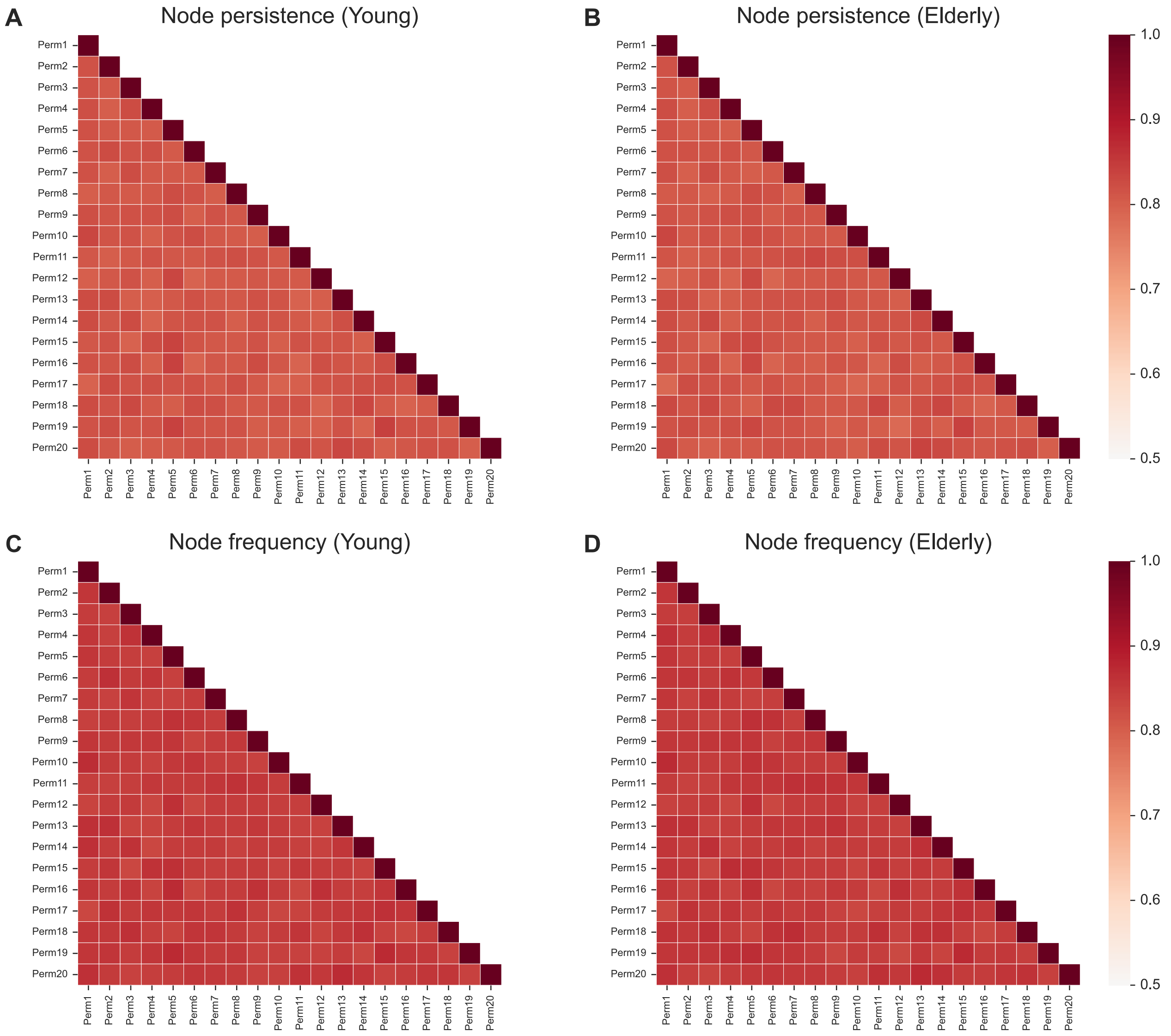

Figure S7: **Pairwise Spearman correlations of the PH-based local measures across 20 random node permutations.** For each permutation, node persistence and node frequency were computed for all nodes in every individual, and subsequently, averaged across individuals within young and elderly groups in the MPI-LEMON dataset. Each subplot shows a 20×20 heatmap corresponding to one measure and one group: (A) node persistence in the young group, (B) node persistence in the elderly group, (C) node frequency in the young group, and (D) node frequency in the elderly group. In all instances, high correlations ($\rho \geq 0.8$) were observed, indicating that node rankings largely remain consistent irrespective of representative cycle choice.

Supplemental notes

Note S1. Correlation of topological measures with phenotypic and clinical scores

We conducted a correlation analysis to examine the relationship of PH-based metrics with phenotypic test scores for the MPI-LEMON dataset and clinical scores of symptom severity for individuals with ASD in the ABIDE-I dataset. This correlation analysis was performed for topological measures on all three scales (see Supplemental methods).

MPI-LEMON dataset provides 52 affective processing-related scores corresponding to all 225 subjects. These scores are divided across eight tests: (a) Cognitive Emotion Regulation Questionnaire (CERQ, 9 scores), (b) Coping Orientations to Problems Experienced (COPE, 14 scores), (c) Emotion Regulation Questionnaire (ERQ, 2 scores), (d) Measure of Affect Regulation Style (MARS, 6 scores), (e) Perceived Stress Questionnaire (PSQ, 5 scores), (f) State-Trait-Angstinventar (STAI-G-X2, 1 phenotypic score), (g) Trait Emotional Intelligence Questionnaire-Short Form (TEIQue-SF, 5 scores), and (h) Trierer Inventar zum Chronischen Stress (TICS, 10 scores).

In the MPI-LEMON dataset, we found that PH-based measures exhibit significant correlations ($p < 0.05$, FDR-corrected) with TICS scores at the global level as well as at the level of RSNs. At the global level, persistent entropy shows significant correlations with 6 out of 10 phenotypic TICS scores, while the L^1-norm and L^2-norm show significant correlations with 1 and 2 scores, respectively. At the RSN-level, these measures show significant correlations with TICS scores within the visual, dorsal attention, and salience/ventral attention networks. Specifically, in the visual network, persistent entropy shows significant correlations with 1 out of 10 TICS scores, whereas both L^1-norm and L^2-norm exhibit significant correlations with 9 out of 10 TICS scores. In the dorsal attention network, persistent entropy and the L^1-norm show significant correlations with 1 and 3 out of 10 TICS scores, respectively. In the salience/ventral attention network, persistent entropy is significantly correlated with 5 TICS scores, while the L^1-norm and L^2-norm exhibit significant correlations with 1 and 2 TICS scores, respectively. Table S7 lists all the correlations between 52 affective processing-related scores and three global measures at both the global and RSN levels.

At the ROI-level, age-related differences detected via node persistence in the salience/ventral attention network are related to affective and somatosensory processing (see Figure 5A). Next, we examined the ROI-level correlations by considering the ROIs with significant between-group differences identified via node persistence. In the salience/ventral attention network, 11 such regions were present, which leads to $11 \times 10 = 110$ correlations. Of these, 21 exhibit significant correlations ($p < 0.05$) before FDR correction. These 21 significant correlations are distributed among seven brain regions. After FDR correction, two ROIs LH_SalVentAttn_FrOperIns_1 and RH_SalVentAttn_FrOperIns_3 show significant correlation with node persistence. All the significant correlations are positive, indicating that higher values of PH-based measures are associated with higher levels of chronic stress. Table S8 provides the ROIs that exhibit significant correlations between all the test scores and node persistence.

In the ABIDE-I dataset, we identified two clinical scores based on the Autism Diagnostic Interview-Revised (ADI-R) criteria that assess symptom severity in individuals with ASD: (a) verbal and (b) social scores. Our analysis revealed no significant correlations between topology-based measures and clinical scores at the global or RSN-level. Correlations between the two clinical scores and the three PH-based measures at both global and RSN-level are provided in Table S9. We further explored ROI-level correlations by focusing on ROIs that showed significant between-group differences based on node persistence. No significant correlations were observed between clinical scores and node persistence at the ROI-level (see Table S8).

Note S2. RSN-to-local level analysis

We computed node persistence and node frequency using Rips complexes constructed from submatrices of the FC Matrix at the level of individual RSNs. A two-tailed two-sample t-test was utilized to detect statistical differences between the groups, and FDR correction was applied independently for each RSN.

In the MPI-LEMON dataset, 59 ROIs exhibit significant differences ($p < 0.05$, FDR-corrected) between the young and elderly groups considering node persistence. These ROIs are distributed among six RSNs: visual (1), somatomotor (24), dorsal attention (5), salience/ventral attention (1), control (1), and default (27) networks. All of these ROIs show higher node persistence values in young individuals compared to elderly individuals. In the ABIDE-I dataset, 53 ROIs exhibit significant differences ($p < 0.05$, FDR-corrected) between the ASD and TD groups via node persistence. These ROIs are distributed among the three RSNs: somatomotor (10), salience/ventral attention (5), and default (38) networks. All the ROIs show higher node persistence values for individuals with ASD compared to TD individuals.

Node frequency reveals 40 and 35 ROIs with significant between-group differences ($p < 0.05$, FDR-corrected) for the MPI-LEMON and ABIDE-I datasets, respectively. In the MPI-LEMON dataset, 40 ROIs are distributed in the RSNs as follows: somatomotor (15), dorsal attention (4), salience/ventral attention (3), control (2), and default (16) networks. Moreover, 37 out of these 40 ROIs identified by node frequency are also identified via node persistence. In the ABIDE-I dataset, 35 ROIs are distributed in three RSNs as follows: somatomotor (8), salience/ventral attention

(8), and default (19) networks. However, 31 out of these 35 ROIs overlap with those found via node persistence. All the ROIs show higher node frequency values in young individuals than in elderly individuals within the MPI-LEMON dataset, and in ASD individuals compared to TD individuals within the ABIDE-I dataset.

In the MPI-LEMON dataset, nodal PSS detects 58 ROIs with significant between-group differences ($p < 0.05$, FDR-corrected) between young and elderly individuals. These include ROIs from six RSNs: visual (2), somatomotor (26), dorsal attention (5), salience/ventral attention (7), control (2), and default (16) networks. Of these 58 ROIs, 47 were also identified via node persistence, and 36 were identified via node frequency. However, in this case, 37 out of 58 ROIs show higher nodal PSS values in young individuals than in elderly individuals. In the ABIDE-I dataset, nodal PSS detects 45 ROIs from the three RSNs, somatomotor (7), salience/ventral attention (14), and default (24), with significant differences between the ASD and TD group ($p < 0.05$, FDR-corrected). Of these 45 ROIs, 35 are also identified through node persistence, and 31 ROIs are identified through node frequency. In this case, most of the ROIs (42 out of 45) display higher nodal PSS values in the ASD group compared to the TD group.

All ROI-level differences identified using node persistence, node frequency, and nodal PSS for both datasets are listed in Table S11. Additionally, Table S12 provides the group-wise averages of node persistence, node frequency, and nodal PSS for each of the 200 ROIs across both datasets, along with the corresponding FDR-corrected p-values.

Note S3. Linking RSN-to-local level analysis with non-invasive brain stimulation outcomes

We investigated the relevance of ROI-level differences in PH-based local measures with the existing literature on non-invasive brain stimulation (NIBS) in healthy elderly individuals and individuals with ASD. As mentioned in the Results section in the main text, target regions that have been shown to enhance motor performance in healthy elderly individuals[S1] correspond to 42 ROIs in the Schaefer atlas. Furthermore, 31 Schaefer ROIs show evidence for improving behavioral or cognitive symptoms associated with ASD[S2].

The UpSet plots in Figure S6 illustrate the number of ROIs associated with clinical improvement and their overlaps with ROIs detected by node persistence, node frequency, and nodal PSS, for both the MPI-LEMON and ABIDE-I datasets. For the MPI-LEMON dataset, out of the 42 clinically relevant ROIs identified by NIBS, 19 are captured by at least one of the topology-based local measures, and 10 are identified by all three measures. In particular, node persistence reveals 15 clinically relevant ROIs spanning three RSNs, node frequency identifies 13 ROIs spanning five RSNs, and nodal PSS reveals 18 ROIs spanning five RSNs (see Figure S6A). For the ABIDE-I dataset, out of the 31 clinically relevant ROIs identified by NIBS, 11 are captured by at least one of the three topology-based local measures, and those are distributed in three RSNs: somatomotor, salience/ventral attention, and default networks. Among these 11 ROIs, 4 ROIs are uniquely identified by node persistence, whereas 2 are uniquely identified by nodal PSS. Specifically, node persistence detects 9 clinically relevant ROIs, node frequency identifies 3, and nodal PSS identifies 7 ROIs (see Figure S6B and Table S10).

Note S4. Robustness of the PH-based local measures

We investigated the robustness of the PH-based local measures by considering different representative cycles of one-dimensional holes. To obtain alternative representations of one-dimensional holes, we applied 20 different permutations of node orderings and rearranged the FC matrices accordingly. For each subject, node persistence and node frequency were then recalculated based on the permuted matrices. We calculated two robust variability indices, median absolute deviation (MAD) and its normalization with respect to the median (MAD/M), to quantify the variability of node-level measures arising from different choices of representative cycles. These indices were calculated separately for each subject and each node. Subsequently, for each group, we computed the average, first quartile (Q1), median (Q2), and third quartile (Q3) of MAD and MAD/M across all subjects.

For node persistence, in the young group, a large proportion of nodes exhibit low variability (see Table S13). Specifically, 140, 198, 174, and 92 nodes (out of 200) have average, Q1, Q2, and Q3 of MAD values below 0.1, respectively, whereas 197, 200, 199, and 170 nodes are below 0.2. In the elderly group, the corresponding numbers are slightly higher, with 173, 200, 189, and 122 nodes falling under the 0.1 threshold. For MAD/M, in the young group, 154, 200, 194, and 88 nodes; and in the elderly group, 164, 200, 193, and 103 nodes have average, Q1, Q2, and Q3 values below 0.2, respectively. For node frequency, the variability assessed using MAD remained low in both groups (see Table S13). In the young group, 183, 200, 198, and 177 nodes have average, Q1, Q2, and Q3 values below 1, respectively. Similarly, in the elderly group, 189, 200, 199, and 181 nodes are below this threshold. Since node frequency can only take integer values, these findings suggest that, across different representative cycle choices, most nodes deviated by at most one unit. These highlight the stability of this measure for most of the nodes. For MAD/M, in the young group, 177, 200, 198, and 123 nodes have average, Q1, Q2, and Q3 values below 0.2, respectively. while in the elderly group, 179, 200, 198, and 128 nodes have values less than 0.2. Therefore, MAD/M values are also consistently low, with the majority of nodes falling below 0.2. Since lower MAD and MAD/M values

reflect higher stability, these results indicate that node persistence and node frequency are largely robust across different representative cycle choices for the majority of nodes. However, a few nodes exhibited MAD/M values exceeding 0.2, suggesting that their node-level scores are more sensitive to the choice of representative cycles.

At the RSN-to-local level, robustness analyses were performed for the three local measures: node persistence, node frequency, and nodal PSS. Here as well, we applied 20 different permutations of node orderings separately for each RSN and rearranged the FC matrices accordingly to obtain different representative cycles. Table S15 provides the average, median (Q2), and third quartile (Q3) of MAD and MAD/M across all subjects, separately for each group and each measure, for all the nodes.

For node persistence, the majority of nodes exhibited no variability at the median level (see Table S15), with 197 and 162 nodes in the young group, and 200 and 176 nodes in the elderly group, showing Q2 and Q3 of MAD values of zero, respectively. A similar trend was observed for MAD/M, where 196 and 148 nodes in the young group, and 198 and 167 nodes in the elderly group, have Q2 and Q3 values equal to zero. Considering average values, 188 nodes in the young group and 194 nodes in the elderly group have MAD values below 0.05, while 154 and 163 nodes in the respective groups have average MAD/M values below 0.2. Similarly, for node frequency, most nodes showed no variability at the median level, with Q2 MAD values of zero in 198 nodes (young) and 200 nodes (elderly), and Q3 MAD values of zero in 171 and 178 nodes, respectively. A similar pattern was observed for MAD/M, where 197 (young) and 199 (elderly) nodes have Q2 values of zero, and 154 and 168 nodes have Q3 values of zero. On average, 187 (young) and 189 (elderly) nodes have MAD values less than 0.3, while nearly all nodes (199 young, 197 elderly) have MAD/M values below 0.2. Similar patterns were observed for nodal PSS. In the young group, 196 and 168 nodes have Q2 and Q3 MAD values equal to zero, respectively, and 191 nodes showed an average MAD below 20. In the elderly group, 199 and 178 nodes have Q2 and Q3 MAD values equal to zero, with 198 nodes showing an average below 20. For MAD/M, 195 and 152 nodes in the young group, and 198 and 168 nodes in the elderly group, have Q2 and Q3 values of zero, respectively. Almost all nodes exhibited low variability, with 197 (young) and 195 (elderly) nodes showing average MAD/M values below 0.2. In summary, the RSN-to-local level analysis shows that node persistence, node frequency, and nodal PSS remain stable under different representative cycle selections for almost all nodes.

Note S5. Robustness of the group-level findings

We analyzed the results for all of the 20 node permutations and identified significantly different regions between the young and elderly groups in the MPI-LEMON dataset for each case. For each permutation, a two-tailed two-sample t-test was used to evaluate group-level differences, and FDR correction was applied to adjust for multiple comparisons.

In our analysis, we considered the node order from the Schaefer atlas and reported the corresponding ROI-level results. Using node persistence, we identified 108 regions with significant between-group differences, of which 27 overlapped with NIBS-identified regions (see Table S10). Across 20 random node permutations, the number of significantly different regions ranged from 99 to 116, with an average of 107. Within these, the number of NIBS-identified regions varied between 21 and 31, averaging 25. To assess consistency, we compared the 108 regions identified from the Schaefer node order with those obtained from each of the 20 random permutations by calculating their overlap. The number of overlapping regions ranged from 87 to 94, with an average of 91. For NIBS-identified regions, the overlap ranged from 19 to 25, with an average of 22. Considering node frequency, 39 significant regions were identified, including 10 overlapping with NIBS. Across 20 permutations, the number of significant regions varied between 38 and 55 (average = 45), with NIBS-identified regions ranging from 7 to 12 (average = 9). The overlap with the reported 39 regions ranged from 27 to 34 (average = 32), and for NIBS regions, the range is from 7 to 10 (average = 8).

We further extended this analysis from RSN-to-local level as well, for the three local measures: node persistence, node frequency, and nodal PSS. For each random node permutation, we computed all three measures across the seven RSNs and obtained the total number of significantly different regions by summing over RSNs for that permutation. Considering node persistence, we identified 59 regions with significant group differences, 15 of which coincided with NIBS-identified regions (see Table S10). Across different permutations, the number of significant regions ranged between 49 and 61 (average = 57), while the subset overlapping with NIBS varied between 13 and 18 (average = 15). When comparing the 59 regions identified from the Schaefer node order with those obtained under different permutations, the overlap ranged from 48 to 56 (average = 53). For NIBS-identified regions, consistency was similarly high, with overlaps of 13 to 15 (average = 14). For node frequency, 40 regions showed significant differences, with 13 overlapping NIBS regions. The number of significant regions fluctuated between 32 and 44 (average = 39) across different permutations, with the number of NIBS-identified regions ranging from 7 to 13 (average = 10). Here as well, we compared the 40 regions identified using the Schaefer atlas node ordering with those obtained from each random permutation by calculating their overlap. The overlaps ranged from 31 to 37, with an average of 33. For NIBS-identified regions, the overlap ranged from 7 to 11, with an average of 9. Employing nodalPSS, 58 regions demonstrated significant between-group differences, with 18 overlapping with NIBS-identified regions. Within 20

random permutations, the number of significant regions ranged from 42 to 59, averaging 52. Here, NIBS-identified regions varied between 13 and 20, with an average of 16. Compared to the node ordering of the Schaefer atlas's 58 significant regions, overlaps ranged between 39 and 48 (average = 44), while NIBS overlaps ranged from 12 to 15 (average = 13). Table S14 summarizes the number of significantly different regions identified using node persistence, node frequency, and nodal PSS across the 20 permutations of node ordering, along with their overlap with NIBS-identified regions and with the regions obtained using the Schaefer atlas node order.

Supplemental methods

Simplicial complex

A simplicial complex is a higher-dimensional generalization of a graph, constructed by combining vertices, edges, triangles, tetrahedra, and their higher-dimensional counterparts. Mathematically, a simplicial complex is defined as a collection K of non-empty subsets of a finite set V that satisfies the following two conditions:

1. If $\sigma \in K$ and $\tau \subsetneq \sigma$, then τ must also be an element of K.
2. For each $v \in V$, the singleton $\{v\}$ is included in K.

The elements of V are called the *vertices* of K, while the elements of K are referred to as *simplices*. If $\tau \subsetneq \sigma$ for simplices $\tau, \sigma \in K$, then τ is termed a *face* of σ and denoted as $\tau < \sigma$. A simplex containing $p+1$ elements is known as a p-*simplex*, meaning it has dimension p. The set of all p-simplices in K is denoted by K_p. The *dimension* of the simplicial complex K is defined as the highest dimension among its simplices. To explore simplicial complexes in greater detail, we recommend standard texts[S3,S4] in algebraic topology.

Vietoris-Rips complex

Let (X, d) be a metric space and S a finite set of points in the metric space, often referred to as a point cloud. For a given radius ϵ (> 0) the Vietoris–Rips complex or Rips complex $R_\epsilon(S)$ is a simplicial complex made up of vertices from S, with simplices formed from finite subsets of S, where each pair of points in a given simplex has a distance at most 2ϵ. Formally, Rips complex $R_\epsilon(S)$ is defined as,

$$R_\epsilon(S) = \{\sigma \subseteq S \mid d(p, q) \leq 2\epsilon, \; \forall \, p, q \in \sigma\}$$

where $d(p, q)$ denotes the distance between points p and q. A 1-simplex (or edge) between two points is added if the distance between them is $\leq 2\epsilon$. Given three points, a 2-simplex (or filled triangle) is formed if the pairwise distances between them are all less than or equal to 2ϵ. In the case of higher-dimensional simplices, a k-simplex arises from a subset of $(k + 1)$ points in S if every pair of points in the subset is connected by an edge.

Rips complex is an efficient method that enables the construction of a simplicial complex on an arbitrary space. Besides Rips complex, there are other methods available, such as Čech complex and alpha complex to construct a simplicial complex[S5]. However, Rips complex is more computationally efficient than Čech complex and is often regarded as an approximation of the latter[S6–S8]. The efficiency arises from the simplicity of its construction, as determining whether a simplex $\sigma \subseteq S$ belongs to $R_\epsilon(S)$ requires only the computation of pairwise distances. For more details regarding Rips complex see e.g.[S9,S10].

Filtration of simplicial complexes

As mentioned, a simplicial complex K is a set of a finite number of simplices, and the simplices are either disjoint or intersect in a common face. A subcomplex is a subset $L \subseteq K$ of the set of simplices such that all the elements in L also satisfy the properties of a simplicial complex.

Given a simplicial complex K, a filtration of length n is a nested sequence of subcomplexes, where each subcomplex is contained within the next. Formally, it is defined as:

$$K_0 \subseteq K_1 \subseteq K_2 \subseteq \cdots \subseteq K_n = K.$$

Here, K_i represents a simplicial complex at the ith stage.

The Rips complex defined in the Methods section admits a natural filtration of simplicial complexes where each subcomplex is indexed by a real parameter ϵ (radius), corresponding to half the distance between pairs of points. Given that only a finite number of point pairs exist, there will be finitely many ϵ values at which new simplices get introduced. At the beginning of the filtration process, i.e., when ϵ is very small, the Rips complex contains only the set of points K_0 in a given metric space. Next, the radius ϵ around each point is gradually increased, and at each

step of the filtration, a simplicial complex is formed. As the radius ϵ increases, higher-dimensional simplices such as edges, triangles, and tetrahedra form. For large ϵ, all points become interconnected, forming a single large simplex. In summary, a filtration tracks how topological features (such as connected components, loops, and voids) evolve across different scales.

Homology

The construction of homology groups of a simplicial complex K begins with a p-chain. A p-chain C_p for an oriented simplicial complex K is defined by the formal linear combinations of oriented p-simplices α_i of K, expressed as:

$$C_p = \sum_{i=1}^{N} c_i \alpha_i$$

where the coefficients c_i are elements from a base field F. Under pointwise addition, the family of p-chains forms a group over the base field F, called the p-dimensional chain group of K, and this group is denoted by $C_p(K)$.

Next, the boundary operator $\partial_p : C_p \to C_{p-1}$ maps the p-chain to the sum of the $(p-1)$-dimensional faces of that p-simplex resulting in a $(p-1)$-chain. Let $\alpha = [x_0, x_1, \ldots, x_p]$ be an oriented p-simplex, and let $[x_0, \ldots, \hat{x}_i, \ldots, x_p]$ denote the $(p-1)$-simplex after the removal of the point x_i from that p-simplex. Then the boundary operator ∂_p is defined as follows:

$$\partial_p(\alpha) = \sum_{i=0}^{p} (-1)^i [x_0, \ldots, \hat{x}_i, \ldots, x_p]$$

The boundary operator satisfies the fundamental property $\partial_p \circ \partial_{p+1} = 0$, which ensures that the boundary of a boundary vanishes. Now, sequentially arranging the chain groups and boundary operators yields a chain complex:

$$\cdots \xrightarrow{\partial_{p+2}} C_{p+1} \xrightarrow{\partial_{p+1}} C_p \xrightarrow{\partial_p} C_{p-1} \xrightarrow{\partial_{p-1}} \cdots$$

Next, p-cycles and p-boundaries are defined from the boundary operator ∂_p. The kernel of the boundary operator ∂_p is called a p-cycle and is denoted by Z_p, i.e.

$$Z_p = \ker(\partial_p) = \{c \in C_p \mid \partial_p(c) = 0\}.$$

In other words, a p-cycle is a p-chain with an empty boundary. A p-boundary is a p-cycle that lies in the image of the boundary operator ∂_{p+1}. The p-boundaries form a group B_p, which is a subgroup of the p-cycles Z_p[S3], i.e.

$$B_p = \mathrm{img}(\partial_{p+1}) = \{c \in C_p \mid \exists\, b \in C_{p+1},\, \partial_{p+1}(b) = c\}.$$

Thus, the simplicial p-homology group of K is defined by the quotient group,

$$H_p(K) = \frac{Z_p(K)}{B_p(K)}.$$

Since the coefficient field is commutative, B_p is a normal subgroup, and H_p is also a group.

The elements of the p-homology group H_p are informally known as p-holes. Notably, H_p forms a vector space over the field F and the dimension of H_p defines β_p, the p-Betti number. Intuitively, this Betti number provides the number of holes formed by p-simplices. For instance, β_0 represents the number of connected components, β_1 represents the number of loops or one-dimensional holes, β_2 represents the number of voids or cavities or two-dimensional holes, and this pattern continues for higher dimensions.

Persistent homology

In the filtration of a simplicial complex K, each subcomplex K_i contains p-chains, p-cycles, p-boundaries, and a p-boundary operator acting on the p-chains. Therefore, the j-persistent p-homology of K_i is defined as,

$$H_p^{i,j} = \frac{Z_p^i}{B_p^{i+j} \cap Z_p^i}$$

where Z_p^i denotes the p-cycles in the subcomplex K_i, B_p^{i+j} denotes the p-boundaries in K_{i+j}. Therefore, the j-persistent p-Betti number of the subcomplex K_i becomes $\beta_p^{i,j} = \dim(H_p^{i,j})$.

The PH of a filtration of simplicial complexes gives more refined information than just the homology of the individual subcomplexes[S11–S13]. In particular, the PH group $H_p^{i,j}$ contains the p-cycles that form in the subcomplex K_i and become boundaries at the subcomplex K_{i+j}. This indicates that the p-cycle persists from step i to step $i+j$ as it appears (or is born) at K_i and disappears (or dies) at K_{i+j}. In simple words, persistence captures the span of the important topological features from their appearance (birth) to their disappearance (death) across the filtration process. For comprehensive details on PH, refer to[S5,S12–S16].

Persistence diagram and persistence landscape

Like barcodes, a persistence diagram also provides a visual representation of the span of the features. A persistence diagram represents a multiset of points $\{(b_i, d_i)\}$ in $\mathbb{R}^2$, representing the birth and death pairs associated with p-holes[S10]. In this diagram, each point (b_i, d_i) is plotted in the Cartesian plane, where the x and y axes represent filtration values at birth and death, respectively. Since $d_i > b_i$, all points lie above the $b = d$ line, and the farther above, the longer the span of the associated p-hole. Persistence diagrams are robust to noisy data[S17]. Distance functions, such as the Wasserstein distance, can be considered to define a metric space of persistence diagrams[S18,S19].

Next, to perform statistical analysis, a persistence diagram is transformed into a sequence of real-valued functions $\Lambda_i : \mathbb{R} \to \mathbb{R}$ for $i \geq 1$, known as the persistence landscape[S20], aggregating the essential information contained in persistence diagrams. The following method is used to construct a persistence landscape from the persistence diagram. First, a clockwise rotation of $45°$ is applied to convert the diagonal of a persistence diagram into the x-axis. Then, isosceles right triangles are drawn from each point (b_i, d_i) of the persistence diagram, considering the point as the vertex. Next, a tent function is constructed from each of the generated triangles. For instance, the second tent function represents the second-highest value at each point across all triangles. In mathematical terms, a piecewise linear function $\Lambda_i : \mathbb{R} \to [0, \infty)$ exists for every birth–death pair $\{(b_i, d_i)\}$ in the persistence diagram defined as:

$$\Lambda_i(t) = \begin{cases} t - b_i & \text{for } t \in [b_i, \frac{b_i+d_i}{2}] \\ d_i - t & \text{for } t \in [\frac{b_i+d_i}{2}, d_i] \\ 0 & \text{otherwise.} \end{cases}$$

The kth largest value among the set $\{\Lambda_i\}$ defines the persistence landscape $\lambda_k(t)$. In this study, we consider $k = 1$, as $\lambda_1(t)$ typically captures the most prominent topological features for each t. As a subset of a Banach space, the persistence landscape enables the calculation of L^p-norms $(1 \leq p \leq \infty)$, which is not the case for persistence diagrams. The L^p-norms of a persistence landscape $\lambda_1(t)$ are defined as[S21]:

$$\|\lambda_1\|_p = \left(\int_{-\infty}^{\infty} |\lambda_1(t)|^p \, dt \right)^{1/p}.$$

In the case of a single birth-death pair (b, d) in the persistence diagram, the norms of $\lambda_1(t)$ are given by: $\|\lambda_1\|_1 = \frac{1}{4}(d - b)^2$ and $\|\lambda_1\|_2 = \frac{1}{2\sqrt{3}}(d - b)^{3/2}$. For multiple non-overlapping birth-death pairs $\{(b_i, d_i)\}$, the norms are computed as: $\|\lambda_1\|_1 = \sum_i \frac{1}{4}(d_i - b_i)^2$ and $\|\lambda_1\|_2 = \sum_i \frac{1}{2\sqrt{3}}(d_i - b_i)^{3/2}$.

Moreover, we calculate the Wasserstein and bottleneck distances to measure the difference between two persistence diagrams[S10]. Let X and Y denote two persistence diagrams. The p-Wasserstein distance is the minimum total cost required to align points (birth-death pairs) from one diagram with another and is mathematically expressed as:

$$W_p(X, Y) = \left(\inf_{\gamma} \sum_{x \in X} \|x - \gamma(x)\|_\infty^p \right)^{1/p},$$

where γ represents a bijection between the points of X and Y, allowing points to be matched either to each other or, when necessary, to the diagonal, due to the difference in the cardinalities of X and Y. This distance quantifies disparities in topological features between two datasets. The bottleneck distance is a special case of the Wasserstein distance with parameter $p \to \infty$, defined as:

$$W_\infty(X, Y) = \inf_{\gamma} \sup_{x \in X} \|x - \gamma(x)\|_\infty.$$

Correlation of topological measures with cognitive, behavioral, and clinical scores

A correlation analysis was conducted to quantify the strength of the linear association between PH-based measures and (i) performance on cognitive/behavioral tests in the MPI-LEMON dataset, and (ii) clinical scores indicating symptom severity in the ABIDE-I dataset. We examined these correlations across all three spatial scales: (a) global scale (brain-wide changes), (b) mesoscopic scale (RSN-level changes), and (c) local scale (ROI-level changes).

The MPI-LEMON dataset includes comprehensive data on 6 cognitive tests and 21 questionnaires addressing emotional tendencies, personality traits, dietary habits, and addiction, accessible at `https://fcon_1000.projects.nitrc.org/indi/retro/MPI_LEMON.html`. We retrieved the phenotypic test scores corresponding to all 225 individuals. Subsequently, we calculated Spearman correlation of metrics derived from PH: persistent entropy, L^1-norm, L^2-norm, and node persistence, with the phenotypic scores available for each cognitive or behavioral test. We employed Spearman correlation as certain test scores are ordinal rather than continuous. Finally, we adjusted the

p-values associated with the estimated correlations using False Discovery Rate (FDR) correction. The FDR correction was implemented separately for p-values of each cognitive or behavioral test. For example, if a given cognitive or behavioral test contains more than one phenotypic score, we performed the FDR correction across all associated scores.

For the ABIDE-I dataset, the analysis was conducted solely for the ASD group. We opted for two clinical scores according to the Autism Diagnostic Interview-Revised (ADI-R) scoring criteria[S22], namely ADI-R verbal and ADI-R social. Among all the potential clinical scores, the ADI-R scores are particularly noteworthy for two main reasons. First, they are available for the largest segment of the ASD group (275 participants). Second, the ADI-R social and verbal scores provide an effective measure of symptom severity in autism[S23]. We calculated the Spearman correlation between PH-based measures and clinical scores. However, no FDR correction was applied at the global and mesoscopic scales since only two scores were considered.

Supplemental references

S1. Yadav, Y., Elumalai, P., Williams, N., Jost, J., and Samal, A. (2023). Discrete Ricci curvatures capture age-related changes in human brain functional connectivity networks. Frontiers in Aging Neuroscience *15*, 1120846. doi:10.3389/fnagi.2023.1120846.

S2. Elumalai, P., Yadav, Y., Williams, N., Saucan, E., Jost, J., and Samal, A. (2022). Graph Ricci curvatures reveal atypical functional connectivity in autism spectrum disorder. Scientific Reports *12*, 8295. doi:10.1038/s41598-022-12171-y.

S3. Munkres, J. R. Elements of Algebraic Topology. CRC press (2018).

S4. Hatcher, A. Algebraic Topology. Cambridge University Press (2002).

S5. Otter, N., Porter, M. A., Tillmann, U., Grindrod, P., and Harrington, H. A. (2017). A roadmap for the computation of persistent homology. EPJ Data Science *6*, 17. doi:10.1140/epjds/s13688-017-0109-5.

S6. Ghrist, R. (2008). Barcodes: The persistent topology of data. Bulletin of the American Mathematical Society *45*, 61–75. doi:10.1090/S0273-0979-07-01191-3.

S7. Dantchev, S., and Ivrissimtzis, I. (2012). Efficient construction of the Čech complex. Computers & Graphics *36*, 708–713. doi:10.1016/j.cag.2012.02.016.

S8. Kerber, M., and Sharathkumar, R. (2013). Approximate Čech Complex in Low and High Dimensions. In: Algorithms and Computation. Springer Berlin Heidelberg (666–676). doi:10.1007/978-3-642-45030-3_62.

S9. Vietoris, L. (1927). Über den höheren Zusammenhang kompakter Räume und eine Klasse von zusammenhangstreuen Abbildungen. Mathematische Annalen *97*, 454–472. doi:10.1007/BF01447877.

S10. Edelsbrunner, H., and Harer, J. Computational Topology: An Introduction. American Mathematical Society (2010).

S11. Robins, V. (1999). Towards computing homology from finite approximations. In: Topology Proceedings vol. 24. (503–532).

S12. Edelsbrunner, H., Letscher, D., and Zomorodian, A. (2002). Topological Persistence and Simplification. Discrete & Computational Geometry *28*, 511–533. doi:10.1007/s00454-002-2885-2.

S13. Zomorodian, A., and Carlsson, G. (2005). Computing Persistent Homology. Discrete & Computational Geometry *33*, 249–274. doi:10.1007/s00454-004-1146-y.

S14. Edelsbrunner, H., and Harer, J. (2008). Persistent Homology – a Survey. Contemporary Mathematics *453*, 257–282.

S15. Carlsson, G. (2009). Topology and data. Bulletin of the American Mathematical Society *46*, 255–308. doi:10.1090/S0273-0979-09-01249-X.

S16. Pun, C. S., Lee, S. X., and Xia, K. (2022). Persistent-homology-based machine learning: a survey and a comparative study. Artificial Intelligence Review *55*, 5169–5213. doi:10.1007/s10462-022-10146-z.

S17. Cohen-Steiner, D., Edelsbrunner, H., and Harer, J. (2005). Stability of persistence diagrams. In: Proceedings of the Twenty-First Annual Symposium on Computational Geometry. (263–271). doi:10.1145/1064092.1064133.

S18. Mileyko, Y., Mukherjee, S., and Harer, J. (2011). Probability measures on the space of persistence diagrams. Inverse Problems *27*, 124007. doi:10.1088/0266-5611/27/12/124007.

S19. Turner, K., Mileyko, Y., Mukherjee, S., and Harer, J. (2014). Fréchet Means for Distributions of Persistence Diagrams. Discrete & Computational Geometry *52*, 44–70. doi:10.1007/s00454-014-9604-7.

S20. Bubenik, P. (2015). Statistical topological data analysis using persistence landscapes. Journal of Machine Learning Research *16*, 77–102.

S21. Bubenik, P. (2020). The Persistence Landscape and Some of Its Properties. In: Topological Data Analysis. (97–117). doi:10.1007/978-3-030-43408-3_4.

S22. Lord, C., Rutter, M., and Le Couteur, A. (1994). Autism Diagnostic Interview-Revised: A revised version of a diagnostic interview for caregivers of individuals with possible pervasive developmental disorders. Journal of Autism and Developmental Disorders *24*, 659–685. doi:10.1007/BF02172145.

S23. Lefort-Besnard, J., Vogeley, K., Schilbach, L., Varoquaux, G., Thirion, B., Dumas, G., and Bzdok, D. (2020). Patterns of autism symptoms: hidden structure in the ADOS and ADI-R instruments. Translational Psychiatry *10*, 257. doi:10.1038/s41398-020-00946-8.
