# Supplementary material for: Node persistence from topological data analysis reveals changes in brain functional connectivity
Source: Patterns (N Y). 2025 Dec 3;7(3):101427. doi: 10.1016/j.patter.2025.101427 (PMC13100682; doi:10.1016/j.patter.2025.101427)
Supplement: Document S2. Article plus supplemental information [file mmc17.pdf]

Patterns

Article

Node persistence from topological data analysis reveals changes in brain functional connectivity

Graphical abstract

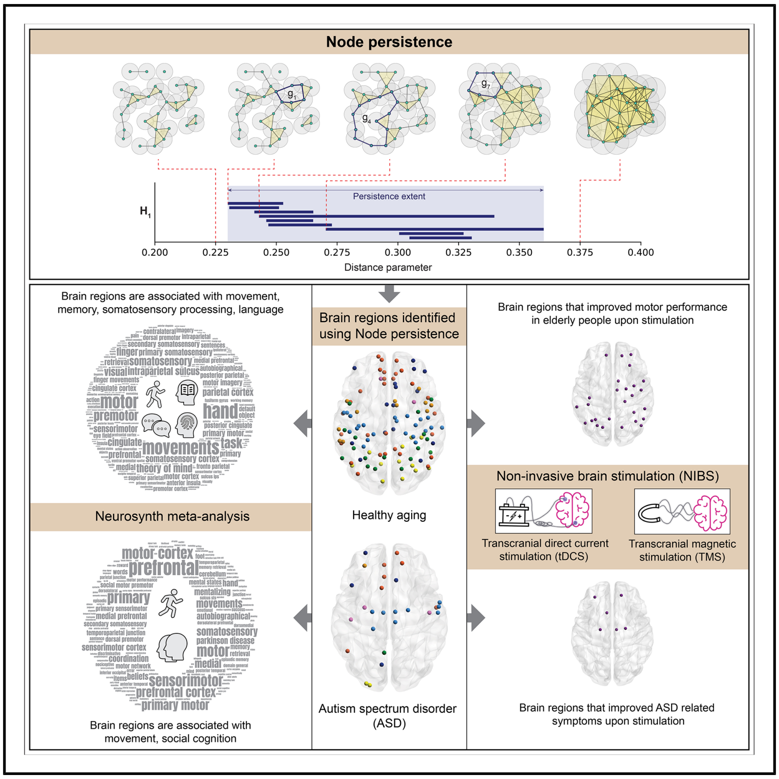

Highlights

- TDA-based investigation of functional connectivity changes in healthy aging and ASD
- Persistent homology characterizes connectivity at global, mesoscopic, and local scales
- Node persistence, a new scalable measure, detects local functional connectivity changes
- Node persistence highlights clinically relevant regions linked to aging and ASD

Authors

Madhumita Mondal, Yasharth Yadav, Jürgen Jost, Areejit Samal

Correspondence

jjost@mis.mpg.de (J.J.),
asamal@imsc.res.in (A.S.)

In brief

This study employs persistent homology to investigate alterations in brain functional connectivity associated with healthy aging and autism spectrum disorder (ASD). Node persistence, a scalable local measure based on persistent homology introduced in this study, identifies brain regions linked to these conditions, including those with clinical evidence from non-invasive brain stimulation.

Mondal et al., 2026, Patterns 7, 101427
March 13, 2026 © 2025 The Author(s). Published by Elsevier Inc.
https://doi.org/10.1016/j.patter.2025.101427

CellPress

Article

Node persistence from topological data analysis reveals changes in brain functional connectivity

Madhumita Mondal,[1,2] **Yasharth Yadav,**[3] **Jürgen Jost,**[4,5,6,7,*] **and Areejit Samal**[1,2,8,*]
[1]The Institute of Mathematical Sciences (IMSc), Chennai, Tamil Nadu 600113, India
[2]Homi Bhabha National Institute (HBNI), Mumbai, Maharashtra 400094, India
[3]School of Physical and Mathematical Sciences, Nanyang Technological University, Singapore 637371, Singapore
[4]Max Planck Institute for Mathematics in the Sciences, Leipzig, Saxony 04103, Germany
[5]Max Planck Institute for Human Cognitive and Brain Sciences, Leipzig, Saxony 04103, Germany
[6]Center for Scalable Data Analytics and Artificial Intelligence Dresden/Leipzig, Leipzig, Saxony 04107, Germany
[7]Santa Fe Institute for the Sciences of Complexity, Santa Fe, NM 87501, USA
[8]Lead contact
*Correspondence: jjost@mis.mpg.de (J.J.), asamal@imsc.res.in (A.S.)
https://doi.org/10.1016/j.patter.2025.101427

THE BIGGER PICTURE Understanding how the human brain's functional connectivity changes with age or in neurological conditions such as autism spectrum disorder (ASD) is crucial for both neuroscience research and potential clinical applications. Large-scale analyses of brain networks can reveal disruptions in regional activity and connectivity patterns that are often associated with cognitive decline, behavioral differences, or neurological disorders. In this study, we provide a comprehensive multiscale view of how brain networks are affected. Importantly, this study introduces a local measure that identifies brain regions with significant differences in connectivity for both healthy aging and ASD. Together, these findings highlight the sensitivity of node persistence to connectivity changes associated with aging and ASD. The broader importance of this research lies in its ability to link mathematical tools with clinically meaningful patterns in brain function. In the long run, the identified regions from the proposed methods may help guide more targeted therapeutic approaches such as non-invasive brain stimulation by highlighting areas most affected by aging or ASD.

SUMMARY

Large-scale analyses of brain functional connectivity can uncover disruptions in regional activity and connectivity that are commonly associated with neurological disorders or cognitive decline associated with healthy aging. In our study, we employ persistent homology (PH), a prominent tool in topological data analysis, to investigate changes in resting-state functional connectivity in healthy aging and autism spectrum disorder (ASD). We analyze functional connectivity changes across three distinct scales: (1) global scale (brain-wide changes), (2) mesoscopic scale (resting-state-network-level changes), and (3) local scale (region-level changes). At the local scale, we introduce node persistence, a scalable PH-based measure that detects brain regions with significant differences in healthy aging or ASD. Notably, these regions overlap with regions whose non-invasive stimulation improves motor function in the elderly or alleviates ASD symptoms, suggesting the utility of node persistence in identifying clinically relevant brain regions affected by aging and ASD.

INTRODUCTION

Brain functional connectivity analysis is a crucial aspect of neuroscience that investigates how different brain regions interact and communicate. Specifically, it examines temporal correlations between neuronal activity across spatially distinct brain regions, revealing activation patterns that occur during cognitive tasks or at rest.[1] Such analyses typically employ neuroimaging techniques such as functional magnetic resonance imaging (fMRI), electroencephalography, or magnetoencephalography. Among these, fMRI is particularly prominent due to its ability to capture spontaneous neuronal activity by measuring fluctuations in blood-oxygen-level-dependent (BOLD) signals. Pairwise correlations between BOLD signal time series across different brain regions[2–4] can reveal functional connectivity networks underlying the brain. Interestingly, spatially distant brain regions demonstrate synchronized activity even in the absence of external cognitive tasks. This phenomenon is known as resting-state functional connectivity and is estimated from resting-state fMRI (rs-fMRI) data.[5–7] Coherent patterns of this

 © 2025 The Author(s). Published by Elsevier Inc.
This is an open access article under the CC BY license (http://creativecommons.org/licenses/by/4.0/).

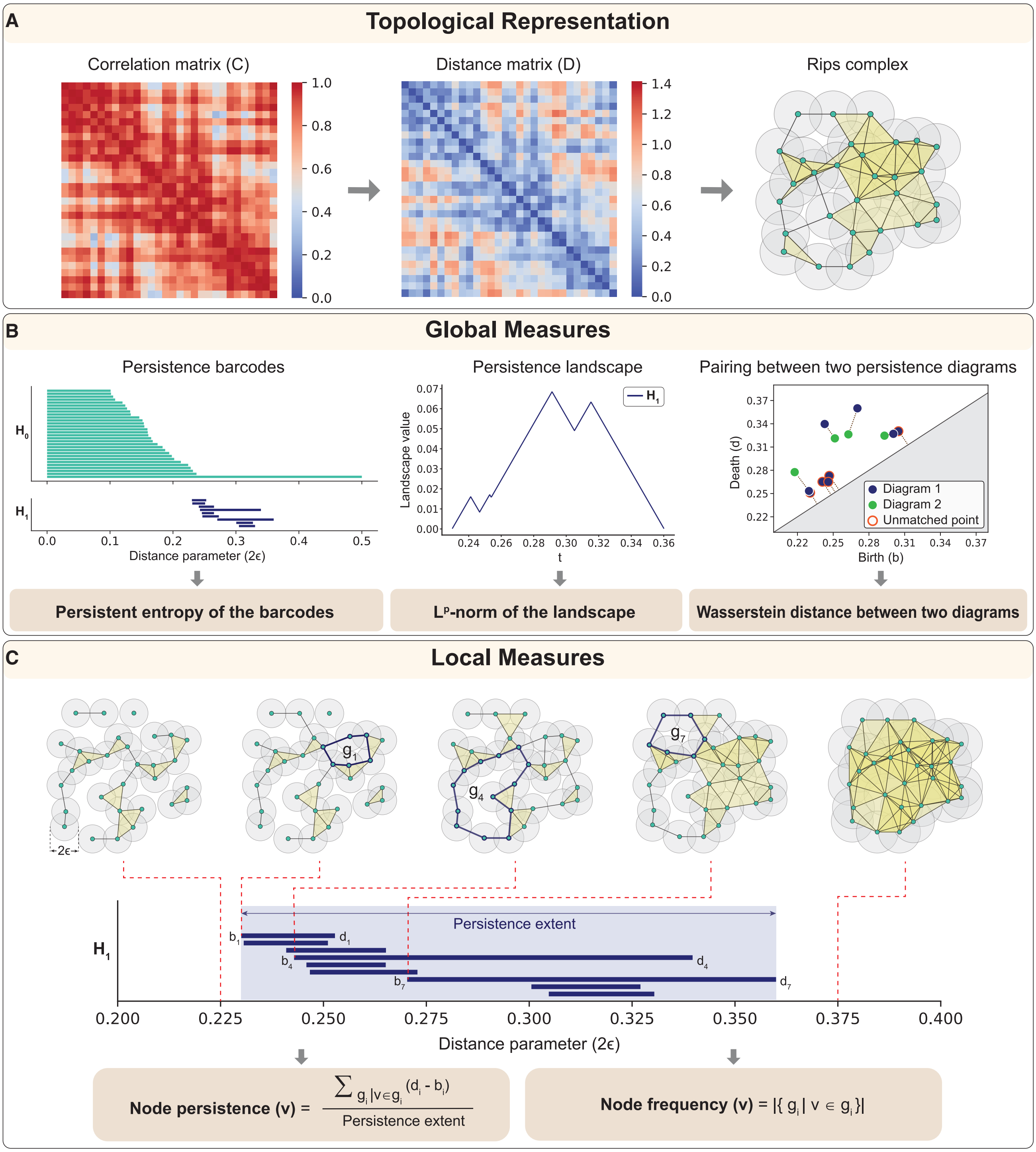

Figure 1. Schematic overview of persistent-homology-based characterization of functional connectivity

(A) Topological representation: from the functional connectivity matrix, which is a type of correlation matrix, the distance matrix ($\boldsymbol{D} = \sqrt{2(1 - \boldsymbol{C})}$) was constructed. Next, this distance matrix was used to build a Rips complex. Note that the Rips complex shown may not reflect the exact spatial embedding of the nodes from the distance matrix.

(B) Global measures: we computed persistent entropy, which quantifies the Shannon entropy of the persistence barcodes, and the L^1-norm and L^2-norm, which capture the overall magnitude of the persistence landscape, to investigate brain-wide and resting-state network-level changes in functional connectivity. Next, we compared the 1-Wasserstein, 2-Wasserstein, and bottleneck distances between intra-group and inter-group persistence diagrams. To compute these distances, points in the diagrams are matched pairwise or with the diagonal if no suitable pairing is possible, and the distances are then calculated based on the lengths of the connecting lines.

(legend continued on next page)

CellPress
OPEN ACCESS

activity across anatomically distinct regions are organized into resting-state networks (RSNs), which reflect the intrinsic functional architecture of the brain. These intrinsic functional networks provide a foundation for understanding large-scale brain organization in both health and disease.[5,7–10] In this study, we focus on studying functional connectivity alterations in two distinct biological processes: age-related cognitive decline and atypical neurodevelopment associated with autism spectrum disorder (ASD).

As the global population ages, uncovering the neural correlates of age-related cognitive decline has become increasingly important. Neuroimaging techniques such as fMRI have been instrumental in advancing our understanding of healthy brain aging.[11,12] Many studies have identified functional changes in regions such as the prefrontal, medial temporal, and parietal cortices, which are essential for maintaining cognitive performance in elderly individuals.[11,13] In parallel, neuroimaging tools have been applied to investigate neurodevelopmental disorders, most notably ASD. A substantial amount of research has investigated the pathophysiology and neurobiology of ASD, providing critical insights into the structural and functional development in ASD relative to typical development.[14,15] ASD refers to a broad range of neurodevelopmental conditions,[16] typically characterized by difficulties in social interaction, verbal or non-verbal communication, and restrictive or repetitive behaviors,[15,16] often accompanied by varying levels of cognitive, motor, or memory impairments.[17–19] The prevalence of ASD is increasing worldwide[20,21] and, while early diagnosis is crucial for effective intervention, it remains equally important to ensure diagnostic reliability and accuracy.[22] Together, these findings underscore the role of neuroimaging techniques, particularly fMRI, in elucidating the neural mechanisms underlying both healthy aging and ASD.[23–25] However, the high complexity and dimensionality of fMRI data have driven the need for advanced analytical tools capable of capturing altered functional connectivity beyond conventional methods. Topological data analysis (TDA) offers a robust multiscale framework for extracting meaningful features from such high-dimensional datasets.[26]

By incorporating principles from algebraic topology and computational geometry, TDA characterizes the inherent shape of high-dimensional datasets.[27–29] In this study, we focus on persistent homology (PH), a central tool in TDA that captures topological features or "holes" in the dataset at multiple scales. These features include connected components (H_0), loops (H_1), and voids (H_2), which correspond to zero-, one-, and two-dimensional holes, respectively. These topological features can then be compactly summarized using representations such as persistence barcodes, persistence diagrams, or persistence landscapes, each capturing the evolution of features across multiple scales.[30–33] Unlike traditional graph-theoretical or network-based methods, which often rely on ad hoc threshold parameters to define connectivity, PH operates across multiple scales, eliminating the need for arbitrary parameter selection. This multiscale framework enables a more robust and unbiased characterization of topological features in complex datasets.[32,34] PH has found widespread application across diverse scientific domains, including biology,[35–38] finance,[39–42] physics,[43–45] and machine learning,[46–48] as well as image and signal processing.[49,50] In particular, PH has found prominent applications in neuroscience due to its ability to encapsulate the multiscale structure of brain connectivity.[51–63] It has been shown to effectively distinguish typical and clinically impaired brain states, providing valuable insights into neurological conditions like ASD,[57,58] Parkinson's disease,[60] Alzheimer's disease,[56,59] and age-related cognitive impairment.[61]

While PH is well suited for summarizing the global topological structure of complex datasets, it lacks the ability to provide local topological information.[31,32] This limitation arises because homology groups are computed on simplicial complexes constructed from the entire dataset, thereby obscuring node-level details. Furthermore, the representatives of homology classes, that is, cycles or holes, are inherently non-unique.[32,64] Nevertheless, understanding how specific brain regions influence global topological changes is crucial in functional connectivity analysis, especially in clinical applications where local alterations often contribute to global functional disruptions.[5,8,10] To address this limitation, several studies have investigated the potential for extracting local topological information from PH.[48,51,52,65–67] In particular, Lord et al.[52] showed that in healthy individuals, topologically central nodes in the persistence scaffold, which summarize one-dimensional holes, may support functional integration across brain modules. Additionally, Liang et al.[57] constructed simplicial complexes at specific thresholds and found significantly fewer one-dimensional holes within certain brain regions in the ASD group compared to the typically developing (TD) controls. However, as data size and dimensionality increase, these methods tend to become increasingly computationally expensive. As a result, local techniques based on PH have seen limited applications in large-scale neuroimaging datasets, highlighting the need for more scalable and efficient methodological advancements.

The primary goal of our work is to develop efficiently computable PH-based metrics and utilize them to identify alterations in the brain functional connectivity in healthy aging and ASD and to link them with cognition and behavior. To this end, we utilized functional connectivity (FC) matrices of 225 subjects from the MPI-LEMON dataset[68,69] and 820 subjects from the ABIDE-I dataset.[70,71] First, we computed PH-based measures at three distinct scales, namely global, mesoscopic, and local, to compare healthy young and healthy elderly individuals in the MPI-LEMON dataset and ASD and TD individuals in the ABIDE-I dataset. At the local or region-level scale, we introduced two PH-based metrics, node persistence and node frequency, which quantify the contribution of a node to one-dimensional homological features. These measures are computationally more efficient than existing local PH-based measures, making them suitable for large-scale brain connectivity analysis. Figure 1 presents an overview of PH-based measures. Second, we utilized Neurosynth meta-analysis[72,73] to identify the cognitive domains

(C) Local measures: we computed node persistence and node frequency to characterize region-level changes in functional connectivity. These measures quantify the influence of a node (v) on one-dimensional homological features (H_1). Rips complexes corresponding to five different distance parameters (2ϵ) are shown, where ϵ denotes the radius of the balls centered at each point, and g_i denotes a representative cycle in H_1 with birth b_i and death d_i.

associated with those brain regions that exhibit significant group differences in our proposed local topological measures. Third, we conducted a correlation analysis to examine the relationship between PH-based measures and phenotypic test scores of individuals in the MPI-LEMON dataset as well as clinical symptom severity scores for individuals with ASD. Fourth, we compared the regions with significant between-group differences in local topological measures against clinically relevant regions documented in existing non-invasive brain stimulation (NIBS) studies. Fifth, we evaluated the effectiveness of our proposed metrics by comparing them with an existing PH-based method for detecting region-level topological changes. Finally, we performed a robustness analysis of the proposed metrics to evaluate their sensitivity to different choices of representative cycles of one-dimensional holes.

RESULTS

In this study, we applied TDA to investigate changes in resting-state functional connectivity associated with healthy aging and ASD. These changes were characterized using topological measures based on PH across three spatial scales: (1) global or brain-wide changes, (2) mesoscopic or RSN-level changes, and (3) local or region of interest (ROI)-level changes. We acquired FC matrices from the MPI-LEMON dataset for the healthy aging investigation and the ABIDE-I dataset for the ASD investigation. The FC matrices in the MPI-LEMON dataset correspond to 153 healthy young and 72 healthy elderly individuals, while those in the ABIDE-I dataset correspond to 395 individuals with ASD and 425 TD individuals. These FC matrices were derived in earlier studies conducted by some of us[69,71] from publicly available resting-state fMRI (rs-fMRI) scans. Each FC matrix is a 200×200 square matrix representing pairwise Pearson correlations between the 200 ROIs, as specified by the Schaefer atlas.[74] Next, we filtered the FC matrices to retain only positive correlations and converted them to ultrametric distance matrices.[75] Our analysis focused on positive correlations, as they have a primary and central role in the structure and organization of brain functional connectivity networks.[76] Finally, we constructed a filtration of Vietoris-Rips (Rips) complexes on each distance matrix and computed PH-based measures to extract multiscale topological information from the FC matrices (Figure 1A). A detailed description of the FC matrices, Rips complex construction, and topological measures is provided in methods and supplemental information.

Brain-wide differences in functional connectivity

We determined brain-wide changes in brain functional connectivity between the groups of individuals using three global topological measures derived from PH, namely persistent entropy,[77] L^1-norm, and L^2-norm[78] (Figure 1B). To evaluate statistical differences between the groups, a two-tailed two-sample t test was conducted.[79]

Figure 2 shows violin plots comparing three global measures between the groups for both MPI-LEMON and ABIDE-I datasets. In the MPI-LEMON dataset, the mean persistent entropy of the young group is significantly higher ($p < 0.001$), suggesting that the persistence of its features is more evenly distributed than the elderly group (Table S1). Furthermore, we found that the young group shows a higher mean L^1-norm ($p < 0.01$) and L^2-norm ($p < 0.001$) of the persistent landscape compared to the elderly group, indicating that one-dimensional holes remain more persistent in the young group. In the ABIDE-I dataset, we found that the mean persistent entropy is significantly higher in the ASD group compared to the TD group ($p < 0.001$). In contrast, the mean L^1-norm and L^2-norm are significantly lower in the ASD group compared to the TD group ($p < 0.001$ for both).

Furthermore, to examine the relationships among the three measures, we performed a Spearman correlation analysis. The analysis was conducted separately for each group, namely young and elderly individuals in the MPI-LEMON dataset and TD and ASD individuals in the ABIDE-I dataset, since each measure showed significant differences between the groups across both datasets. The results revealed a strong positive correlation ($\rho \geq 0.93$) between the L^1-norm and L^2-norm across all groups. In contrast, persistent entropy showed weak positive correlation with either the L^1-norm or L^2-norm. This suggests that the L^1-norm and L^2-norm are largely capturing similar properties, while persistent entropy provides complementary information about the participants. The correlation coefficients, along with the corresponding p values, are provided in Table S2.

The group-wise comparisons presented in Figure 2 correspond to Rips complexes constructed from FC matrices with positive correlations only. We also constructed Rips complexes from FC matrices containing the full range of Pearson correlations and found that the group differences in the three global measures remained consistent ($p < 0.05$) with those obtained using only positive correlations in both the MPI-LEMON and ABIDE-I datasets. Figure S1 displays violin plots comparing the three global measures across groups in the MPI-LEMON and ABIDE-I datasets, considering the full range of Pearson correlations from the FC matrices. The findings imply that positive correlations significantly impact overall brain functional connectivity, as the global outcomes using all correlations closely resemble those obtained solely from positive correlations. The result also highlights that positive correlations are sufficient to reflect the overall functional organization of the brain.

Additionally, we compared 1-Wasserstein, 2-Wasserstein, and bottleneck distances between intra-group and inter-group persistence diagrams for both datasets[34] and conducted a one-tailed two-sample t test to evaluate statistical differences. Figures S2 and S3 illustrate the corresponding violin plots based on positive correlations and all correlations, respectively. For all three distance measures, inter-group distances are found to be significantly higher than intra-group distances across both datasets. Table S1 reports group-wise averages and p values for persistent entropy, L^1-norm, and L^2-norm, as well as intra-group and inter-group comparisons for Wasserstein and bottleneck distances, based on both positive and all correlations from the FC matrices.

RSN-level differences in functional connectivity

RSNs are critical for understanding the intrinsic functional organization of the brain without the influence of external tasks.[10] In this study, we focused on seven well-known RSNs defined according to the Schaefer atlas, specifically visual network (29 ROIs), somatomotor network (35 ROIs), dorsal attention network (26 ROIs), salience/ventral attention network (22 ROIs), limbic

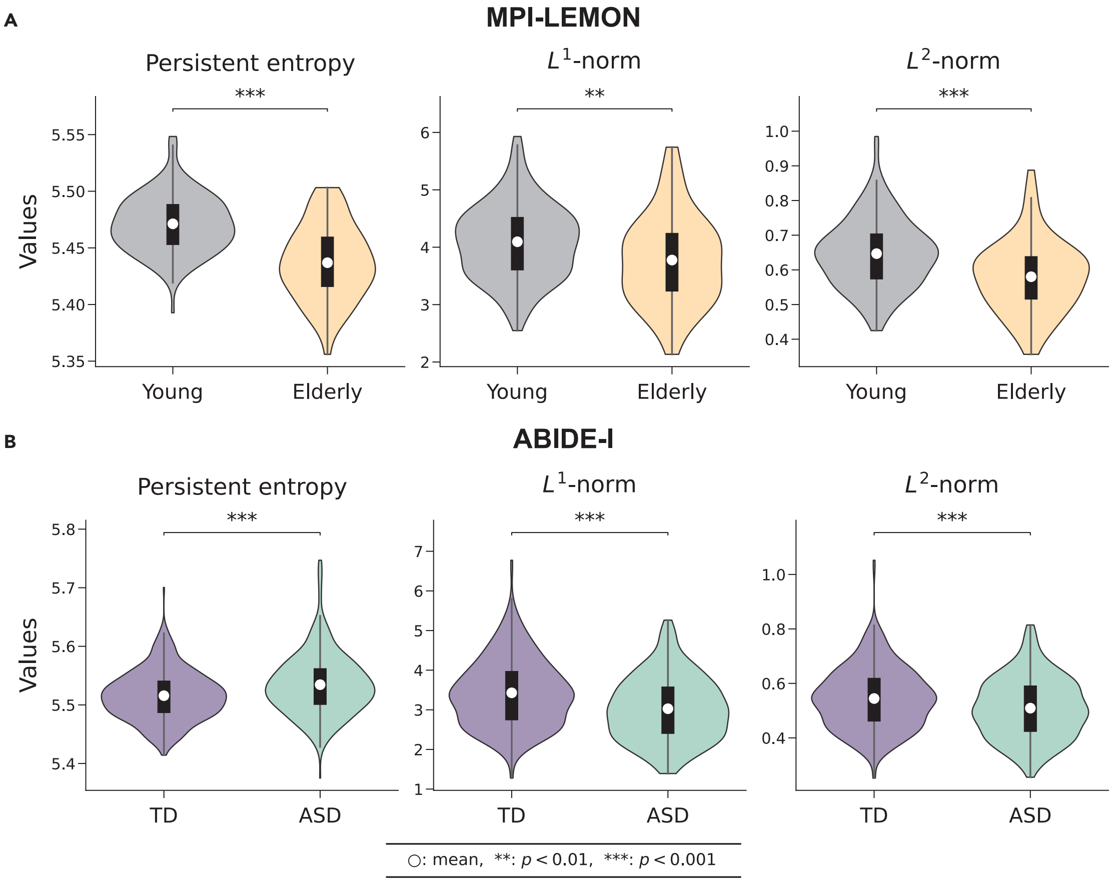

Figure 2. Brain-wide differences between the groups as identified by three global measures: Persistent entropy of the persistence barcodes and L^1-norm and L^2-norm of the persistent landscape

(A) MPI-LEMON dataset: violin plots corresponding to 153 young and 72 elderly individuals across three global measures. The mean values of all three measures are significantly higher ($p < 0.01$) in the young group compared to the elderly group.

(B) ABIDE-I dataset: violin plots corresponding to 425 typically developing (TD) individuals and 395 individuals with autism spectrum disorder (ASD) across three global measures. Average persistent entropy is significantly higher ($p < 0.001$) in the ASD group than in the TD group; however, average L^1-norm and L^2-norm are significantly lower ($p < 0.001$) in the ASD group.

network (12 ROIs), control network (30 ROIs), and default network (46 ROIs).[10,74] We examined each of the seven RSNs separately for each individual and assessed RSN-level changes in brain functional connectivity between groups by applying persistent entropy, L^1-norm, and L^2-norm. A two-tailed two-sample t test was conducted to evaluate statistical differences between the groups, and false discovery rate (FDR) correction was applied separately for each measure.

For the MPI-LEMON dataset, violin plots of persistent entropy, L^1-norm, and L^2-norm across RSNs for young and elderly groups are presented in Figure 3A. In the somatomotor, dorsal attention, salience/ventral attention, and default networks, all three measures show significant between-group differences ($p < 0.05$, FDR corrected). Only the L^1-norm and L^2-norm in the visual network, and persistent entropy in the control network, differ significantly. No differences are observed in the limbic network. Figure 3B presents results for ABIDE-I dataset, where significant differences ($p < 0.05$, FDR corrected) across all three measures are found in the somatomotor, salience/ventral attention, and default networks; no differences are observed in the other RSNs. We also performed Spearman correlation analysis between the three measures separately for each RSN. Similar to the global-scale results, the L^1-norm and L^2-norm showed a strong positive correlation ($\rho \geq 0.95$) across all four groups within each RSN. In contrast, persistent entropy exhibited moderate to high positive correlations with the L^1-norm and L^2-norm. Table S2 provides the detailed correlation coefficients and corresponding p values across all the RSNs.

Additionally, we compared 1-Wasserstein, 2-Wasserstein, and bottleneck distances between intra-group and inter-group persistence diagrams for both datasets using one-tailed two-sample t tests. Figure S4 presents violin plots comparing intra-group and inter-group distances across both datasets. Table S3 provides group-wise averages; FDR-corrected p values for persistent entropy, L^1-norm, and L^2-norm; and intra-group and inter-group comparisons of distance measures across RSNs.

ROI-level differences in the functional connectivity

In the first subsection, using whole-brain FC matrices, we showed that global topological measures based on PH can

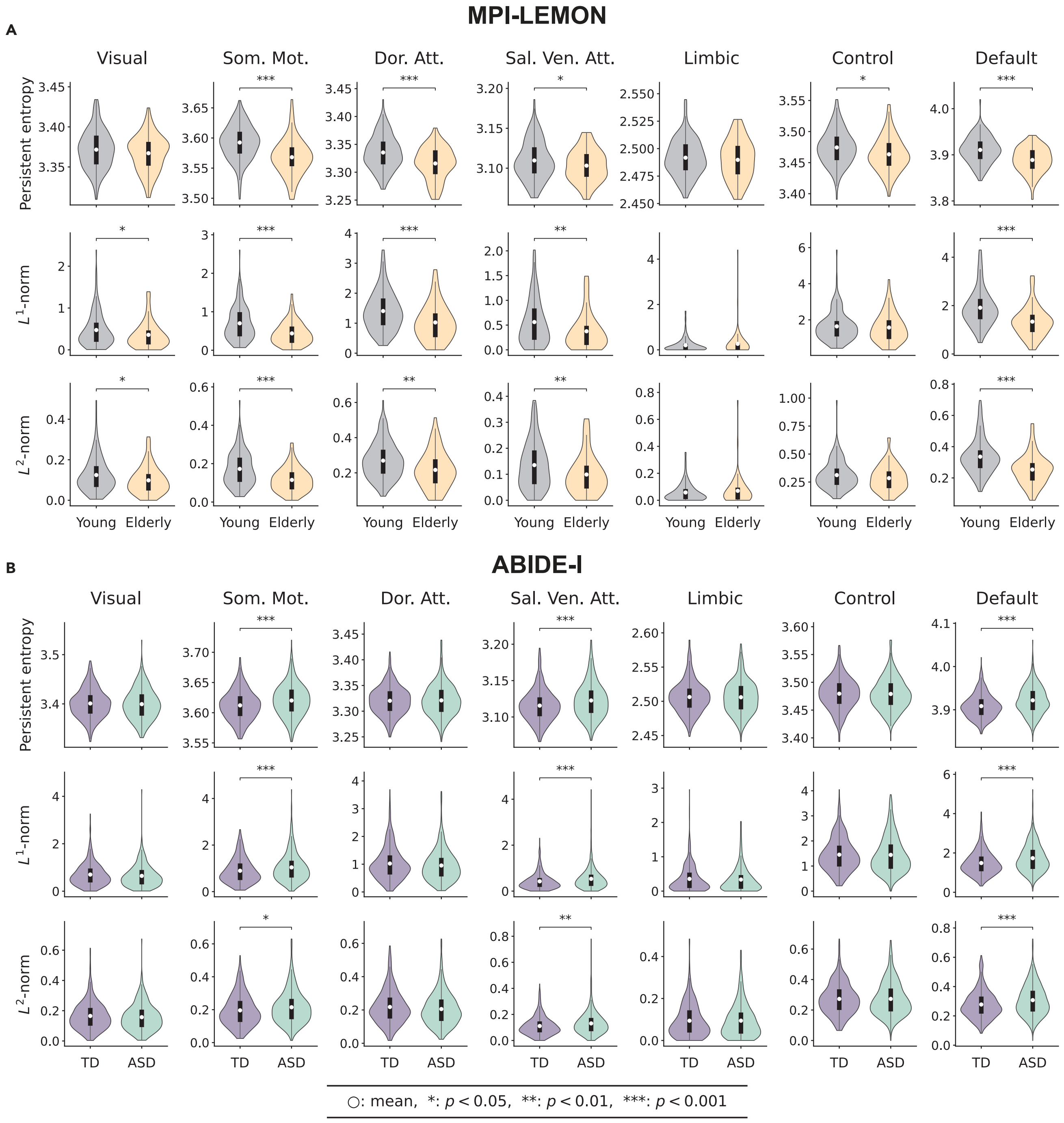

Figure 3. Resting-state-network-level differences between the groups as identified by three global measures: Persistent entropy of the persistence barcodes and L^1-norm and L^2-norm of the persistent landscape

Each row corresponds to a given measure, and each column corresponds to a given resting-state network (RSN).

(A) MPI-LEMON dataset: violin plots corresponding to 153 young and 72 elderly individuals across seven RSNs for each global measure are shown. For the somatomotor (Som. Mot.), dorsal attention (Dor. Att.), salience/ventral attention (Sal. Ven. Att.), and default networks, the mean values of all the three measures are significantly higher ($p < 0.05$, FDR corrected) in the young group compared to the elderly group. For the visual network, only the mean values of the L^1-norm and L^2-norm are significantly higher ($p < 0.05$, FDR corrected) in the young group. For the control network, only the mean of persistent entropy in the young group exhibits a significantly higher ($p < 0.05$, FDR corrected) value than the elderly group. No significant differences are observed between the groups in the limbic network for any of the three measures.

(legend continued on next page)

CellPress
OPEN ACCESS

effectively differentiate between young and elderly groups in the MPI-LEMON dataset as well as ASD and TD groups in the ABIDE-I dataset. In the second subsection, we extended this analysis to the RSN level by applying the same topological measures to RSN-specific FC matrices. This enabled us to identify the RSNs where group-level differences in functional connectivity are concentrated. In this subsection, we focus on the 200 regions, as defined by the Schaefer atlas, to investigate ROI-level differences in brain functional connectivity. To achieve this, we proposed two topology-based local measures: node persistence and node frequency (Figure 1C and methods). Node persistence quantifies the topological importance of a node by evaluating the duration of its involvement in one-dimensional holes during the filtration process. A higher node persistence value indicates that the node is consistently involved in more persistent one-dimensional holes, suggesting a key role in maintaining the loop structures of the simplicial complex. On the other hand, node frequency measures how many distinct one-dimensional holes a node belongs to, irrespective of their persistence duration throughout the filtration. We computed node persistence and node frequency across the 200 ROIs corresponding to each subject. A two-tailed two-sample t test was used to evaluate group-level differences, and FDR correction was applied to adjust for multiple comparisons.

Based on node persistence, we identified several ROIs with significant between-group differences in both datasets. In the MPI-LEMON dataset, 108 ROIs exhibit significant differences ($p < 0.05$, FDR corrected) between the young and elderly groups. Among the seven RSNs, the ROIs are categorized as visual (12), somatomotor (22), dorsal attention (17), salience/ventral attention (11), limbic (2), control (15), and default (29) networks. All of these ROIs, except for one in the limbic network (RH_Limbic_TempPole_1), show higher node persistence values in young individuals compared to elderly individuals. In the ABIDE-I dataset, 27 ROIs exhibit significant differences ($p < 0.05$, FDR corrected) between the ASD and TD groups. The ROIs are distributed as visual (2), somatomotor (9), dorsal attention (1), salience/ventral attention (1), limbic (3), control (4), and default (7) networks. All the ROIs show higher node persistence values for individuals with ASD. Figure 4 depicts the ROIs with statistically significant between-group differences ($p < 0.05$, FDR corrected) based on node persistence for both datasets.

In contrast, node frequency reveals 39 and 35 ROIs with significant between-group differences ($p < 0.05$, FDR corrected) for the MPI-LEMON and ABIDE-I datasets, respectively (Figure S5). In the MPI-LEMON dataset, 39 ROIs are distributed as follows: visual (3), somatomotor (6), dorsal attention (9), salience/ventral attention (4), limbic (2), control (3), and default (12) networks. Moreover, the ROIs identified through node frequency are a subset of those identified by node persistence. In the ABIDE-I dataset, 35 ROIs are distributed in six RSNs as follows: visual (5), somatomotor (11), dorsal attention (2), salience/ventral attention (5), control (5), and default (7) networks. Moreover, 21 out of these 35 ROIs overlap with those found via node persistence. Table S4 lists ROIs with significant between-group differences identified by both node persistence and node frequency. For each of the 200 ROIs, group-wise averages of node persistence and node frequency are provided in Table S5, along with FDR-corrected p values, for both the MPI-LEMON and ABIDE-I datasets.

Behavioral and cognitive relevance of ROI-level differences

In the preceding subsection, we found that node persistence can identify 108 ROIs with significant differences between young and elderly individuals in the MPI-LEMON dataset and 27 ROIs with significant differences between ASD and TD groups within the ABIDE-I dataset. These ROIs are distributed across multiple RSNs defined by the Schaefer atlas. Subsequently, to identify the cognitive domains related to these significant ROIs, we performed a Neurosynth meta-analysis (see methods) for each RSN. Only the ROIs exhibiting significant between-group differences in node persistence were considered in this analysis.

In the MPI-LEMON dataset, our analysis primarily focused on somatomotor, dorsal attention, salience/ventral attention, and default networks, as these RSNs not only exhibit significant differences across three global measures (persistent entropy, L^1-norm, and L^2-norm) but also contain the majority of ROIs with significant differences in node persistence. For the ABIDE-I dataset, we focused on the somatomotor and default networks, as they exhibit significant group differences at the RSN level and also contain the majority of ROIs with significant differences identified via node persistence. Although the salience/ventral attention network also showed significant group differences at the RSN level in ABIDE-I, it is excluded here, as only one region is identified using node persistence.

Figures 5A and 5B show word clouds illustrating the behavioral relevance of key brain regions in four RSNs for the MPI-LEMON dataset and two RSNs for the ABIDE-I dataset, respectively. We found that ROIs with age-related changes in node persistence are associated with movement in the somatomotor and dorsal attention networks; somatosensory and affective processing in the salience/ventral attention network; and language, social cognition, and memory in the default network. We found that ROIs with ASD-related differences in node persistence are associated with movement in the somatomotor network and social cognition in the default network. The terms linked to significantly different ROIs across all seven RSNs for both datasets are summarized in Table S6.

Correlation of topological measures with phenotypic and clinical scores

We conducted a correlation analysis to examine the relationship of PH-based metrics with phenotypic test scores for the MPI-LEMON dataset and clinical scores of symptom severity for individuals with ASD in the ABIDE-I dataset. In the MPI-LEMON dataset, significant positive correlations were found between PH-based measures and Trierer Inventar zum

(B) ABIDE-I dataset: violin plots corresponding to 425 typically developing (TD) individuals and 395 individuals with autism spectrum disorder (ASD) across seven RSNs for each global measure are shown. For the somatomotor (Som. Mot.), salience/ventral attention (Sal. Ven. Att.), and default networks, the mean values of all three measures are significantly lower ($p < 0.05$, FDR corrected) in the ASD group compared to the TD group. For visual, dorsal attention (Dor. Att.), limbic, and control networks, none of the three measures exhibit significant differences between the TD and ASD groups.

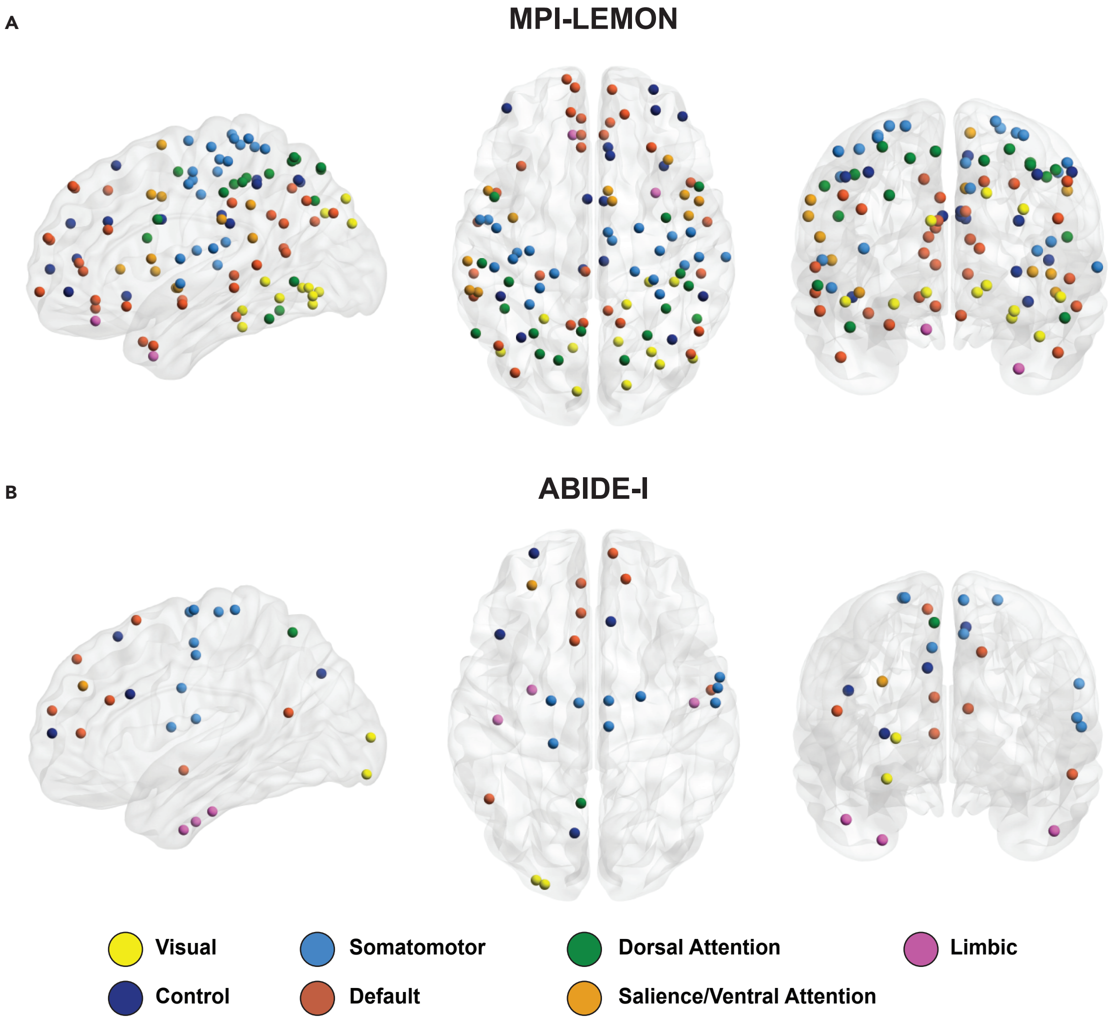

Figure 4. Visual representation of brain regions with significant between-group differences in node persistence

(A) MPI-LEMON dataset: 108 regions with significant differences ($p < 0.05$, FDR corrected) in node persistence between the healthy young and healthy elderly groups. For every region, young individuals show higher node persistence compared to the elderly individuals, except for one region in the limbic network (RH_Limbic_TempPole_1).

(B) ABIDE-I dataset: 27 regions with significant differences ($p < 0.05$, FDR corrected) in node persistence between the autism spectrum disorder (ASD) and typically developing (TD) groups. All regions reveal higher node persistence for individuals with ASD relative to TD individuals. Each brain region is assigned to one of the seven resting-state networks (RSNs) as defined by the Schaefer atlas. The regions are colored according to their respective RSNs, as shown at the bottom. The visualization was generated using BrainNet Viewer.[80] Table S4 lists the significantly different regions of interest identified via node persistence across both datasets.

Chronischen Stress (TICS) scores at both the brain-wide and RSN levels, particularly within the visual, dorsal attention, and salience/ventral attention networks (Table S7). These correlations suggest that higher PH-based measures are linked to higher levels of chronic stress. At the ROI level, correlations indicated that age-related differences in node persistence within only two brain regions were related to chronic stress (Table S8). In the ABIDE-I dataset, no significant correlations were identified between topology-based measures and clinical scores of ASD symptom severity at the brain-wide, RSN, or ROI levels (Tables S8 and S9). Our findings suggest that PH-based measures capture latent or compensatory mechanisms that reliably distinguish ASD from TD.[81,82] However, symptom severity in ASD likely reflects more heterogeneous influences,[83] including genetic, metabolic,[84,85] and developmental factors,[86,87] which may not be directly represented in functional connectivity. This could be a reason why PH-based measures fail to show strong correlations with symptom severity. A detailed description of the correlation analysis is provided in Note S1.

Linking ROI-level differences in topological measures with NIBS outcomes

Alongside the meta-analysis decoding, we conducted an additional analysis to evaluate the relevance of our findings with the existing literature on NIBS in healthy elderly individuals and individuals with ASD. We focused on three commonly used NIBS techniques, namely transcranial direct current stimulation (tDCS), transcranial alternating current stimulation (tACS), and

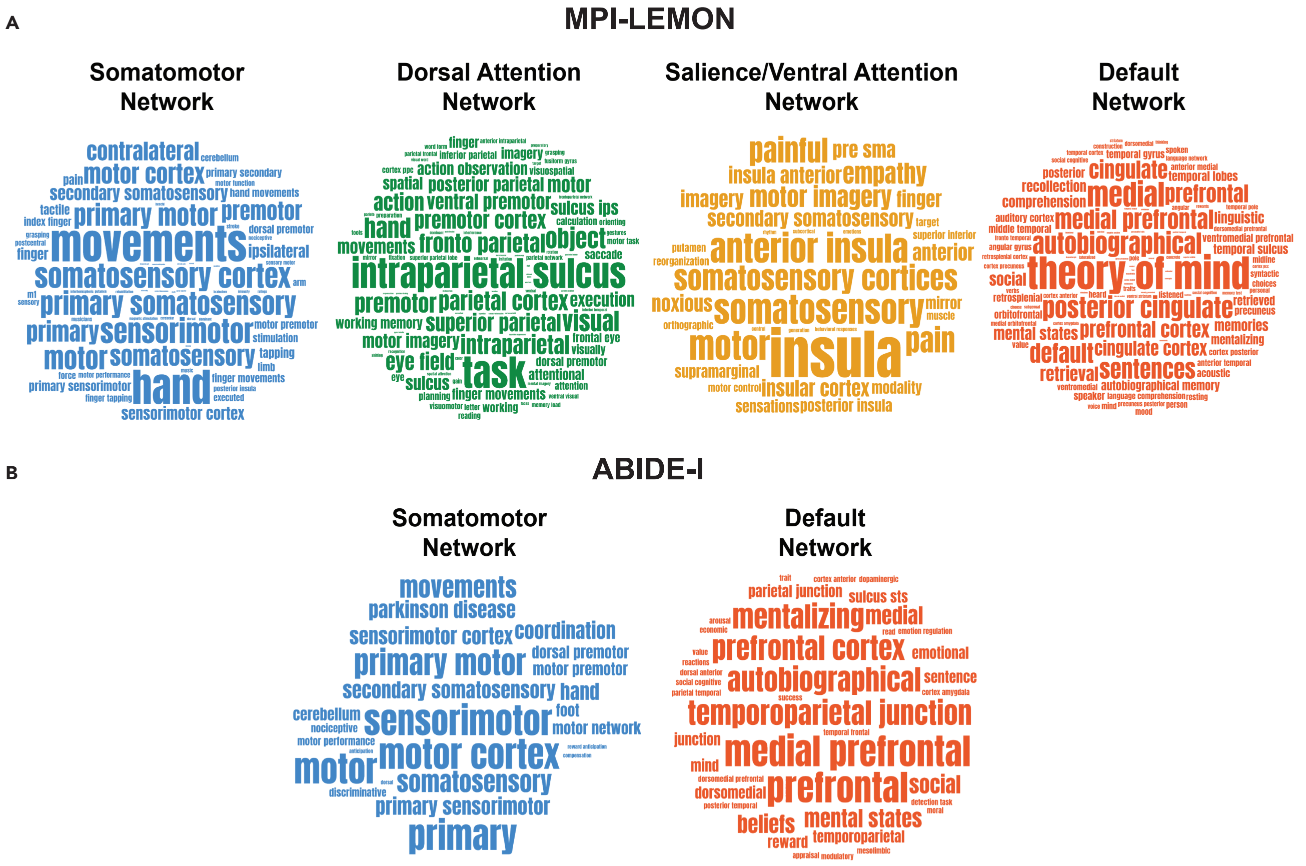

Figure 5. Behavioral and cognitive relevance of ROI-level changes using Neurosynth meta-analysis decoding

(A) MPI-LEMON dataset: word clouds highlighting cognitive and behavioral terms related to significantly different age-related brain regions identified via node persistence in four RSNs: somatomotor, dorsal attention, salience/ventral attention, and default networks.

(B) ABIDE-I dataset: word clouds highlighting cognitive and behavioral terms related to significantly different brain regions ($p < 0.05$, FDR corrected) between individuals with autism spectrum disorder (ASD) and typically developing (TD) individuals, as identified via node persistence, in two RSNs: somatomotor and default networks. The words are scaled according to their frequency of occurrence. However, the scaling is performed independently for each word cloud. In other words, frequencies are not visually comparable across different word clouds. The word clouds are created using the online tool https://www.wordclouds.com.

transcranial magnetic stimulation (TMS). For the MPI-LEMON dataset, we investigated the overlap between brain regions exhibiting differences in PH-based measures and those where NIBS has been shown to enhance motor performance in healthy elderly individuals. For the ABIDE-I dataset, we investigated the overlap with brain regions where NIBS has indicated reductions in ASD-related symptoms. The sets of brain regions where NIBS interventions have shown beneficial outcomes were curated in two prior studies conducted by some of us.[69,71]

The data derived from previous NIBS studies in healthy elderly individuals revealed four cortical target regions with evidence for improvement in motor function, namely primary motor cortex, dorsolateral prefrontal cortex, posterior parietal cortex, and right supplementary motor area. These target regions are mapped to 42 ROIs in the Schaefer atlas across five RSNs: somatomotor (11), dorsal attention (12), salience/ventral attention (4), control (8), and default (7) networks. The Euler diagram in Figure 6A illustrates the overlaps among 42 ROIs exhibiting improvements in motor function following NIBS, along with the ROIs identified by node persistence and node frequency. Additionally, it highlights both the overlaps and unique ROIs identified across NIBS studies, node persistence, and node frequency. Among these 42 clinically relevant ROIs, 27 are detected using node persistence and ten are detected using node frequency. The clinically relevant ROIs detected by node persistence and node frequency are distributed across five and four RSNs, respectively. No clinically significant ROIs are found within the visual and limbic networks. Further, the ten clinically relevant ROIs detected by node frequency are a subset of those detected by node persistence (Figure 6A and Table S10). In other words, node persistence uniquely identified 17 clinically relevant ROIs.

The data derived from previous NIBS studies in individuals with ASD revealed five cortical target regions that show evidence for improving behavioral or cognitive symptoms associated with ASD, namely premotor cortex, dorsolateral prefrontal cortex, pars triangularis, pars opercularis, and left primary motor cortex. These target regions encompass 31 Schaefer ROIs and are distributed across five RSNs: somatomotor (10), dorsal attention (5), salience/ventral attention (4), control (5), and default (7) networks. The Euler diagram in Figure 6B shows the overlaps

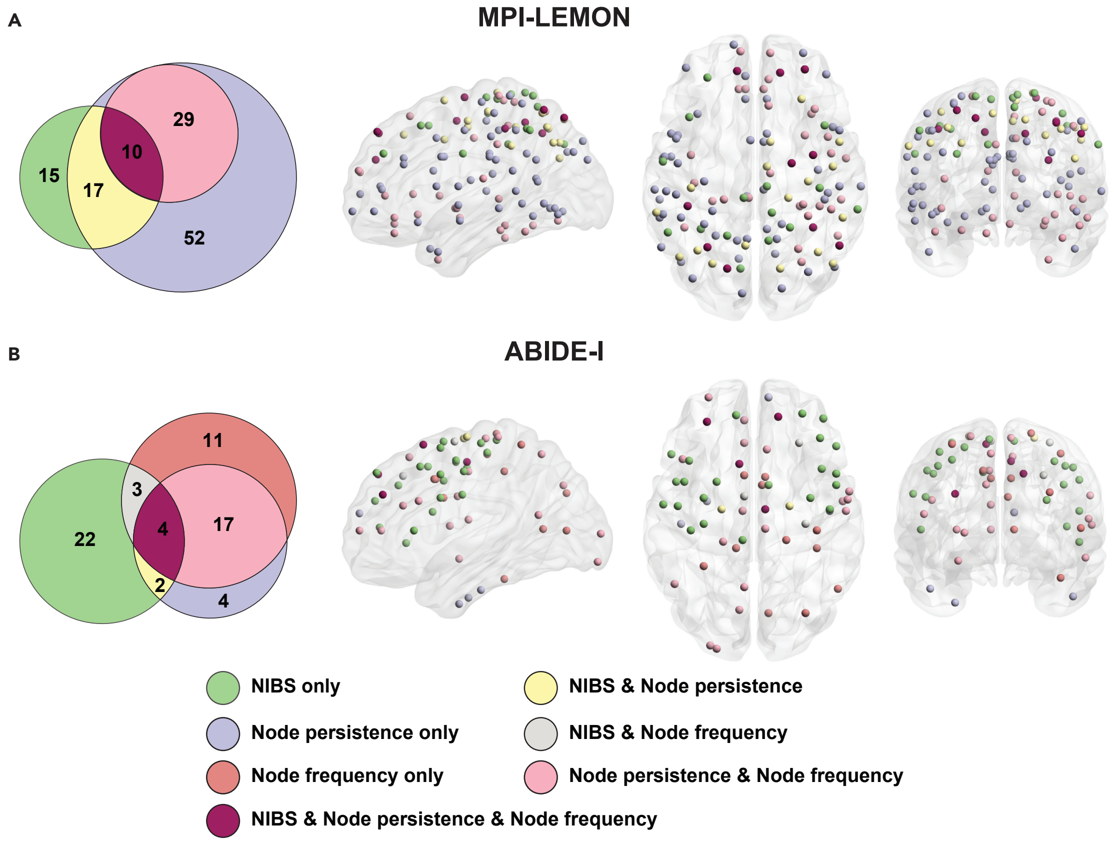

Figure 6. Visual representation of brain regions identified via non-invasive brain stimulation and local persistent-homology-based measures

(A) MPI-LEMON dataset: Euler diagram depicting overlaps between brain regions identified by non-invasive brain stimulation (NIBS) (42), node persistence (108), and node frequency (39), along with unique regions in each set. Accompanying this, the brain regions corresponding to each partition of the Euler diagram are visually represented. Within NIBS, 27 of 42 ROIs are identified considering node persistence, node frequency, or both. Seventeen ROIs are uniquely identified using node persistence. Ten ROIs identified via node frequency form a subset of ROIs detected by node persistence.

(B) ABIDE-I dataset: Euler diagram depicting overlaps between brain regions identified by NIBS (31), node persistence (27), and node frequency (35), along with unique regions in each set. Accompanying this, the brain regions corresponding to each partition of the Euler diagram are visually represented. Within the 31 ROIs in NIBS, nine ROIs are identified considering either node persistence, node frequency, or both. Two and three ROIs are uniquely identified considering node persistence and node frequency, respectively.

among the 31 ROIs exhibiting ASD-related improvements following NIBS and ROIs identified by PH-based local measures. It also illustrates both the overlaps and unique ROIs identified across NIBS studies, node persistence, and node frequency. Among these 31 clinically relevant ROIs, six are detected using node persistence and seven are detected considering node frequency. These clinically relevant ROIs are distributed across three RSNs: somatomotor, salience/ventral attention, and default networks. Both the local measures collectively identified 9 out of 31 clinically relevant ROIs, with two uniquely identified by node persistence and three uniquely detected by node frequency (Figure 6B and Table S10).

Comparison with existing local measures based on PH

Petri et al.[51] introduced the concept of homological scaffolds, which are secondary networks that summarize one-dimensional holes (cycles) captured by PH. Building on this idea, nodal persistence scaffold strength (nodal PSS) was proposed as a node-level centrality measure that quantifies the contribution of a region to these cycles.[52] Specifically, nodal PSS considers the edges involved in representative H_1 cycles extracted from PH via JavaPlex[88] and assigns strength scores to nodes accordingly. Other local topological measures, such as local PH, can also be defined at the level of individual nodes in a simplicial complex.[66] In this study, we included nodal PSS as a reference method to compare with our proposed local topological measures of node persistence and node frequency, since all these measures characterize the structure of one-dimensional holes. Further, nodal PSS has also been employed in the context of fMRI data analysis.[52]

Notably, both node persistence and node frequency are less computationally expensive than the construction of homological scaffolds. The latter is significantly more time consuming, particularly during the filtration process, where building the simplicial complex becomes increasingly intensive for larger point sets. This makes node persistence and node frequency better suited for node-level PH analyses on larger simplicial complexes compared to nodal PSS. In particular, when applied to FC matrices with 200 regions, we found that the scalability of homological scaffold construction significantly diminishes. Therefore,

to identify ROI-level changes in functional connectivity using nodal PSS, we computed nodal PSS by considering FC matrices at the level of RSNs (RSNs to local). Specifically, the homological scaffolds were constructed independently for each RSN, and nodal PSS was computed for each ROI within these networks. To ensure a consistent comparison with our proposed measures, we also recomputed node persistence and node frequency using FC matrices restricted to individual RSNs. In other words, our comparative analysis focused solely on intra-RSN topological patterns by excluding inter-RSN connections, thereby enabling the detection of ROI-level differences within each RSN. A two-tailed two-sample *t* test was utilized to detect statistical differences between the groups, and FDR correction was applied independently for each RSN.

In the MPI-LEMON dataset, node persistence, node frequency, and nodal PSS identify age-related differences in 59, 40, and 58 ROIs, respectively ($p < 0.05$, FDR corrected). In the ABIDE-I dataset, these three measures reveal significant ASD-related differences in 53, 35, and 45 ROIs, respectively ($p < 0.05$, FDR corrected). The overlaps among these sets and their distribution across RSNs are provided in Note S2. All significant ROIs identified using node persistence, node frequency, and nodal PSS for both datasets are listed in Table S11. Additionally, Table S12 provides the group-wise averages of node persistence, node frequency, and nodal PSS for each of the 200 ROIs across both datasets, along with the corresponding FDR-corrected *p* values.

Finally, we investigated the overlap between our RSN-to-local-level analysis results and clinically validated ROIs from the NIBS literature. The UpSet plots[89] in Figure S6 illustrate the number of ROIs associated with clinical improvement and how many of these were identified using node persistence, node frequency, and nodal PSS, along with their intersections, for both MPI-LEMON and ABIDE-I datasets. In the MPI-LEMON dataset, of the 42 clinically relevant regions identified through NIBS, node persistence, node frequency, and nodal PSS detected 15, 13, and 18 ROIs, respectively. Similarly, in the ABIDE-I dataset, out of the 31 clinically relevant ROIs identified by NIBS, the three measures identified nine, three, and seven ROIs, respectively. Further details on the overlap between regions identified by local PH measures and NIBS-targeted regions are provided in Note S3 and Table S10.

To summarize, when examining ROI-level differences in functional connectivity during healthy aging and ASD using RSN-level FC matrices, node persistence identifies more brain regions than nodal PSS. In determining clinically relevant regions, nodal PSS detects more regions than node persistence in healthy aging, whereas in autism, node persistence identifies more regions than nodal PSS. However, both node persistence and nodal PSS can uniquely identify certain clinically relevant ROIs across both datasets.

Robustness of PH-based local measures

The two PH-based local measures proposed in this study are defined with respect to the representative cycle of each one-dimensional hole. Since PH does not guarantee unique representative cycles, the particular choice of cycle may influence the resulting node-level measures. Therefore, it is essential to evaluate the robustness of the proposed measures under different representative cycle selections. In JavaPlex, the representative cycle obtained from a given distance matrix is deterministic for a fixed node order. However, altering the node order can lead to different representative cycles for the same one-dimensional hole. Therefore, to address the robustness of the ROI-based measures, we generated 20 different permutations of node orderings and rearranged the FC matrix accordingly. Next, we computed node persistence and node frequency for all of the subjects from their permuted FC matrices. This procedure provides alternative representations of one-dimensional holes, allowing us to assess the robustness of the proposed measures. Note that this analysis is performed only for the MPI-LEMON dataset.

Since the distributions of node persistence and node frequency were found to be skewed, we adopted robust variability indices: median absolute deviation (MAD) and its normalization with respect to the median (MAD/M).[90,91] This index provides a scale-independent measure of dispersion that is less affected by skewness or outliers. Small values of MAD and MAD/M indicate that the measures remain stable across different cycle choices, whereas larger values reflect greater sensitivity of the node-level measures to the specific representative cycles. These indices allowed us to quantify the robustness of the proposed measures under different representative cycle choices. The variability index was calculated separately for each subject and each node. For each node, Table S13 reports the average, first quartile (Q1), median (Q2), and third quartile (Q3) of MAD and MAD/M for the node measures, summarized across individuals in young and elderly groups of the MPI-LEMON dataset.

First, we examined the extent to which node persistence and node frequency values vary across individual FC matrices when different representative cycles are considered. Our results indicate that, for both measures, some sensitivity is present but remains relatively small in magnitude (Note S4). For instance, in the case of node frequency, most nodes exhibited an average MAD value below 1. Given that node frequency can only take integer values, this finding suggests that, although the choice of representative cycles may vary, the number of cycles to which a node belongs remains fairly stable. Overall, variability in node-level measures due to different representative cycle choices was minimal for most nodes in both the young and elderly groups, supporting the robustness of the proposed measures. The summary statistics of both measures across both young and elderly groups are presented in Table S13.

Second, we examined whether the ranking of the nodes based on node persistence and node frequency remains consistent across different cycle choices. To evaluate this, we computed Spearman correlations between all pairs of the 20 node permutations for each subject, separately for both measures. The correlations were then averaged across individuals within each group, yielding four 20×20 correlation matrices corresponding to the two measures in the young and elderly groups. We observed that, across all comparisons, the Spearman correlations were consistently high ($\rho \geq 0.8$) for both measures. This indicates that the node rankings remain largely unchanged across different choices of representative cycles of one-dimensional holes. Figure S7 presents the pairwise correlations across 20 random permutations for node persistence and node frequency in both the young and elderly groups.

Third, we examined whether the group-level differences obtained by node persistence and node frequency depend on the cycle choice. To address this and assess the consistency of our findings, we performed a two-tailed two-sample t test to identify significantly different regions or nodes between the young and elderly groups across each of the 20 node permutations. Using node persistence, the number of significantly different regions across permutations ranged from 99 to 116, with an average of 107. In our analysis, we followed the canonical node order provided by Schaefer et al.[74] and identified 108 regions in total. Of these, on average, 91 regions overlapped with those obtained across different node permutations. Similar results were observed for node frequency (Note S5). Across 20 randomizations, the number of significantly different regions ranged from 38 to 53, with an average of 45. Additionally, we evaluated the extent to which regions identified across different node permutations coincided with NIBS-identified regions. In this case as well, the number of overlapping regions remained within a small range. Table S14 summarizes the results for the PH-based local measures across 20 permutations of node orderings.

Finally, we extended these analyses to the RSN-to-local level, considering node persistence, node frequency, and nodal PSS. The main findings were consistent across all measures. Variability indices showed only minimal fluctuations across nodes, indicating the stability of these metrics at the RSN-to-local level (Note S4 and Table S15). Moreover, both the number of significantly different nodes and their overlap with NIBS-identified regions remained within a narrow range (Note S5 and Table S14). In summary, although the PH-based local measures differ slightly at some nodes depending on the different choices of representative cycles, the overall number of significantly different regions between the groups remains largely within a short interval.

DISCUSSION

This study attempts to apply PH, a key tool in TDA, to analyze brain functional connectivity in individuals undergoing healthy aging and those with ASD at three different scales: (1) global (brain-wide changes), (2) mesoscopic (RSN-level changes), and (3) local (ROI-level changes). In addition, we introduce a scalable and computationally efficient method for extracting local topological insights from brain functional connectivity using PH.

In this study, we acquired FC matrices derived from the MPI-LEMON and ABIDE-I datasets to investigate changes in brain functional connectivity associated with healthy aging and autism, respectively. The FC matrix, representing pairwise Pearson correlations among 200 brain regions as specified by the Schaefer atlas, served as the initial input for our PH-based analysis. We focused on functionally meaningful interactions by retaining only positive correlations, which were subsequently transformed into ultrametric distance matrices. This transformation allowed us to compute PH-based measures via Rips filtration. In particular, we employed persistent entropy of the persistence barcodes, L^1-norm and L^2-norm of the persistence landscape, for both brain-wide and RSN-level investigations. For the ROI-level analysis, we employed our proposed metrics, node persistence and node frequency, to identify specific ROIs responsible for functional connectivity differences.

At the global scale, our analysis demonstrated significant differences in topological measures between the groups. A higher persistent entropy indicates a more uniform distribution of the persistence of features across the filtration, suggesting the absence of dominant topological structures. Conversely, a lower persistent entropy reflects a concentration of longer-lived features, implying the presence of prominent topological structures within the data.[77] Higher L^1-norm and L^2-norm of the persistence landscape indicate that one-dimensional topological features persist longer, capturing their dominance and longevity across the filtration. Additionally, 1-Wasserstein, 2-Wasserstein, and bottleneck distances revealed higher inter-group distances compared to intra-group distances for both the MPI-LEMON and ABIDE-I datasets, indicating a greater topological similarity within groups than between groups.

Mesoscale analysis revealed that, although significant topological differences were observed at the global level, they do not arise uniformly across all RSNs. In other words, only specific RSNs contribute to the observed effects. At the local scale, significantly different nodes are mostly concentrated within the RSNs that also exhibited significant group differences during our mesoscale analysis. This highlights the consistency of our findings across different scales. Notably, the healthy aging study indicates a strong agreement between the two measures. In contrast, the ASD study indicates that the two measures may capture distinct aspects of region-level topological differences. Remarkably, our methodology enabled the extraction of multiscale topological features from FC matrices, revealing brain regions responsible for alterations in resting-state functional connectivity associated with healthy aging and ASD.

Next, we used Neurosynth meta-analysis decoding to determine the behavioral and cognitive relevance of the changes arising due to healthy aging and ASD, as identified by node persistence. Our analysis revealed that the brain regions exhibiting age-related differences in node persistence are mainly related to movement, language, social cognition, memory, somatosensory, and affective processing. Previous research suggests that aging impacts various domains of motor performance,[92,93] including coordination,[94] movement variability,[95] and speed,[96] as well as language production[97] and affective processing.[98] It also leads to diminished somatosensory functions such as sensitivity to warmth, touch, and vibration,[99] collectively highlighting the physical and emotional changes associated with aging.[98] Our analysis revealed that the brain regions exhibiting ASD-related differences in node persistence are mainly related to movement and social cognition. Previous meta-analysis decoding suggests that ASD influences the cognitive domains.[17–19,100–106] Hence, our findings indicate that regions exhibiting differences in node persistence in both healthy aging and ASD are associated with cognitive domains and abilities that are commonly reported to undergo age-related or ASD-related changes.

Following the meta-analysis, we assessed the alignment between our findings and prior tDCS, tACS, and TMS studies. Our analysis revealed that brain regions exhibiting altered PH-based local measures in both healthy elderly individuals and those with ASD overlap with regions previously shown to benefit

from non-invasive stimulation, either through improved motor performance in aging or reduction in symptom severity in ASD. Furthermore, in determining clinically significant brain regions, node persistence proved superior in detecting a larger number of clinically relevant brain regions in the context of healthy aging, while for autism, node persistence and node frequency revealed a comparable number of clinically relevant brain regions. Notably, this study also makes an attempt to validate PH-based analyses of brain functional connectivity networks using evidence from non-invasive brain stimulation (tDCS/tACS/TMS) studies.

In aging, postural adaptability typically declines, reflected in reduced center of pressure (COP) complexity due to weakened integration of visual, vestibular, and proprioceptive systems. Prior research[107,108] has shown that tDCS over the left prefrontal and primary motor cortices enhances COP complexity and improves balance, indicating that stimulation of these regions can acutely boost adaptability under cognitive load. Similarly, in ASD, tDCS/TMS applied over the left and right prefrontal motor areas and dorsolateral prefrontal cortex has improved Autism Treatment Evaluation Checklist (ATEC) scores, particularly in social relating behaviors and overall functioning.[109,110] Consistent with these findings, our node persistence analysis identified regions associated with aging (e.g., 7Networks_LH_Default_PFC_9, 7Networks_LH_SomMot_6, and 7Networks_LH_SomMot_10) and ASD (e.g., 7Networks_LH_SomMot_14 and 7Networks_LH_SalVentAttn_PFCl_1), which are part of the prefrontal and primary motor cortices and overlap with previously reported tDCS or TMS targets. This convergence underscores the potential of node-persistence-identified regions as biomarkers of functional adaptability, while also highlighting the broader utility of PH-based measures in generating hypotheses for clinically relevant stimulation targets, which can subsequently be evaluated through non-invasive stimulation protocols. Although further validation in larger and clinically diverse cohorts is required, these findings suggest a possible bridge between topological analyses and translational neuroscience applications.

We compared our proposed local topological measures with nodal PSS, an existing method that also characterizes one-dimensional holes in functional connectivity networks. Node persistence was found to capture more brain regions with significant group differences compared to nodal PSS in both the MPI-LEMON and ABIDE-I datasets. However, when aligning our findings with non-invasive stimulation outcomes, we found that the regions highlighted by node persistence do not entirely overlap with those identified by nodal PSS. This suggests that different local topological measures may offer complementary insights into clinically relevant brain regions. Due to the higher computational complexity of nodal PSS, these comparisons were conducted using FC matrices restricted to individual RSNs. In contrast, our proposed measures, node persistence and node frequency, are scalable and were applied to FC matrices involving all ROIs in the main analyses reported in this study. This scalability and methodological flexibility make our approach well suited for neuroimaging studies involving large populations.

Finally, as the ROI-based measures are directly related to the representative cycles of one-dimensional holes and such cycles are not unique, we conducted a robustness analysis to evaluate their sensitivity to different cycle choices. Our findings indicate that ROIs exhibit varying degrees of robustness: while some nodes remain highly stable irrespective of cycle selection, some are more sensitive to these choices. Nevertheless, the majority of nodes demonstrate moderate to high robustness. Importantly, although the values for most ROIs differ slightly across different cycles, their relative rankings remain largely preserved. Moreover, when comparing between groups, the majority of ROIs that displayed significant differences remained consistent across different representative cycle choices, highlighting the robustness of the group-level findings.

Although PH-based measures provide rigorous characterizations of higher-order brain network structure, their neurobiological interpretation remains abstract. For instance, a one-dimensional loop may capture functionally related yet indirectly connected regions, although its precise mechanistic relationship to brain physiology remains to be fully elucidated. Thus, PH features are best regarded as descriptive markers of network organization rather than direct mechanistic indicators.[61,111] One a priori mechanistic framework that links brain network organization to behavior and cognition is provided by the functional segregation and integration hypothesis.[112] Functional segregation refers to the specialized processing of information within locally interconnected groups shaped by development and activity-dependent selection. On the other hand, integration refers to coordinated communication across distinct brain regions via a short chain of synaptic pathways. Current evidence indicates that both properties coexist in brain connectivity networks. In other words, information processing in the brain is both localized and distributed, and deviation in either property could lead to atypical behavior or cognitive and motor impairment. PH is fundamentally a global measure, as it tracks topological features such as connected components and cycles across multiple scales. It can therefore be considered to capture the functional integration of brain connectivity. Recent studies have highlighted this perspective,[61] and future work could explore it more systematically.

In conclusion, we found that PH facilitates the identification of age-related as well as ASD-related changes in functional connectivity at multiple spatial scales. Further, we introduced two local PH-based measures, namely node persistence and node frequency, to detect topology-influenced variations at the level of individual brain regions. Moreover, to support the clinical relevance of our findings for non-invasive brain stimulation, we demonstrated that local measures based on PH can effectively identify brain regions impacted by healthy aging and ASD. One limitation of our approach is that the node-level measures currently consider only one-dimensional topological features such as loops or cycles, potentially overlooking more complex structures. Future research could extend this framework to incorporate higher-dimensional features, such as voids, for a more comprehensive characterization of network topology. Furthermore, node persistence could be utilized to examine local characteristics of brain functional connectivity networks beyond aging and ASD and could potentially be extended to other types of networks, such as task-based fMRI networks. While our analyses focused on assessing group-level differences, validation of the findings, and robustness of

PH-based nodal measures, an important next step should be to evaluate their predictive utility. In particular, future work can focus on examining whether node persistence and node frequency can improve out-of-sample prediction of age or clinical status compared with both existing PH-based metrics and standard graph-theoretical measures.

METHODS

In this study, we utilize PH-based metrics to investigate changes in resting-state functional connectivity in individuals undergoing healthy aging and those with ASD. First, we acquired FC matrices obtained from rs-fMRI images of subjects from the MPI-LEMON[68] and ABIDE-I datasets.[70] Second, we constructed a sequence of nested Rips complexes[113] on the distance matrix derived from each FC matrix. Third, we computed topological invariants based on PH to identify the changes in functional connectivity at three different scales: (1) global scale (brain-wide changes), (2) mesoscopic scale (RSN-level changes), and (3) local scale (ROI-level changes).

FC matrices

The FC matrices used in this study are obtained from preprocessed rs-fMRI images of subjects from the MPI-LEMON and ABIDE-I datasets. These FC matrices were previously generated by some of us using the CONN functional connectivity toolbox[114] and are available at https://github.com/asamallab/Curvature-FCN-Aging and https://github.com/asamallab/Curvature-FCN-ASD. The FC matrix of each subject is a 200×200 square matrix representing the pairwise correlations among the 200 ROIs as outlined in the Schaefer atlas.[74] Each matrix entry (i,j) contains a numerical value representing the Pearson correlation between the time series of ROI i and ROI j. The time series for each ROI was computed by averaging the time series of all voxels located within that region from the preprocessed fMRI images. For a comprehensive overview of the preprocessing pipeline and FC matrix generation, readers are referred to Yadav et al.[69,71] and Elumalai et al.[71]

In addition to assigning each voxel to one of 200 ROIs, the Schaefer atlas[74] also associates each ROI with one of seven RSNs. These RSNs include the visual network (29 ROIs), somatomotor network (35 ROIs), dorsal attention network (26 ROIs), salience/ventral attention network (22 ROIs), limbic network (12 ROIs), control network (30 ROIs), and default network (46 ROIs). This parcellation method allows for examining brain functional connectivity at the local scale (ROI level) as well as at the mesoscopic scale (RSN level).

A summary of the datasets obtained for this study is as follows.

(1) MPI-LEMON: the FC matrices of 225 subjects from the Max Planck Institute Leipzig Study for Mind-Body-Emotion Interactions (MPI-LEMON) dataset[68] consists of 153 healthy young (age range 20–35 years) and 72 healthy elderly individuals (age range: 59–77 years).[69] Notably, the MPI-LEMON dataset does not include any middle-aged individuals.
(2) ABIDE-I: the FC matrices of 820 subjects (age range: 7–64 years) from the ABIDE-I project[70] consists of 395 individuals with ASD and 425 age-matched TD individuals.[71]

Construction of Rips complex from FC matrix and PH

Given an FC matrix ($\boldsymbol{C}$), the ultrametric distance matrix[75] $\boldsymbol{D}$ is constructed using the distance measure given by

$$\boldsymbol{D}_{ij} = \sqrt{2(1 - \boldsymbol{C}_{ij})}, \quad \text{(Equation 1)}$$

where $\boldsymbol{C}_{ij}$ is the Pearson correlation between the brain regions i and j. The ultrametric distance in our analyses is defined based solely on positive correlations in the FC matrix, reflecting the dominating role of positive connectivity in supporting overall brain function.[76,115] As a result, each value in the distance matrix ranges between 0 and $\sqrt{2}$. These distance matrices are utilized to construct the Rips complex[113] and to compute PH on the filtration of Rips complexes.

A Rips complex is a type of simplicial complex constructed from a set of points S in a metric space. A simplicial complex K is a higher-dimensional generalization of a graph, constructed from simplices such as vertices, edges, triangles, tetrahedra, and their higher-dimensional counterparts. For a given radius ϵ (>0), the Rips complex is a simplicial complex made up of vertices from S, with simplices formed from finite subsets of S, where each pair of points in a given simplex has a distance at most 2ϵ.[34,113] The radius ϵ has been chosen as the filtration parameter because it is standard in PH and represents the actual underlying metric distances that directly correspond to correlations between brain regions. Therefore, this choice captures the underlying topological structure of the data. Given a simplicial complex K, a filtration of length n is a nested sequence of subcomplexes, where each subcomplex is contained within the next. Formally, it is defined as

$$K_0 \subseteq K_1 \subseteq K_2 \subseteq \ldots \subseteq K_n = K.$$

In this sequence, K_i represents a simplicial complex at the i th step. Unique values from the distance matrix serve as a filtration parameter of the resulting sequence of Rips complexes. Further details regarding simplicial complexes, Rips complexes, and filtrations can be found in the supplemental information.

Homology is a fundamental concept in algebraic topology that studies the shape of a space by identifying its topological features, often known as "holes" of different dimensions. These features include the zeroth homology group H_0, representing connected components, the first homology group H_1, representing loops or one-dimensional holes, the second homology group H_2, representing voids or two-dimensional holes, and so forth in the simplicial complex.[34,113] It provides a way to characterize topological features that remain invariant under continuous transformations like stretching, bending, or squishing. PH extends this idea by analyzing the span of important topological features of a simplicial complex throughout the filtration process.[27,116,117] Given a filtered simplicial complex built from data, PH tracks the birth and death of the features, capturing their persistence. Detailed mathematical expressions of homology and PH are included in the supplemental information.

Topological measures

Persistent entropy

The span of topological features is effectively visualized using persistence barcodes, which distill complex high-dimensional data into a straightforward interpretable format. In the filtration

of a finite simplicial complex K, the pth barcode diagram represents the birth and death of p holes during the filtration process.[30] In particular, for a Rips filtration, a barcode spanning from b to d on the x axis depicts a p hole in K, with b and d representing its birth and death filtration values, respectively.

Persistent entropy is a summary statistic of the persistence barcodes that quantifies the spans of the topological features from a sequence of birth-death information.[77,118,119] In particular, it provides the Shannon entropy of the persistence barcodes. Consider a persistence diagram $PD = \{(b_i,d_i)\}$, where (b_i,d_i) represents the birth and death filtration values, with each $d_i<+\infty$. The persistent entropy associated with PD is then defined as

$$PE(PD) = -\sum_{i=1}^{n} e_i \log(e_i),$$

where

$$e_i = \frac{(d_i - b_i)}{L} \text{ and } L = \sum_{i=1}^{n}(d_i - b_i),$$

with n denoting the total number of bars and $0 \leq PE(PD) \leq \log(n)$. Persistent entropy shows how the spans of the features are spread out. A higher entropy value indicates that the spans of features are more evenly distributed.

L^p-norm of persistence landscape

Like barcodes, a persistence diagram also visually represents the span of the features. It represents a multiset of points $\{(b_i,d_i)\}$ in $\mathbb{R}^2$, indicating the birth and death pairs for each features.[34] To perform statistical analysis, a persistence diagram is transformed into a sequence of real-valued functions $\Lambda_i : \mathbb{R}\rightarrow\mathbb{R}$ for $i \geq 1$, known as the persistence landscape,[120] aggregating essential information contained in persistence diagrams. The k th largest value among the set $\{\Lambda_i\}$ defines the persistence landscape $\lambda_k(t)$. This study considers $k = 1$, as $\lambda_1(t)$ captures the most prominent topological features for each $t \in \mathbb{R}$. As a subset of Banach space, the persistence landscape enables the calculation of L^p-norms ($1 \leq p \leq \infty$), unlike persistence diagrams. The L^p-norms of persistence landscape $\lambda_1(t)$ are defined as[78]

$$\|\lambda_1\|_p = \left(\int_{-\infty}^{\infty} |\lambda_1(t)|^p \quad dt\right)^{1/p}.$$

Moreover, we calculate the 1-Wasserstein, 2-Wasserstein, and bottleneck distances to measure the difference between two persistence diagrams.[34] Details on persistence landscape construction and Wasserstein distance computation are provided in the supplemental information.

Node persistence and node frequency

Although PH captures multiscale topological information, it remains a global measure and limits its ability to characterize individual components' topology. In brain connectivity analysis, measures such as persistent entropy and landscapes reveal global patterns but cannot localize specific regions responsible for altered connectivity. In this section, we propose two approaches to extract node-level information from the first homology group H_1, namely node persistence and node frequency.

A one-dimensional hole is defined by its one-dimensional boundary, which is a collection of 1-simplices (or edges) that form a cycle. However, this boundary is not unique. Multiple cycles in the simplicial complex might describe the same one-dimensional hole.[31] Therefore, it is crucial to consider a representative cycle for each class. In our work, we use the representative cycles defined by JavaPlex,[88] an open-source library designed for computational topology and TDA, which is integrated into our workflow via the Jython interface.

Let $G = \{g_1, g_2, \ldots, g_{\beta_1}\}$ be the set of all one-dimensional holes, where β_1 denotes the number of one-dimensional holes and the ith one-dimensional hole is associated with the birth-death pair (b_i,d_i). The representative cycle of g_i is defined by the set of nodes $\{v_i^1, v_i^2, v_i^3, \ldots, v_i^m\}$. Furthermore, we use the term "persistence extent" to characterize the overall spread or range of persistence barcodes.

The node persistence of a node v is defined by aggregating the spans of all cycles that include the node over the persistence extent of the one-dimensional holes. Mathematically, it can be expressed as

$$NP(v) = \frac{\sum_{g_i|v \in g_i}(d_i - b_i)}{\max\{d_i \;|1 \leq i \leq \beta_1\} - \min\{b_i|1 \leq i \leq \beta_1\}}.$$

The node frequency of a node v provides the number of distinct cycles containing the node. Mathematically, it can be expressed as

$$NF(v) = |\{g_i|v \in g_i\}|.$$

The two measures, node persistence and node frequency, yield node-level or local insights into PH while taking the one-dimensional holes into account.

PH-based analysis of brain functional connectivity

We consider FC matrices from two datasets to conduct a PH-based analysis of brain connectivity, namely MPI-LEMON comprising 225 subjects and ABIDE-I comprising 820 subjects. In particular, we employed topological measures derived from PH to compare resting-state functional connectivity between young and elderly groups in the MPI-LEMON dataset and between individuals with ASD and TD controls in the ABIDE-I dataset.

Brain-wide analysis

To identify brain-wide or global changes in resting-state functional connectivity, we utilized the 200 × 200 FC matrix for each subject. Each FC matrix was transformed into a distance matrix according to the procedure outlined in the section "construction of Rips complex from FC matrix and PH." Subsequently, we constructed a filtration of Rips complexes over each distance matrix, indexed by the set of unique pairwise distances serving as the filtration parameters. Our analysis focused on the evolution of topological features as distance varies, captured by PH. We calculated six global topological measures: (1) persistent entropy from the persistence barcodes associated with H_0, H_1, and H_2[77]; (2) L^1-norm of the persistence landscape associated with H_1[120]; (3) L^2-norm of the persistence landscape associated with H_1; (4) the 1-Wasserstein distance; (5) the 2-Wasserstein distance; and (6) the bottleneck distance between the persistence diagrams associated with H_1. We utilized the open-source library Gudhi[121] to compute these global topological measures.

RSN-level analysis

As mentioned previously, the Schaefer atlas[74] consists of 200 ROIs or nodes partitioned across seven RSNs. To assess

RSN-level changes in functional connectivity, we extracted submatrices from the FC matrix containing connections between nodes within each RSN and converted them into distance matrices using Equation 1. Consequently, for each subject, this yielded seven distance matrices, each corresponding to one of the seven RSNs. This results in 225 $\times$ 7 = 1,575 and 820 $\times$ 7 = 5,740 distance matrices for the MPI-LEMON and ABIDE-I datasets, respectively. The size of each distance matrix varies based on the number of nodes present in the respective RSN. Finally, Rips filtrations for each RSN were constructed using only intra-RSN connections, following the same approach as the brain-wide analysis. The six topological measures listed in brain-wide analysis were also calculated to capture RSN-level functional connectivity changes. These include persistent entropy, L^1-norm, L^2-norm, and 1-Wasserstein, 2-Wasserstein, and bottleneck distances.

ROI-level analysis

To identify ROI-level or local changes in resting-state functional connectivity, we utilized the filtration of Rips complexes generated during the brain-wide analysis. Subsequently, we calculated three topological measures based on PH: (1) node persistence, (2) node frequency, and (3) nodal PSS. These measures characterize node-level topological information by extracting the representative cycles of the first homology group H_1, which corresponds to one-dimensional holes or cycles within the Rips complex. Node persistence and node frequency were computed via JavaPlex,[88] while nodal PSS was computed using the Python package Holes.[51,122]

Neurosynth meta-analysis decoding

Using the ROI-level analysis described in the preceding section, we identified specific brain regions associated with altered resting-state functional connectivity in healthy aging (based on the MPI-LEMON dataset) as well as during ASD (based on the ABIDE-I dataset). The cognitive and behavioral implications of these findings were then examined through Neurosynth meta-analysis decoding.[72,73] First, we used the Neurosynth meta-analysis tool to identify the terms that are related to behavior, cognition, and perception based on the centroid coordinates of the 200 ROIs in the Schaefer atlas. Second, we focused on nodes exhibiting significant inter-group differences in node persistence values. Each ROI or node belongs to one of the seven RSNs, as specified by the Schaefer atlas. Third, we computed the frequency of terms associated with significant nodes within each RSN. The statistical significance of these frequency counts was assessed using a procedure described in the following subsection.

Statistical analyses

We performed two-tailed two-sample t tests[79] to examine the statistical significance of the differences in the means of the measured values between (1) young and elderly groups in the MPI-LEMON dataset and (2) ASD and TD groups in the ABIDE-I dataset. To evaluate group-level differences at global and mesoscopic scales, comparisons were performed across three measures: persistent entropy, L^1-norm, and L^2-norm. For 1-Wasserstein, 2-Wasserstein, and bottleneck distances, one-tailed two-sample t tests were used to test whether intra-group distances were smaller than inter-group distances. Furthermore, to evaluate group-level differences at the local scale, two-tailed two-sample t tests were performed across all 200 nodes for node persistence, node frequency, and nodal PSS.

To assess the statistical significance of the frequency of terms in the Neurosynth meta-analysis decoding, we compared the occurrences of the same terms within an equal number of randomly chosen nodes (proxy ROIs). For instance, considering node persistence, we obtained 108 nodes that exhibit significant differences between the young and elderly groups in the MPI-LEMON dataset. Therefore, from the 200 nodes in the Schaefer atlas, we randomly considered 108 nodes. This allowed us to establish a null distribution for term occurrences by generating 1,000 sets of these randomly selected nodes. We then determined the Z score for each term's frequency in the original set of ROIs. Thereafter, we converted these Z scores into p values, assuming a normal distribution.[69,71,73]

To account for multiple comparisons and reduce the likelihood of false positives, we implemented an FDR correction[123] to adjust the p values. We conducted all statistical tests using the SciPy[124] and statsmodels[125] packages in Python. Moreover, the analyses were performed without incorporating any covariates such as gender or age.

Estimating overlap between brain regions identified by ROI-level analysis and non-invasive stimulation studies

We systematically compared ROIs showing significant between-group differences in local topological measures with those identified in NIBS studies, focusing on three NIBS modalities: tDCS, tACS, and TMS. To compare with brain regions exhibiting functional connectivity changes, we focused on NIBS target regions that improved motor performance in healthy elderly individuals for age-related changes and alleviated ASD symptoms for ASD-related changes. We evaluated the overlap of these target regions with those identified by node persistence, node frequency, and nodal PSS. These overlaps allowed us to assess whether local topological measures of resting-state functional connectivity could identify brain regions whose non-invasive stimulation yields functional benefits in healthy aging and ASD.

The target regions of NIBS experiments were acquired based on a systematic literature review carried out in earlier studies by some of us.[69,71] These target regions, which originally correspond to regions in the Brodmann atlas,[126] were mapped to regions in the Schaefer atlas. This mapping is available at https://github.com/asamallab/Curvature-FCN-ASD.

RESOURCE AVAILABILITY

Lead contact

Requests for further information and resources should be directed to and will be fulfilled by the lead contact, Areejit Samal (asamal@imsc.res.in).

Materials availability

This study did not generate new materials.

Data and code availability

- The functional connectivity matrices considered in our study were downloaded directly from two GitHub repositories and are publicly available at https://github.com/asamallab/Curvature-FCN-Aging[69] and https://github.com/asamallab/Curvature-FCN-ASD.[71]
- The code for computing node persistence and node frequency, as well as the code for the analysis and to reproduce the results in this paper, is

publicly accessible through the GitHub repository (https://github.com/asamallab/NodePersistence_PH_FCM) and has been archived at Zenodo.[127]

- Any additional information required to reanalyze the data reported in this paper is available from the lead contact upon request.

ACKNOWLEDGMENTS

The authors thank Kelin Xia for insightful discussions. A.S. acknowledges funding from the Department of Atomic Energy, Government of India (via the Apex project to The Institute of Mathematical Sciences [IMSc], Chennai) and funding from the Max Planck Society, Germany (through the award of a Max Planck Partner Group).

AUTHOR CONTRIBUTIONS

Conceptualization, M.M., Y.Y., J.J., and A.S.; methodology, M.M., Y.Y., J.J., and A.S.; investigation, M.M., Y.Y., J.J., and A.S.; visualization, M.M.; writing – original draft, M.M., Y.Y., J.J., and A.S.; writing – review & editing, M.M., Y.Y., J.J., and A.S.; funding acquisition, A.S.; resources, M.M., Y.Y., J.J., and A.S.; supervision, J.J. and A.S.

DECLARATION OF INTERESTS

The authors declare no competing interests.

SUPPLEMENTAL INFORMATION

Supplemental information can be found online at https://doi.org/10.1016/j.patter.2025.101427.

Received: June 12, 2025
Revised: September 23, 2025
Accepted: October 28, 2025
Published: December 3, 2025

REFERENCES

1. Friston, K.J., Frith, C.D., Liddle, P.F., and Frackowiak, R.S. (1993). Functional connectivity: the principal-component analysis of large (PET) data sets. J. Cereb. Blood Flow Metab. *13*, 5–14. https://doi.org/10.1038/jcbfm.1993.4.
2. Ogawa, S., Lee, T.-M., Kay, A.R., and Tank, D.W. (1990). Brain magnetic resonance imaging with contrast dependent on blood oxygenation. Proc. Natl. Acad. Sci. USA *87*, 9868–9872. https://doi.org/10.1073/pnas.87.24.9868.
3. Logothetis, N.K. (2008). What we can do and what we cannot do with fMRI. Nature *453*, 869–878. https://doi.org/10.1038/nature06976.
4. Hlinka, J., Paluš, M., Vejmelka, M., Mantini, D., and Corbetta, M. (2011). Functional connectivity in resting-state fMRI: is linear correlation sufficient? Neuroimage *54*, 2218–2225. https://doi.org/10.1016/j.neuroimage.2010.08.042.
5. Biswal, B., Yetkin, F.Z., Haughton, V.M., and Hyde, J.S. (1995). Functional connectivity in the motor cortex of resting human brain using echo-planar MRI. Magn. Reson. Med. *34*, 537–541. https://doi.org/10.1002/mrm.1910340409.
6. Raichle, M.E., MacLeod, A.M., Snyder, A.Z., Powers, W.J., Gusnard, D.A., and Shulman, G.L. (2001). A default mode of brain function. Proc. Natl. Acad. Sci. USA *98*, 676–682. https://doi.org/10.1073/pnas.98.2.676.
7. Greicius, M.D., Krasnow, B., Reiss, A.L., and Menon, V. (2003). Functional connectivity in the resting brain: A network analysis of the default mode hypothesis. Proc. Natl. Acad. Sci. USA *100*, 253–258. https://doi.org/10.1073/pnas.0135058100.
8. Smith, S.M., Fox, P.T., Miller, K.L., Glahn, D.C., Fox, P.M., Mackay, C.E., Filippini, N., Watkins, K.E., Toro, R., Laird, A.R., and Beckmann, C.F. (2009). Correspondence of the brain's functional architecture during activation and rest. Proc. Natl. Acad. Sci. USA *106*, 13040–13045. https://doi.org/10.1073/pnas.0905267106.
9. Fox, M.D., and Raichle, M.E. (2007). Spontaneous fluctuations in brain activity observed with functional magnetic resonance imaging. Nat. Rev. Neurosci. *8*, 700–711. https://doi.org/10.1038/nrn2201.
10. Yeo, B.T.T., Krienen, F.M., Sepulcre, J., Sabuncu, M.R., Lashkari, D., Hollinshead, M., Roffman, J.L., Smoller, J.W., Zöllei, L., Polimeni, J.R., et al. (2011). The organization of the human cerebral cortex estimated by intrinsic functional connectivity. J. Neurophysiol. *106*, 1125–1165. https://doi.org/10.1152/jn.00338.2011.
11. Grady, C. (2012). The cognitive neuroscience of ageing. Nat. Rev. Neurosci. *13*, 491–505. https://doi.org/10.1038/nrn3256.
12. Tromp, D., Dufour, A., Lithfous, S., Pebayle, T., and Després, O. (2015). Episodic memory in normal aging and Alzheimer disease: Insights from imaging and behavioral studies. Ageing Res. Rev. *24*, 232–262. https://doi.org/10.1016/j.arr.2015.08.006.
13. Nyberg, L., Salami, A., Andersson, M., Eriksson, J., Kalpouzos, G., Kauppi, K., Lind, J., Pudas, S., Persson, J., and Nilsson, L.-G. (2010). Longitudinal evidence for diminished frontal cortex function in aging. Proc. Natl. Acad. Sci. USA *107*, 22682–22686. https://doi.org/10.1073/pnas.1012651108.
14. Lord, C., Rutter, M., and Le Couteur, A. (1994). Autism Diagnostic Interview-Revised: A revised version of a diagnostic interview for caregivers of individuals with possible pervasive developmental disorders. J. Autism Dev. Disord. *24*, 659–685. https://doi.org/10.1007/BF02172145.
15. Lord, C., Brugha, T.S., Charman, T., Cusack, J., Dumas, G., Frazier, T., Jones, E.J.H., Jones, R.M., Pickles, A., State, M.W., et al. (2020). Autism spectrum disorder. Nat. Rev. Dis. Primers *6*, 5. https://doi.org/10.1038/s41572-019-0138-4.
16. National Institute of Neurological Disorders and Stroke. Autism Spectrum Disorder Fact Sheet (2020). https://www.nimh.nih.gov/health/publications/autism-spectrum-disorder.
17. Kristen, S., Rossmann, F., and Sodian, B. (2014). Theory of own mind and autobiographical memory in adults with ASD. Res. Autism Spectr. Dis. *8*, 827–837. https://doi.org/10.1016/j.rasd.2014.03.009.
18. Habib, A., Harris, L., Pollick, F., and Melville, C. (2019). A meta-analysis of working memory in individuals with autism spectrum disorders. PLoS One *14*, e0216198. https://doi.org/10.1371/journal.pone.0216198.
19. Zampella, C.J., Wang, L.A.L., Haley, M., Hutchinson, A.G., and de Marchena, A. (2021). Motor Skill Differences in Autism Spectrum Disorder: a Clinically Focused Review. Curr. Psychiatry Rep. *23*, 64. https://doi.org/10.1007/s11920-021-01280-6.
20. Arora, N.K., Nair, M.K.C., Gulati, S., Deshmukh, V., Mohapatra, A., Mishra, D., Patel, V., Pandey, R.M., Das, B.C., Divan, G., et al. (2018). Neurodevelopmental disorders in children aged 2–9 years: Population-based burden estimates across five regions in India. PLoS Med. *15*, e1002615. https://doi.org/10.1371/journal.pmed.1002615.
21. Maenner, M.J., Shaw, K.A., Baio, J., Washington, A., Patrick, M., DiRienzo, M., Christensen, D.L., Wiggins, L.D., Pettygrove, S., Andrews, J.G., et al. (2020). Prevalence of autism spectrum disorder among children aged 8 years – autism and developmental disabilities monitoring network, 11 sites, United States, 2016. MMWR. Surveill. Summ. *69*, 1–12. https://doi.org/10.15585/mmwr.ss6904a1.
22. Fein, D., Barton, M., Eigsti, I.-M., Kelley, E., Naigles, L., Schultz, R.T., Stevens, M., Helt, M., Orinstein, A., Rosenthal, M., et al. (2013). Optimal outcome in individuals with a history of autism. J. Child Psychol. Psychiatry *54*, 195–205. https://doi.org/10.1111/jcpp.12037.
23. Spreng, R.N., Wojtowicz, M., and Grady, C.L. (2010). Reliable differences in brain activity between young and old adults: A quantitative meta-analysis across multiple cognitive domains. Neurosci. Biobehav. Rev. *34*, 1178–1194. https://doi.org/10.1016/j.neubiorev.2010.01.009.

24. Woodward, N.D., and Cascio, C.J. (2015). Resting-State Functional Connectivity in Psychiatric Disorders. JAMA Psychiatry *72*, 743–744. https://doi.org/10.1001/jamapsychiatry.2015.0484.

25. Solso, S., Xu, R., Proudfoot, J., Hagler, D.J., Campbell, K., Venkatraman, V., Carter Barnes, C., Ahrens-Barbeau, C., Pierce, K., Dale, A., et al. (2016). Diffusion Tensor Imaging Provides Evidence of Possible Axonal Overconnectivity in Frontal Lobes in Autism Spectrum Disorder Toddlers. Biol. Psychiatry *79*, 676–684. https://doi.org/10.1016/j.biopsych.2015.06.029.

26. Chazal, F., and Michel, B. (2021). An introduction to topological data analysis: fundamental and practical aspects for data scientists. Front. Artif. Intell. *4*, 667963. https://doi.org/10.3389/frai.2021.667963.

27. Edelsbrunner, H., Letscher, D., and Zomorodian, A. (2002). Topological Persistence and Simplification. Discrete Comput. Geom. *28*, 511–533. https://doi.org/10.1007/s00454-002-2885-2.

28. Patania, A., Vaccarino, F., and Petri, G. (2017). Topological analysis of data. EPJ Data Sci. *6*, 7. https://doi.org/10.1140/epjds/s13688-017-0104-x.

29. Munkres, J.R. (2018). Elements of Algebraic Topology (CRC Press).

30. Ghrist, R. (2007). Barcodes: The persistent topology of data. Bull. Amer. Math. Soc. *45*, 61–76. https://doi.org/10.1090/S0273-0979-07-01191-3.

31. Edelsbrunner, H., and Harer, J. (2008). Persistent Homology – a Survey. Contemp. Math. *453*, 257–282.

32. Carlsson, G. (2009). Topology and data. Bull. Amer. Math. Soc. *46*, 255–308. https://doi.org/10.1090/S0273-0979-09-01249-X.

33. Otter, N., Porter, M.A., Tillmann, U., Grindrod, P., and Harrington, H.A. (2017). A roadmap for the computation of persistent homology. EPJ Data Sci. *6*, 17. https://doi.org/10.1140/epjds/s13688-017-0109-5.

34. Edelsbrunner, H., and Harer, J. (2010). Computational Topology: An Introduction (American Mathematical Society).

35. Chan, J.M., Carlsson, G., and Rabadan, R. (2013). Topology of viral evolution. Proc. Natl. Acad. Sci. USA *110*, 18566–18571. https://doi.org/10.1073/pnas.1313480110.

36. Topaz, C.M., Ziegelmeier, L., and Halverson, T. (2015). Topological data analysis of biological aggregation models. PLoS One *10*, e0126383. https://doi.org/10.1371/journal.pone.0126383.

37. Cámara, P.G., Levine, A.J., and Rabadán, R. (2016). Inference of Ancestral Recombination Graphs through Topological Data Analysis. PLoS Comput. Biol. *12*, e1005071. https://doi.org/10.1371/journal.pcbi.1005071.

38. Xia, K., Li, Z., and Mu, L. (2018). Multiscale Persistent Functions for Biomolecular Structure Characterization. Bull. Math. Biol. *80*, 1–31. https://doi.org/10.1007/s11538-017-0362-6.

39. Gidea, M. (2017). Topological Data Analysis of Critical Transitions in Financial Networks. In 3rd International Winter School and Conference on Network Science: NetSci-X 2017, pp. 47–59. https://doi.org/10.1007/978-3-319-55471-6_5.

40. Gidea, M., and Katz, Y. (2018). Topological data analysis of financial time series: Landscapes of crashes. Phys. Stat. Mech. Appl. *491*, 820–834. https://doi.org/10.1016/j.physa.2017.09.028.

41. Guo, H., Zhao, X., Yu, H., and Zhang, X. (2021). Analysis of global stock markets' connections with emphasis on the impact of COVID-19. Physica A *569*, 125774. https://doi.org/10.1016/j.physa.2021.125774.

42. Kulkarni, S., Pharasi, H.K., Vijayaraghavan, S., Kumar, S., Chakraborti, A., and Samal, A. (2024). Investigation of Indian stock markets using topological data analysis and geometry-inspired network measures. Phys. Stat. Mech. Appl. *643*, 129785. https://doi.org/10.1016/j.physa.2024.129785.

43. Kramar, M., Goullet, A., Kondic, L., and Mischaikow, K. (2013). Persistence of force networks in compressed granular media. Phys. Rev. *87*, 042207. https://doi.org/10.1103/PhysRevE.87.042207.

44. Donato, I., Gori, M., Pettini, M., Petri, G., De Nigris, S., Franzosi, R., and Vaccarino, F. (2016). Persistent homology analysis of phase transitions. Phys. Rev. E *93*, 052138. https://doi.org/10.1103/PhysRevE.93.052138.

45. Heydenreich, S., Brück, B., and Harnois-Déraps, J. (2021). Persistent homology in cosmic shear: Constraining parameters with topological data analysis. Astron. Astrophys. *648*, A74. https://doi.org/10.1051/0004-6361/202039048.

46. Adams, H., Emerson, T., Kirby, M., Neville, R., Peterson, C., Shipman, P., Chepushtanova, S., Hanson, E., Motta, F., and Ziegelmeier, L. (2017). Persistence images: a stable vector representation of persistent homology. J. Mach. Learn. Res. *18*, 218–252. https://dl.acm.org/doi/10.5555/3122009.3122017.

47. Townsend, J., Micucci, C.P., Hymel, J.H., Maroulas, V., and Vogiatzis, K.D. (2020). Representation of molecular structures with persistent homology for machine learning applications in chemistry. Nat. Commun. *11*, 3230. https://doi.org/10.1038/s41467-020-17035-5.

48. Pun, C.S., Lee, S.X., and Xia, K. (2022). Persistent-homology-based machine learning: a survey and a comparative study. Artif. Intell. Rev. *55*, 5169–5213. https://doi.org/10.1007/s10462-022-10146-z.

49. Vandaele, R., Nervo, G.A., and Gevaert, O. (2020). Topological image modification for object detection and topological image processing of skin lesions. Sci. Rep. *10*, 21061. https://doi.org/10.1038/s41598-020-77933-y.

50. Edelsbrunner, H. (2013). Persistent homology in image processing. In Graph-Based Representations in Pattern Recognition, W.G. Kropatsch, N.M. Artner, Y. Haxhimusa, and X. Jiang, eds., pp. 182–183. https://doi.org/10.1007/978-3-642-38221-5_19.

51. Petri, G., Expert, P., Turkheimer, F., Carhart-Harris, R., Nutt, D., Hellyer, P.J., and Vaccarino, F. (2014). Homological scaffolds of brain functional networks. J. R. Soc. Interface *11*, 20140873. https://doi.org/10.1098/rsif.2014.0873.

52. Lord, L.-D., Expert, P., Fernandes, H.M., Petri, G., Van Hartevelt, T.J., Vaccarino, F., Deco, G., Turkheimer, F., and Kringelbach, M.L. (2016). Insights into Brain Architectures from the Homological Scaffolds of Functional Connectivity Networks. Front. Syst. Neurosci. *10*, 85. https://doi.org/10.3389/fnsys.2016.00085.

53. Giusti, C., Ghrist, R., and Bassett, D.S. (2016). Two's company, three (or more) is a simplex. J. Comput. Neurosci. *41*, 1–14. https://doi.org/10.1007/s10827-016-0608-6.

54. Bendich, P., Marron, J.S., Miller, E., Pieloch, A., and Skwerer, S. (2016). Persistent homology analysis of brain artery trees. Ann. Appl. Stat. *10*, 198–218. https://doi.org/10.1214/15-AOAS886.

55. Chung, M.K., Lee, H., DiChristofano, A., Ombao, H., and Solo, V. (2019). Exact topological inference of the resting-state brain networks in twins. Netw. Neurosci. *3*, 674–694. https://doi.org/10.1162/netn_a_00091.

56. Kuang, L., Han, X., Chen, K., Caselli, R.J., Reiman, E.M., and Wang, Y.; Alzheimer's Disease Neuroimaging Initiative (2019). A concise and persistent feature to study brain resting-state network dynamics: Findings from the Alzheimer's Disease Neuroimaging Initiative. Hum. Brain Mapp. *40*, 1062–1081. https://doi.org/10.1002/hbm.24383.

57. Liang, D., Xia, S., Zhang, X., and Zhang, W. (2021). Analysis of Brain Functional Connectivity Neural Circuits in Children With Autism Based on Persistent Homology. Front. Hum. Neurosci. *15*, 745671. https://doi.org/10.3389/fnhum.2021.745671.

58. Talesh Jafadideh, A., and Mohammadzadeh Asl, B. (2022). Topological analysis of brain dynamics in autism based on graph and persistent homology. Comput. Biol. Med. *150*, 106202. https://doi.org/10.1016/j.compbiomed.2022.106202.

59. Xing, J., Jia, J., Wu, X., and Kuang, L. (2022). A Spatiotemporal Brain Network Analysis of Alzheimer's Disease Based on Persistent Homology. Front. Aging Neurosci. *14*, 788571. https://doi.org/10.3389/fnagi.2022.788571.

60. Zhang, W., Xia, S., Tang, X., Zhang, X., Liang, D., and Wang, Y. (2023). Topological analysis of functional connectivity in Parkinson's disease. Front. Neurosci. *17*, 1236128. https://doi.org/10.3389/fnins.2023.1236128.

CellPress
OPEN ACCESS

61. Ryu, H., Habeck, C., Stern, Y., and Lee, S. (2023). Persistent homology-based functional connectivity and its association with cognitive ability: Life-span study. Hum. Brain Mapp. *44*, 3669–3683. https://doi.org/10.1002/hbm.26304.

62. Mijangos, M., Pacheco, L., Bravetti, A., González-García, N., Padilla, P., and Velasco-Segura, R. (2024). Persistent homology reveals robustness loss in inhaled substance abuse rs-fMRI networks. PLoS One *19*, e0310165. https://doi.org/10.1371/journal.pone.0310165.

63. Chung, M.K., Huang, S.-G., Carroll, I.C., Calhoun, V.D., and Goldsmith, H.H. (2024). Topological state-space estimation of functional human brain networks. PLoS Comput. Biol. *20*, e1011869. https://doi.org/10.1371/journal.pcbi.1011869.

64. Aggarwal, M., and Periwal, V. (2023). Tight basis cycle representatives for persistent homology of large biological data sets. PLoS Comput. Biol. *19*, e1010341. https://doi.org/10.1371/journal.pcbi.1010341.

65. Bendich, P., Gasparovic, E., Harer, J., Izmailov, R., and Ness, L. (2015). Multi-scale local shape analysis and feature selection in machine learning applications. In 2015 International Joint Conference on Neural Networks (IJCNN), pp. 1–8. https://doi.org/10.1109/IJCNN.2015.7280428.

66. Fasy, B.T., and Wang, B. (2016). Exploring persistent local homology in topological data analysis. In 2016 IEEE International Conference on Acoustics, Speech and Signal Processing (ICASSP), pp. 6430–6434. https://doi.org/10.1109/ICASSP.2016.7472915.

67. Nguyen, N., Hou, T., Amico, E., Zheng, J., Huang, H., Kaplan, A.D., Petri, G., Goñi, J., Kaufmann, R., Zhao, Y., et al. (2024). Volume-Optimal Persistence Homological Scaffolds of Hemodynamic Networks Covary with MEG Theta-Alpha Aperiodic Dynamics. In Medical Image Computing and Computer Assisted Intervention – MICCAI 2024, pp. 519–529. https://doi.org/10.1007/978-3-031-72384-1_49.

68. Babayan, A., Erbey, M., Kumral, D., Reinelt, J.D., Reiter, A.M.F., Röbbig, J., Schaare, H.L., Uhlig, M., Anwander, A., Bazin, P.-L., et al. (2019). A mind-brain-body dataset of MRI, EEG, cognition, emotion, and peripheral physiology in young and old adults. Sci. Data *6*, 180308. https://doi.org/10.1038/sdata.2018.308.

69. Yadav, Y., Elumalai, P., Williams, N., Jost, J., and Samal, A. (2023). Discrete Ricci curvatures capture age-related changes in human brain functional connectivity networks. Front. Aging Neurosci. *15*, 1120846. https://doi.org/10.3389/fnagi.2023.1120846.

70. Di Martino, A., Yan, C.-G., Li, Q., Denio, E., Castellanos, F.X., Alaerts, K., Anderson, J.S., Assaf, M., Bookheimer, S.Y., Dapretto, M., et al. (2014). The autism brain imaging data exchange: towards a large-scale evaluation of the intrinsic brain architecture in autism. Mol. Psychiatry *19*, 659–667. https://doi.org/10.1038/mp.2013.78.

71. Elumalai, P., Yadav, Y., Williams, N., Saucan, E., Jost, J., and Samal, A. (2022). Graph Ricci curvatures reveal atypical functional connectivity in autism spectrum disorder. Sci. Rep. *12*, 8295. https://doi.org/10.1038/s41598-022-12171-y.

72. Yarkoni, T., Poldrack, R.A., Nichols, T.E., Van Essen, D.C., and Wager, T.D. (2011). Large-scale automated synthesis of human functional neuroimaging data. Nat. Methods *8*, 665–670. https://doi.org/10.1038/nmeth.1635.

73. Williams, N., Wang, S.H., Arnulfo, G., Nobili, L., Palva, S., and Palva, J.M. (2023). Modules in connectomes of phase-synchronization comprise anatomically contiguous, functionally related regions. Neuroimage *272*, 120036. https://doi.org/10.1016/j.neuroimage.2023.120036.

74. Schaefer, A., Kong, R., Gordon, E.M., Laumann, T.O., Zuo, X.-N., Holmes, A.J., Eickhoff, S.B., and Yeo, B.T.T. (2018). Local-Global Parcellation of the Human Cerebral Cortex from Intrinsic Functional Connectivity MRI. Cereb. Cortex *28*, 3095–3114. https://doi.org/10.1093/cercor/bhx179.

75. Mantegna, R.N. (1999). Hierarchical structure in financial markets. Eur. Phys. J. B *11*, 193–197. https://doi.org/10.1007/s100510050929.

76. Qian, J., Diez, I., Ortiz-Terán, L., Bonadio, C., Liddell, T., Goñi, J., and Sepulcre, J. (2018). Positive Connectivity Predicts the Dynamic Intrinsic Topology of the Human Brain Network. Front. Syst. Neurosci. *12*, 38. https://doi.org/10.3389/fnsys.2018.00038.

77. Chintakunta, H., Gentimis, T., Gonzalez-Diaz, R., Jimenez, M.-J., and Krim, H. (2015). An entropy-based persistence barcode. Pattern Recogn. *48*, 391–401. https://doi.org/10.1016/j.patcog.2014.06.023.

78. Bubenik, P. (2020). The persistence landscape and some of its properties. In Topological Data Analysis, N. Baas, G. Carlsson, G. Quick, M. Szymik, and M. Thaule, eds., pp. 97–117. https://doi.org/10.1007/978-3-030-43408-3_4.

79. Yuen, K.K. (1974). The two-sample trimmed t for unequal population variances. Biometrika *61*, 165–170. https://doi.org/10.1093/biomet/61.1.165.

80. Xia, M., Wang, J., and He, Y. (2013). BrainNet Viewer: A Network Visualization Tool for Human Brain Connectomics. PLoS One *8*, e68910. https://doi.org/10.1371/journal.pone.0068910.

81. Benkarim, O., Paquola, C., Park, B.-y., Hong, S.-J., Royer, J., Vos de Wael, R., Lariviere, S., Valk, S., Bzdok, D., Mottron, L., and C Bernhardt, B. (2021). Connectivity alterations in autism reflect functional idiosyncrasy. Commun. Biol. *4*, 1078. https://doi.org/10.1038/s42003-021-02572-6.

82. Guo, Z., Tang, X., Xiao, S., Yan, H., Sun, S., Yang, Z., Huang, L., Chen, Z., and Wang, Y. (2024). Systematic review and meta-analysis: multimodal functional and anatomical neural alterations in autism spectrum disorder. Mol. Autism *15*, 16. https://doi.org/10.1186/s13229-024-00593-6.

83. Picci, G., Gotts, S.J., and Scherf, K.S. (2016). A theoretical rut: revisiting and critically evaluating the generalized under/over-connectivity hypothesis of autism. Dev. Sci. *19*, 524–549. https://doi.org/10.1111/desc.12467.

84. Bjørklund, G., Meguid, N.A., El-Ansary, A., El-Bana, M.A., Dadar, M., Aaseth, J., Hemimi, M., Osredkar, J., and Chirumbolo, S. (2018). Diagnostic and Severity-Tracking Biomarkers for Autism Spectrum Disorder. J. Mol. Neurosci. *66*, 492–511. https://doi.org/10.1007/s12031-018-1192-1.

85. Hiremath, C.S., Sagar, K.J.V., Yamini, B.K., Girimaji, A.S., Kumar, R., Sravanti, S.L., Padmanabha, H., Vykunta Raju, K.N., Kishore, M.T., Jacob, P., et al. (2021). Emerging behavioral and neuroimaging biomarkers for early and accurate characterization of autism spectrum disorders: a systematic review. Transl. Psychiatry *11*, 42. https://doi.org/10.1038/s41398-020-01178-6.

86. Lenroot, R.K., and Yeung, P.K. (2013). Heterogeneity within Autism Spectrum Disorders: What have We Learned from Neuroimaging Studies? Front. Hum. Neurosci. *7*, 733. https://doi.org/10.3389/fnhum.2013.00733.

87. Waizbard-Bartov, E., and Miller, M. (2023). Does the severity of autism symptoms change over time? A review of the evidence, impacts, and gaps in current knowledge. Clin. Psychol. Rev. *99*, 102230. https://doi.org/10.1016/j.cpr.2022.102230.

88. Adams, H., Tausz, A., Johansson, V.-, and JavaPlex, M. (2014). A research software package for persistent (co)homology. In Mathematical Software–ICMS 2014 (Springer), pp. 129–136. https://doi.org/10.1007/978-3-662-44199-2_23.

89. Krassowski, M. ComplexUpset (2020). https://github.com/krassowski/complex-upset.

90. Habib, E.A.E. (2012). Mean absolute deviation about median as a tool of explanatory data analysis. Int. J. Res. Rev. Appl. Sci. *11*, 517–523.

91. Leys, C., Ley, C., Klein, O., Bernard, P., and Licata, L. (2013). Detecting outliers: Do not use standard deviation around the mean, use absolute deviation around the median. J. Exp. Soc. Psychol. *49*, 764–766. https://doi.org/10.1016/j.jesp.2013.03.013.

92. Seidler, R.D., Bernard, J.A., Burutolu, T.B., Fling, B.W., Gordon, M.T., Gwin, J.T., Kwak, Y., and Lipps, D.B. (2010). Motor control and aging: Links to age-related brain structural, functional, and biochemical effects. Neurosci. Biobehav. Rev. *34*, 721–733. https://doi.org/10.1016/j.neubiorev.2009.10.005.

93. Cirillo, J. (2021). Physical activity, motor performance and skill learning: a focus on primary motor cortex in healthy aging. Exp. Brain Res. *239*, 3431–3438. https://doi.org/10.1007/s00221-021-06218-1.

94. Seidler, R.D., Alberts, J.L., and Stelmach, G.E. (2002). Changes in Multi-Joint Performance with Age. Mot. Control *6*, 19–31. https://doi.org/10.1123/mcj.6.1.19.

95. Contreras-Vidal, J.L., Teulings, H.L., and Stelmach, G.E. (1998). Elderly subjects are impaired in spatial coordination in fine motor control. Acta Psychol. *100*, 25–35. https://doi.org/10.1016/S0001-6918(98)00023-7.

96. Buckles, V.D. (1993). Age-related slowing. In Sensorimotor Impairment in the Elderly (Netherlands: Springer), pp. 73–87. https://doi.org/10.1007/978-94-011-1976-4_6.

97. Schmitter-Edgecombe, M., Vesneski, M., and Jones, D.W. (2000). Aging and Word-Finding: A Comparison of Spontaneous and Constrained Naming Tests. Arch. Clin. Neuropsychol. *15*, 479–493. https://doi.org/10.1093/arclin/15.6.479.

98. Mather, M. (2012). The emotion paradox in the aging brain. Ann. N. Y. Acad. Sci. *1251*, 33–49. https://doi.org/10.1111/j.1749-6632.2012.06471.x.

99. Edwards, R.R., and Fillingim, R.B. (2001). Age-Associated Differences in Responses to Noxious Stimuli. J. Gerontol. A Biol. Sci. Med. Sci. *56*, M180–M185. https://doi.org/10.1093/gerona/56.3.M180.

100. Ming, X., Brimacombe, M., and Wagner, G.C. (2007). Prevalence of motor impairment in autism spectrum disorders. Brain Dev. *29*, 565–570. https://doi.org/10.1016/j.braindev.2007.03.002.

101. Pickles, A., Simonoff, E., Conti-Ramsden, G., Falcaro, M., Simkin, Z., Charman, T., Chandler, S., Loucas, T., and Baird, G. (2009). Loss of language in early development of autism and specific language impairment. J. Child Psychol. Psychiatry *50*, 843–852. https://doi.org/10.1111/j.1469-7610.2008.02032.x.

102. Ellis Weismer, S., Lord, C., and Esler, A. (2010). Early language patterns of toddlers on the autism spectrum compared to toddlers with developmental delay. J. Autism Dev. Disord. *40*, 1259–1273. https://doi.org/10.1007/s10803-010-0983-1.

103. Kasari, C., and Patterson, S. (2012). Interventions Addressing Social Impairment in Autism. Curr. Psychiatry Rep. *14*, 713–725. https://doi.org/10.1007/s11920-012-0317-4.

104. Senju, A. (2012). Spontaneous Theory of Mind and Its Absence in Autism Spectrum Disorders. Neuroscientist *18*, 108–113. https://doi.org/10.1177/1073858410397208.

105. Solomon, M., McCauley, J.B., Iosif, A.-M., Carter, C.S., and Ragland, J.D. (2016). Cognitive control and episodic memory in adolescents with autism spectrum disorders. Neuropsychologia *89*, 31–41. https://doi.org/10.1016/j.neuropsychologia.2016.05.013.

106. Bhat, A.N. (2021). Motor impairment increases in children with autism spectrum disorder as a function of social communication, cognitive and functional impairment, repetitive behavior severity, and comorbid diagnoses: A SPARK study report. Autism Res. *14*, 202–219. https://doi.org/10.1002/aur.2453.

107. Zhou, D., Zhou, J., Chen, H., Manor, B., Lin, J., and Zhang, J. (2015). Effects of transcranial direct current stimulation (tDCS) on multiscale complexity of dual-task postural control in older adults. Exp. Brain Res. *233*, 2401–2409. https://doi.org/10.1007/s00221-015-4310-0.

108. Yosephi, M.H., Ehsani, F., Zoghi, M., and Jaberzadeh, S. (2018). Multi-session anodal tDCS enhances the effects of postural training on balance and postural stability in older adults with high fall risk: primary motor cortex versus cerebellar stimulation. Brain Stimul. *11*, 1239–1250. https://doi.org/10.1016/j.brs.2018.07.044.

109. Kang, J.-N., Song, J.-J., Casanova, M.F., Sokhadze, E.M., and Li, X.-L. (2019). Effects of repetitive transcranial magnetic stimulation on children with low-function autism. CNS Neurosci. Ther. *25*, 1254–1261. https://doi.org/10.1111/cns.13150.

110. Hadoush, H., Nazzal, M., Almasri, N.A., Khalil, H., and Alafeef, M. (2020). Therapeutic Effects of Bilateral Anodal Transcranial Direct Current Stimulation on Prefrontal and Motor Cortical Areas in Children with Autism Spectrum Disorders: A Pilot Study. Autism Res. *13*, 828–836. https://doi.org/10.1002/aur.2290.

111. Bhattacharya, D., Kaur, R., Aithal, N., Sinha, N., and Issac, T.G. (2025). Persistent homology for MCI classification: a comparative analysis between graph and Vietoris-Rips filtrations. Front. Neurosci. *19*, 1518984. https://doi.org/10.3389/fnins.2025.1518984.

112. Tononi, G., Sporns, O., and Edelman, G.M. (1994). A measure for brain complexity: relating functional segregation and integration in the nervous system. Proc. Natl. Acad. Sci. USA *91*, 5033–5037. https://doi.org/10.1073/pnas.91.11.5033.

113. Vietoris, L. (1927). Über den höheren Zusammenhang kompakter Räume und eine Klasse von zusammenhangstreuen Abbildungen. Math. Ann. *97*, 454–472. https://doi.org/10.1007/BF01447877.

114. Whitfield-Gabrieli, S., and Nieto-Castanon, A. (2012). Conn: A Functional Connectivity Toolbox for Correlated and Anticorrelated Brain Networks. Brain Connect. *2*, 125–141. https://doi.org/10.1089/brain.2012.0073.

115. Chen, G., Chen, G., Xie, C., and Li, S.-J. (2011). Negative Functional Connectivity and Its Dependence on the Shortest Path Length of Positive Network in the Resting-State Human Brain. Brain Connect. *1*, 195–206. https://doi.org/10.1089/brain.2011.0025.

116. Robins, V. (1999). Towards computing homology from finite approximations. Topol. Proc. *24*, 503–532.

117. Zomorodian, A., and Carlsson, G. (2005). Computing Persistent Homology. Discrete Comput. Geom. *33*, 249–274. https://doi.org/10.1007/s00454-004-1146-y.

118. Rucco, M., Castiglione, F., Merelli, E., and Pettini, M. (2016). Characterisation of the idiotypic immune network through persistent entropy. In Proceedings of ECCS 2014, pp. 117–128. https://doi.org/10.1007/978-3-319-29228-1_11.

119. Atienza, N., Gonzalez-Díaz, R., and Soriano-Trigueros, M. (2020). On the stability of persistent entropy and new summary functions for topological data analysis. Pattern Recogn. *107*, 107509. https://doi.org/10.1016/j.patcog.2020.107509.

120. Bubenik, P. (2015). Statistical topological data analysis using persistence landscapes. J. Mach. Learn. Res. *16*, 77–102.

121. Maria, C., Boissonnat, J.-D., Glisse, M., and Yvinec, M. (2014). The Gudhi library: simplicial complexes and persistent homology. In Mathematical Software – ICMS 2014, H. Hong and C. Yap, eds., pp. 167–174. https://doi.org/10.1007/978-3-662-44199-2_28.

122. Guerra, M., De Gregorio, A., Fugacci, U., Petri, G., and Vaccarino, F. (2021). Homological scaffold via minimal homology bases. Sci. Rep. *11*, 5355. https://doi.org/10.1038/s41598-021-84486-1.

123. Benjamini, Y., and Hochberg, Y. (1995). Controlling the false discovery rate: a practical and powerful approach to multiple testing. J. Roy. Stat. Soc. B *57*, 289–300. https://doi.org/10.1111/j.2517-6161.1995.tb02031.x.

124. Virtanen, P., Gommers, R., Oliphant, T.E., Haberland, M., Reddy, T., Cournapeau, D., Burovski, E., Peterson, P., Weckesser, W., Bright, J., et al. (2020). SciPy 1.0: fundamental algorithms for scientific computing in Python. Nat. Methods *17*, 261–272. https://doi.org/10.1038/s41592-019-0686-2.

125. Seabold, S., and Perktold, J. (2010). Statsmodels: Econometric and Statistical Modeling with Python. In Proceedings of the 9th Python in Science Conference. https://doi.org/10.25080/Majora-92bf1922-011.

126. Strotzer, M. (2009). One century of brain mapping using Brodmann areas. Klin. Neuroradiol. *19*, 179–186. https://doi.org/10.1007/s00062-009-9002-3.

127. Mondal, M., Yadav, Y., Jost, J., and Samal, A. Data and codes for the article "Node persistence from topological data analysis reveals changes in brain functional connectivity" (2025). https://doi.org/10.5281/zenodo.17340938.

Patterns, Volume *7*

Supplemental information

Node persistence from topological data analysis reveals changes in brain functional connectivity

Madhumita Mondal, Yasharth Yadav, Jürgen Jost, and Areejit Samal

Supplemental figures

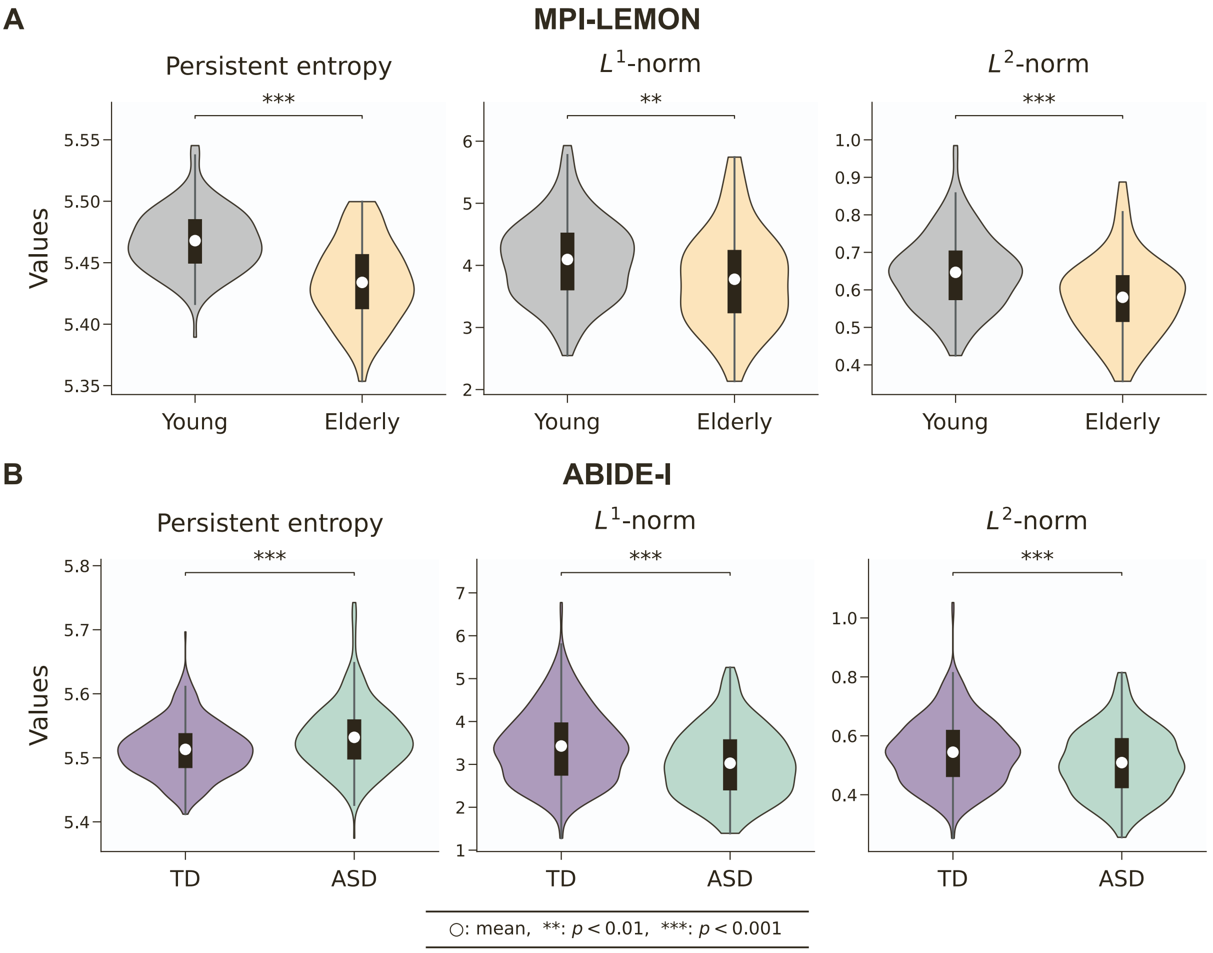

Figure S1: **Brain-wide differences between the groups as identified by three global measures: persistent entropy of the persistence barcodes, L^1-norm, and L^2-norm of the persistent landscape, considering all the correlations from functional connectivity matrices.** (A) MPI-LEMON dataset: violin plots corresponding to 153 young and 72 elderly individuals across three global measures. The mean values of all three measures are significantly higher ($p < 0.01$) in the young group compared to the elderly group. (B) ABIDE-I dataset: violin plots corresponding to 425 typically developing (TD) individuals and 395 individuals with autism spectrum disorder (ASD) across three global measures. Average persistent entropy is significantly higher ($p < 0.001$) in the ASD group than the TD group; however, average L^1-norm and L^2-norm are significantly lower ($p < 0.001$) in the ASD group.

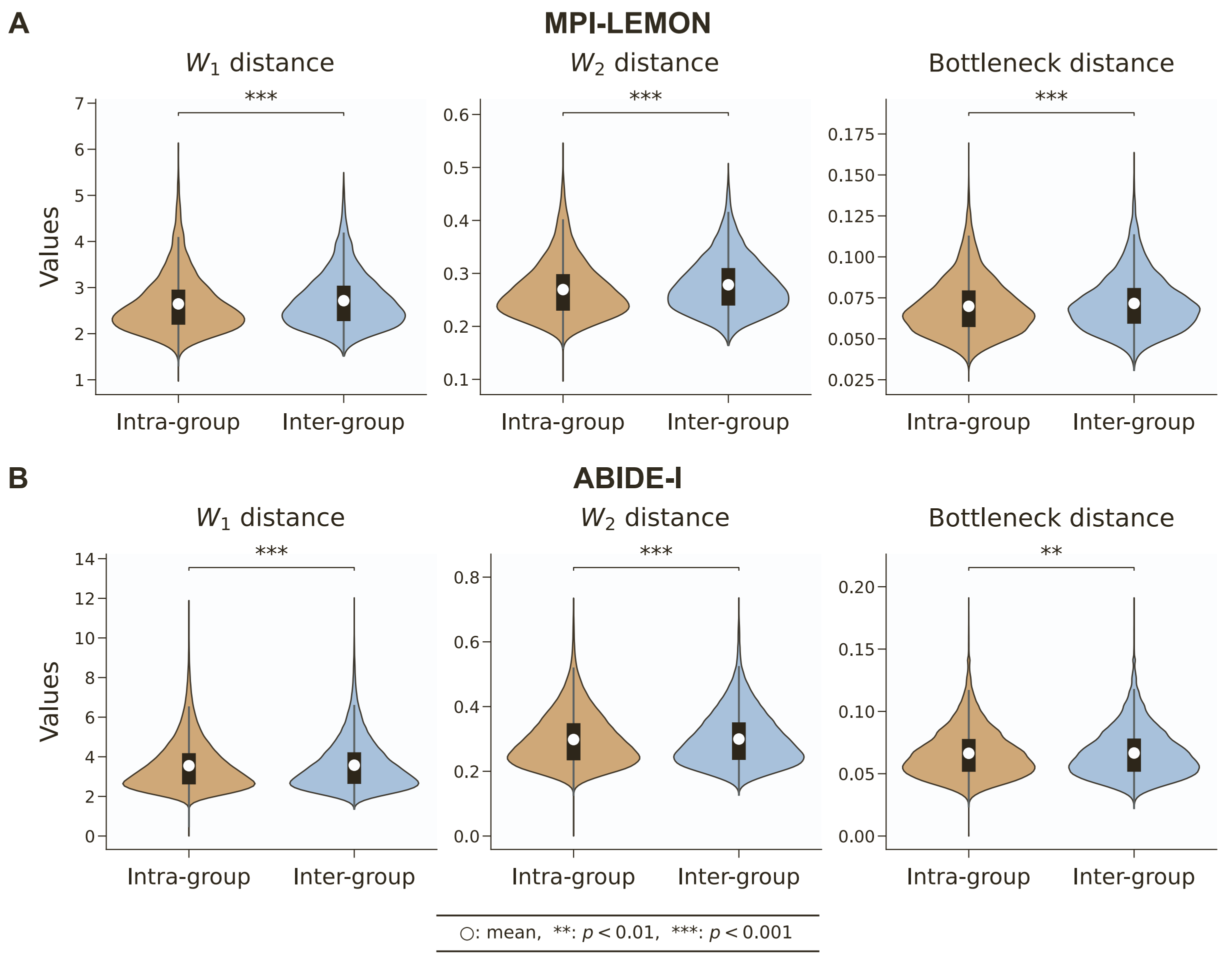

Figure S2: **Brain-wide differences between the intra-group and inter-group distances of persistent diagrams as identified by three global measures: 1-Wasserstein (W_1), 2-Wasserstein (W_2), and bottleneck distances, considering only the positive correlations of the functional connectivity matrices.** (A) MPI-LEMON dataset: violin plots corresponding to intra-group (young-young or elderly-elderly pairs) and inter-group (young-elderly pairs) distances across three measures. The mean values of inter-group distances are significantly higher ($p < 0.001$) than the intra-group distances. (B) ABIDE-I dataset: violin plots corresponding to intra-group (ASD-ASD or TD-TD pairs) and inter-group (ASD-TD pairs) distances across three global measures. The mean values of inter-group distances are significantly higher ($p < 0.01$) than the intra-group distances.

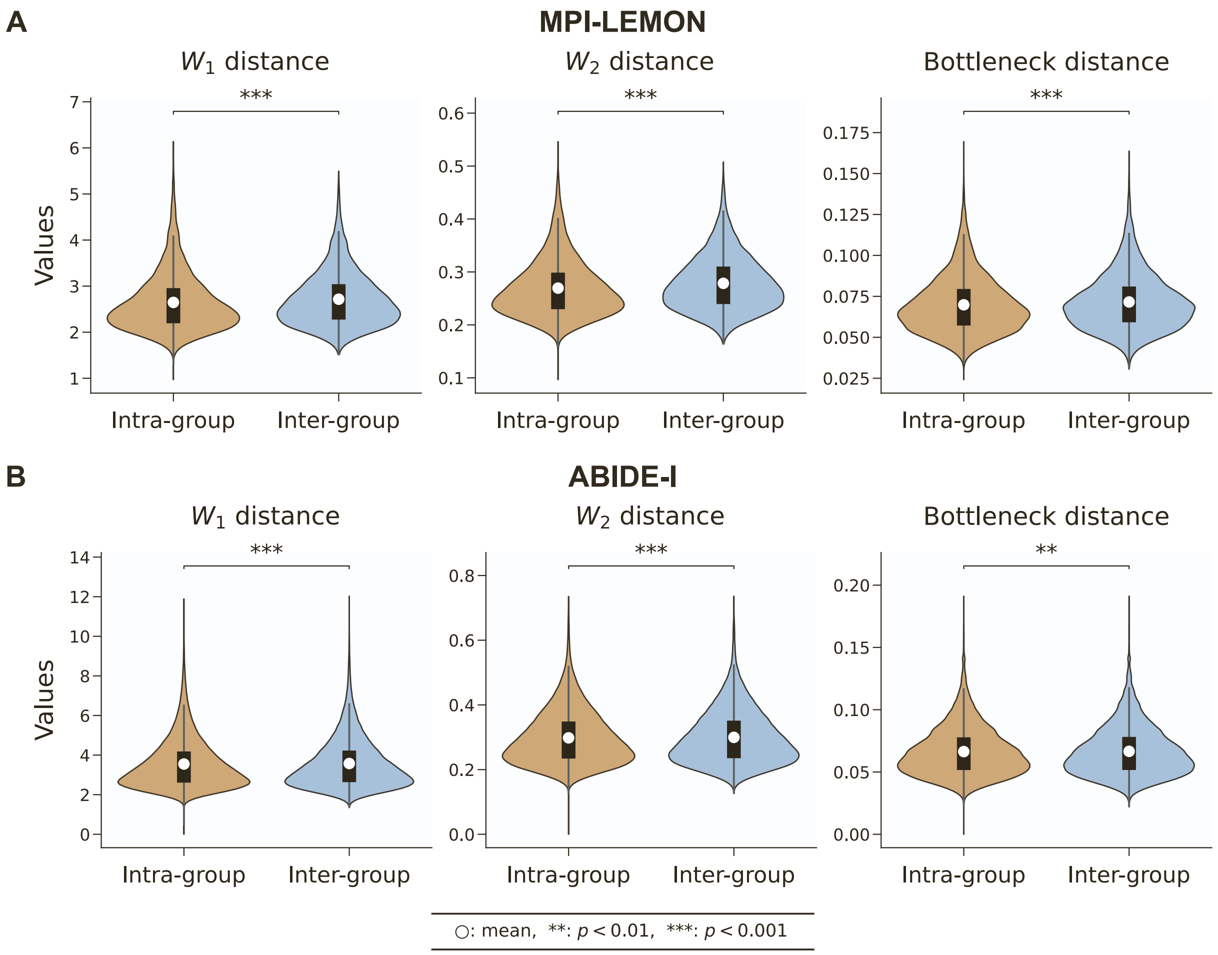

Figure S3: **Brain-wide differences between the intra-group and inter-group distances of persistent diagrams as identified by three global measures: 1-Wasserstein (W_1), 2-Wasserstein (W_2), and bottleneck distances, considering all the correlations of the functional connectivity matrices.** (A) MPI-LEMON dataset: violin plots corresponding to intra-group (young-young or elderly-elderly pairs) and inter-group (young-elderly pairs) distances across three measures. The mean values of inter-group distances are significantly higher ($p < 0.001$) than the intra-group distances. (B) ABIDE-I dataset: violin plots corresponding to intra-group (ASD-ASD or TD-TD pairs) and inter-group (ASD-TD pairs) distances across three global measures. The mean values of inter-group distances are significantly higher ($p < 0.01$) than the intra-group distances.

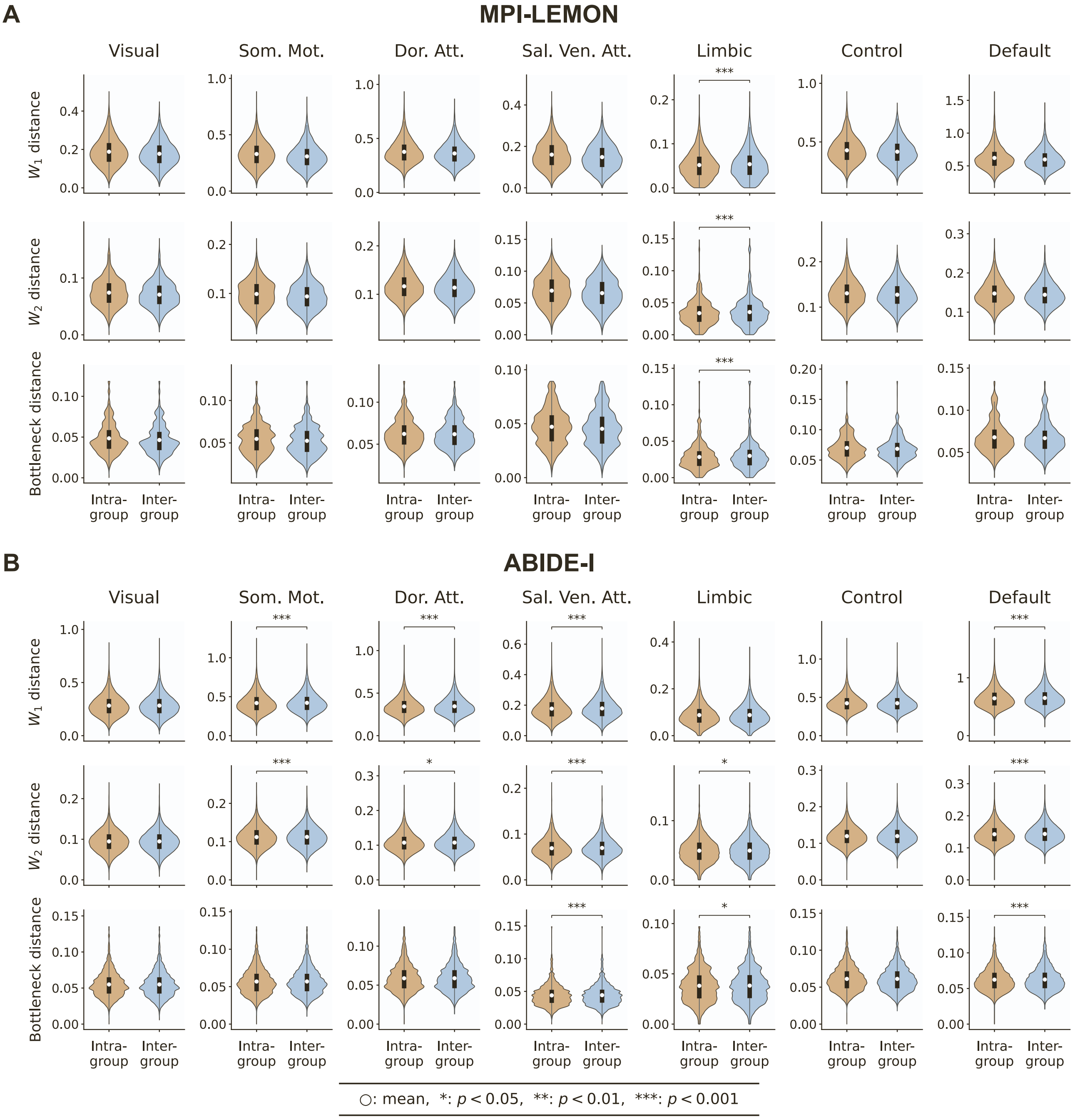

Figure S4: **RSN-level differences between the intra-group and inter-group distances of the persistent diagrams as identified by three global measures: 1-Wasserstein (W_1), 2-Wasserstein (W_2), and bottleneck distances, considering only the positive correlations of the functional connectivity matrices.** Each row corresponds to a given measure, and each column corresponds to a given RSN. (A) MPI-LEMON dataset: violin plots corresponding to intra-group (young-young or elderly-elderly pairs) and inter-group (young-elderly pairs) distances. Inter-group distances are significantly higher ($p < 0.001$) than the intra-group distances only for the limbic network corresponding to all three measures. (B) ABIDE-I dataset: violin plots corresponding to intra-group (ASD-ASD or TD-TD pairs) and inter-group (ASD-TD pairs) distances. For the somatomotor (Som. Mot.) and dorsal attention (Dor. Att.) networks, the inter-group distances are significantly higher ($p < 0.05$) than the intra-group distances for 1-Wasserstein and 2-Wasserstein distances. In the salience/ventral attention (Sal. Ven. Att.) and default networks, inter-group distances are significantly higher ($p < 0.001$) than the intra-group distances for the three measures: 1-Wasserstein, 2-Wasserstein and bottleneck distances. In the limbic network, inter-group distances are significantly higher ($p < 0.05$) than the intra-group distances for 2-Wasserstein and bottleneck distances.

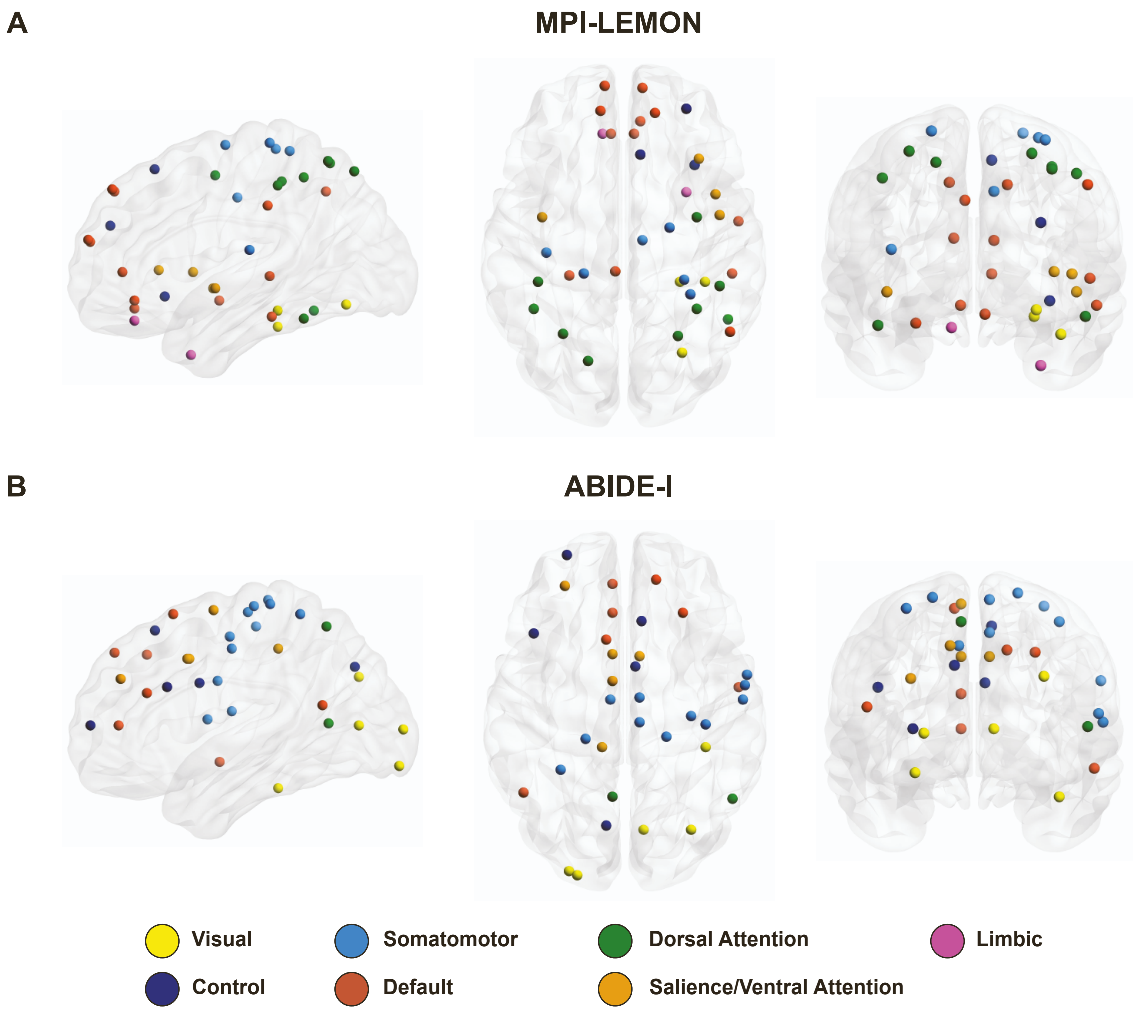

Figure S5: **Visual representation of brain regions with significant between-group differences in node frequency ($p < 0.05$, FDR-corrected).** (A) MPI-LEMON dataset: 39 regions with significant differences in node frequency between the healthy young and healthy elderly groups. For every region, young participants show higher node frequency compared to the elderly participants, except one region RH_Limbic_TempPole_1. (B) ABIDE-I dataset: 35 regions with significant differences in node frequency between the autism spectrum disorder (ASD) and typically developing (TD) groups. All regions reveal increased node frequency for ASD participants relative to TD participants. Each brain region is assigned to one of the seven resting-state networks (RSNs) as defined by the Schaefer atlas. The regions are colored according to their respective RSNs, as detailed in the figure legend. The visualization was generated using BrainNet Viewer. Table S4 lists the significantly different ROIs identified via node frequency across both datasets.

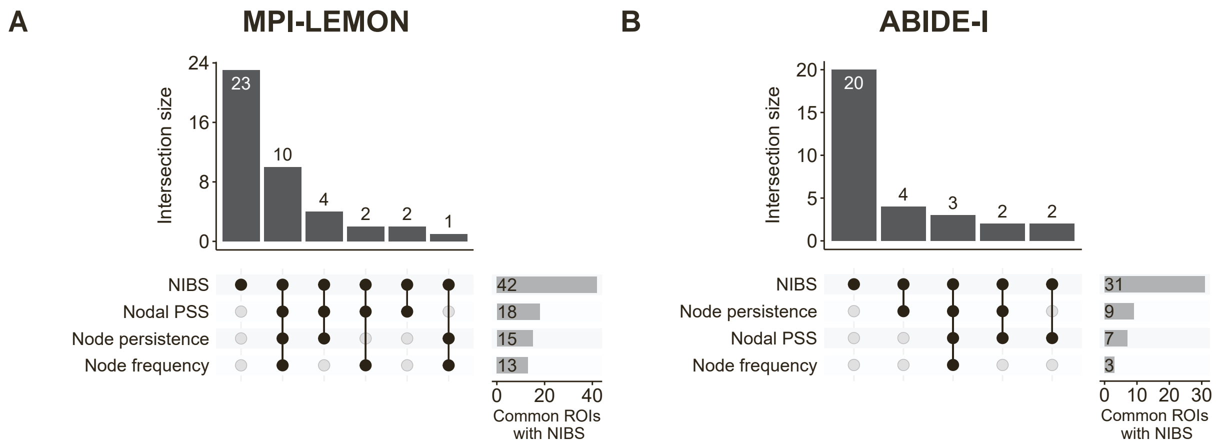

Figure S6: **UpSet plots illustrating the intersection of ROIs identified via non-invasive brain stimulation (NIBS), and local persistent homology-based measures from RSN-to-local level analysis.** Each subplot highlights the combinations of node-based measures, namely node persistence, node frequency, and nodal PSS, with a focus on NIBS-identified regions. The connected cells in the matrix indicate the sets of ROIs involved in each intersection, and the bars aligned with the columns represent the size of each intersection, providing a visual representation of how the sets overlap, particularly emphasizing the role of NIBS in these intersections. The right horizontal bars indicate the cardinality of the intersection of the set of ROIs identified via NIBS and corresponding local persistent homology-based measures. (A) MPI-LEMON dataset: within NIBS, 19 of 42 ROIs are identified considering all three measures collectively, and 10 ROIs are identified when all three are considered simultaneously. Two ROIs are uniquely identified using nodal PSS. (B) ABIDE-I dataset: within the 31 ROIs in NIBS, 11 ROIs are identified considering all three measures collectively, and three ROIs are common across all the measures. Four and two ROIs are uniquely identified considering node persistence and nodal PSS, respectively.

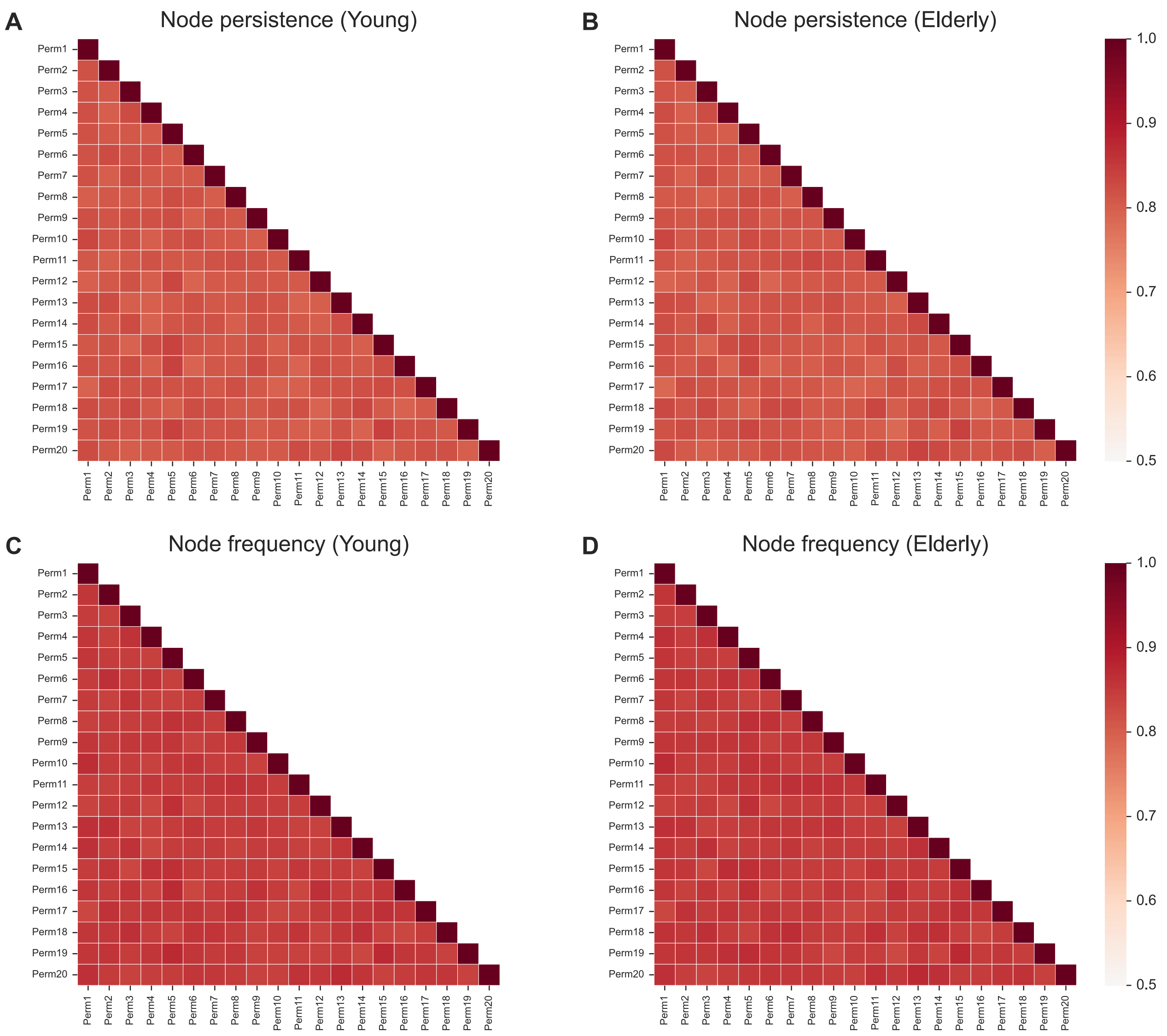

Figure S7: **Pairwise Spearman correlations of the PH-based local measures across 20 random node permutations.** For each permutation, node persistence and node frequency were computed for all nodes in every individual, and subsequently, averaged across individuals within young and elderly groups in the MPI-LEMON dataset. Each subplot shows a 20×20 heatmap corresponding to one measure and one group: (A) node persistence in the young group, (B) node persistence in the elderly group, (C) node frequency in the young group, and (D) node frequency in the elderly group. In all instances, high correlations ($\rho \geq 0.8$) were observed, indicating that node rankings largely remain consistent irrespective of representative cycle choice.

Supplemental notes

Note S1. Correlation of topological measures with phenotypic and clinical scores

We conducted a correlation analysis to examine the relationship of PH-based metrics with phenotypic test scores for the MPI-LEMON dataset and clinical scores of symptom severity for individuals with ASD in the ABIDE-I dataset. This correlation analysis was performed for topological measures on all three scales (see Supplemental methods).

MPI-LEMON dataset provides 52 affective processing-related scores corresponding to all 225 subjects. These scores are divided across eight tests: (a) Cognitive Emotion Regulation Questionnaire (CERQ, 9 scores), (b) Coping Orientations to Problems Experienced (COPE, 14 scores), (c) Emotion Regulation Questionnaire (ERQ, 2 scores), (d) Measure of Affect Regulation Style (MARS, 6 scores), (e) Perceived Stress Questionnaire (PSQ, 5 scores), (f) State-Trait-Angstinventar (STAI-G-X2, 1 phenotypic score), (g) Trait Emotional Intelligence Questionnaire-Short Form (TEIQue-SF, 5 scores), and (h) Trierer Inventar zum Chronischen Stress (TICS, 10 scores).

In the MPI-LEMON dataset, we found that PH-based measures exhibit significant correlations ($p < 0.05$, FDR-corrected) with TICS scores at the global level as well as at the level of RSNs. At the global level, persistent entropy shows significant correlations with 6 out of 10 phenotypic TICS scores, while the L^1-norm and L^2-norm show significant correlations with 1 and 2 scores, respectively. At the RSN-level, these measures show significant correlations with TICS scores within the visual, dorsal attention, and salience/ventral attention networks. Specifically, in the visual network, persistent entropy shows significant correlations with 1 out of 10 TICS scores, whereas both L^1-norm and L^2-norm exhibit significant correlations with 9 out of 10 TICS scores. In the dorsal attention network, persistent entropy and the L^1-norm show significant correlations with 1 and 3 out of 10 TICS scores, respectively. In the salience/ventral attention network, persistent entropy is significantly correlated with 5 TICS scores, while the L^1-norm and L^2-norm exhibit significant correlations with 1 and 2 TICS scores, respectively. Table S7 lists all the correlations between 52 affective processing-related scores and three global measures at both the global and RSN levels.

At the ROI-level, age-related differences detected via node persistence in the salience/ventral attention network are related to affective and somatosensory processing (see Figure 5A). Next, we examined the ROI-level correlations by considering the ROIs with significant between-group differences identified via node persistence. In the salience/ventral attention network, 11 such regions were present, which leads to $11 \times 10 = 110$ correlations. Of these, 21 exhibit significant correlations ($p < 0.05$) before FDR correction. These 21 significant correlations are distributed among seven brain regions. After FDR correction, two ROIs LH_SalVentAttn_FrOperIns_1 and RH_SalVentAttn_FrOperIns_3 show significant correlation with node persistence. All the significant correlations are positive, indicating that higher values of PH-based measures are associated with higher levels of chronic stress. Table S8 provides the ROIs that exhibit significant correlations between all the test scores and node persistence.

In the ABIDE-I dataset, we identified two clinical scores based on the Autism Diagnostic Interview-Revised (ADI-R) criteria that assess symptom severity in individuals with ASD: (a) verbal and (b) social scores. Our analysis revealed no significant correlations between topology-based measures and clinical scores at the global or RSN-level. Correlations between the two clinical scores and the three PH-based measures at both global and RSN-level are provided in Table S9. We further explored ROI-level correlations by focusing on ROIs that showed significant between-group differences based on node persistence. No significant correlations were observed between clinical scores and node persistence at the ROI-level (see Table S8).

Note S2. RSN-to-local level analysis

We computed node persistence and node frequency using Rips complexes constructed from submatrices of the FC Matrix at the level of individual RSNs. A two-tailed two-sample t-test was utilized to detect statistical differences between the groups, and FDR correction was applied independently for each RSN.

In the MPI-LEMON dataset, 59 ROIs exhibit significant differences ($p < 0.05$, FDR-corrected) between the young and elderly groups considering node persistence. These ROIs are distributed among six RSNs: visual (1), somatomotor (24), dorsal attention (5), salience/ventral attention (1), control (1), and default (27) networks. All of these ROIs show higher node persistence values in young individuals compared to elderly individuals. In the ABIDE-I dataset, 53 ROIs exhibit significant differences ($p < 0.05$, FDR-corrected) between the ASD and TD groups via node persistence. These ROIs are distributed among the three RSNs: somatomotor (10), salience/ventral attention (5), and default (38) networks. All the ROIs show higher node persistence values for individuals with ASD compared to TD individuals.

Node frequency reveals 40 and 35 ROIs with significant between-group differences ($p < 0.05$, FDR-corrected) for the MPI-LEMON and ABIDE-I datasets, respectively. In the MPI-LEMON dataset, 40 ROIs are distributed in the RSNs as follows: somatomotor (15), dorsal attention (4), salience/ventral attention (3), control (2), and default (16) networks. Moreover, 37 out of these 40 ROIs identified by node frequency are also identified via node persistence. In the ABIDE-I dataset, 35 ROIs are distributed in three RSNs as follows: somatomotor (8), salience/ventral attention

(8), and default (19) networks. However, 31 out of these 35 ROIs overlap with those found via node persistence. All the ROIs show higher node frequency values in young individuals than in elderly individuals within the MPI-LEMON dataset, and in ASD individuals compared to TD individuals within the ABIDE-I dataset.

In the MPI-LEMON dataset, nodal PSS detects 58 ROIs with significant between-group differences ($p < 0.05$, FDR-corrected) between young and elderly individuals. These include ROIs from six RSNs: visual (2), somatomotor (26), dorsal attention (5), salience/ventral attention (7), control (2), and default (16) networks. Of these 58 ROIs, 47 were also identified via node persistence, and 36 were identified via node frequency. However, in this case, 37 out of 58 ROIs show higher nodal PSS values in young individuals than in elderly individuals. In the ABIDE-I dataset, nodal PSS detects 45 ROIs from the three RSNs, somatomotor (7), salience/ventral attention (14), and default (24), with significant differences between the ASD and TD group ($p < 0.05$, FDR-corrected). Of these 45 ROIs, 35 are also identified through node persistence, and 31 ROIs are identified through node frequency. In this case, most of the ROIs (42 out of 45) display higher nodal PSS values in the ASD group compared to the TD group.

All ROI-level differences identified using node persistence, node frequency, and nodal PSS for both datasets are listed in Table S11. Additionally, Table S12 provides the group-wise averages of node persistence, node frequency, and nodal PSS for each of the 200 ROIs across both datasets, along with the corresponding FDR-corrected p-values.

Note S3. Linking RSN-to-local level analysis with non-invasive brain stimulation outcomes

We investigated the relevance of ROI-level differences in PH-based local measures with the existing literature on non-invasive brain stimulation (NIBS) in healthy elderly individuals and individuals with ASD. As mentioned in the Results section in the main text, target regions that have been shown to enhance motor performance in healthy elderly individuals[S1] correspond to 42 ROIs in the Schaefer atlas. Furthermore, 31 Schaefer ROIs show evidence for improving behavioral or cognitive symptoms associated with ASD[S2].

The UpSet plots in Figure S6 illustrate the number of ROIs associated with clinical improvement and their overlaps with ROIs detected by node persistence, node frequency, and nodal PSS, for both the MPI-LEMON and ABIDE-I datasets. For the MPI-LEMON dataset, out of the 42 clinically relevant ROIs identified by NIBS, 19 are captured by at least one of the topology-based local measures, and 10 are identified by all three measures. In particular, node persistence reveals 15 clinically relevant ROIs spanning three RSNs, node frequency identifies 13 ROIs spanning five RSNs, and nodal PSS reveals 18 ROIs spanning five RSNs (see Figure S6A). For the ABIDE-I dataset, out of the 31 clinically relevant ROIs identified by NIBS, 11 are captured by at least one of the three topology-based local measures, and those are distributed in three RSNs: somatomotor, salience/ventral attention, and default networks. Among these 11 ROIs, 4 ROIs are uniquely identified by node persistence, whereas 2 are uniquely identified by nodal PSS. Specifically, node persistence detects 9 clinically relevant ROIs, node frequency identifies 3, and nodal PSS identifies 7 ROIs (see Figure S6B and Table S10).

Note S4. Robustness of the PH-based local measures

We investigated the robustness of the PH-based local measures by considering different representative cycles of one-dimensional holes. To obtain alternative representations of one-dimensional holes, we applied 20 different permutations of node orderings and rearranged the FC matrices accordingly. For each subject, node persistence and node frequency were then recalculated based on the permuted matrices. We calculated two robust variability indices, median absolute deviation (MAD) and its normalization with respect to the median (MAD/M), to quantify the variability of node-level measures arising from different choices of representative cycles. These indices were calculated separately for each subject and each node. Subsequently, for each group, we computed the average, first quartile (Q1), median (Q2), and third quartile (Q3) of MAD and MAD/M across all subjects.

For node persistence, in the young group, a large proportion of nodes exhibit low variability (see Table S13). Specifically, 140, 198, 174, and 92 nodes (out of 200) have average, Q1, Q2, and Q3 of MAD values below 0.1, respectively, whereas 197, 200, 199, and 170 nodes are below 0.2. In the elderly group, the corresponding numbers are slightly higher, with 173, 200, 189, and 122 nodes falling under the 0.1 threshold. For MAD/M, in the young group, 154, 200, 194, and 88 nodes; and in the elderly group, 164, 200, 193, and 103 nodes have average, Q1, Q2, and Q3 values below 0.2, respectively. For node frequency, the variability assessed using MAD remained low in both groups (see Table S13). In the young group, 183, 200, 198, and 177 nodes have average, Q1, Q2, and Q3 values below 1, respectively. Similarly, in the elderly group, 189, 200, 199, and 181 nodes are below this threshold. Since node frequency can only take integer values, these findings suggest that, across different representative cycle choices, most nodes deviated by at most one unit. These highlight the stability of this measure for most of the nodes. For MAD/M, in the young group, 177, 200, 198, and 123 nodes have average, Q1, Q2, and Q3 values below 0.2, respectively. while in the elderly group, 179, 200, 198, and 128 nodes have values less than 0.2. Therefore, MAD/M values are also consistently low, with the majority of nodes falling below 0.2. Since lower MAD and MAD/M values

reflect higher stability, these results indicate that node persistence and node frequency are largely robust across different representative cycle choices for the majority of nodes. However, a few nodes exhibited MAD/M values exceeding 0.2, suggesting that their node-level scores are more sensitive to the choice of representative cycles.

At the RSN-to-local level, robustness analyses were performed for the three local measures: node persistence, node frequency, and nodal PSS. Here as well, we applied 20 different permutations of node orderings separately for each RSN and rearranged the FC matrices accordingly to obtain different representative cycles. Table S15 provides the average, median (Q2), and third quartile (Q3) of MAD and MAD/M across all subjects, separately for each group and each measure, for all the nodes.

For node persistence, the majority of nodes exhibited no variability at the median level (see Table S15), with 197 and 162 nodes in the young group, and 200 and 176 nodes in the elderly group, showing Q2 and Q3 of MAD values of zero, respectively. A similar trend was observed for MAD/M, where 196 and 148 nodes in the young group, and 198 and 167 nodes in the elderly group, have Q2 and Q3 values equal to zero. Considering average values, 188 nodes in the young group and 194 nodes in the elderly group have MAD values below 0.05, while 154 and 163 nodes in the respective groups have average MAD/M values below 0.2. Similarly, for node frequency, most nodes showed no variability at the median level, with Q2 MAD values of zero in 198 nodes (young) and 200 nodes (elderly), and Q3 MAD values of zero in 171 and 178 nodes, respectively. A similar pattern was observed for MAD/M, where 197 (young) and 199 (elderly) nodes have Q2 values of zero, and 154 and 168 nodes have Q3 values of zero. On average, 187 (young) and 189 (elderly) nodes have MAD values less than 0.3, while nearly all nodes (199 young, 197 elderly) have MAD/M values below 0.2. Similar patterns were observed for nodal PSS. In the young group, 196 and 168 nodes have Q2 and Q3 MAD values equal to zero, respectively, and 191 nodes showed an average MAD below 20. In the elderly group, 199 and 178 nodes have Q2 and Q3 MAD values equal to zero, with 198 nodes showing an average below 20. For MAD/M, 195 and 152 nodes in the young group, and 198 and 168 nodes in the elderly group, have Q2 and Q3 values of zero, respectively. Almost all nodes exhibited low variability, with 197 (young) and 195 (elderly) nodes showing average MAD/M values below 0.2. In summary, the RSN-to-local level analysis shows that node persistence, node frequency, and nodal PSS remain stable under different representative cycle selections for almost all nodes.

Note S5. Robustness of the group-level findings

We analyzed the results for all of the 20 node permutations and identified significantly different regions between the young and elderly groups in the MPI-LEMON dataset for each case. For each permutation, a two-tailed two-sample t-test was used to evaluate group-level differences, and FDR correction was applied to adjust for multiple comparisons.

In our analysis, we considered the node order from the Schaefer atlas and reported the corresponding ROI-level results. Using node persistence, we identified 108 regions with significant between-group differences, of which 27 overlapped with NIBS-identified regions (see Table S10). Across 20 random node permutations, the number of significantly different regions ranged from 99 to 116, with an average of 107. Within these, the number of NIBS-identified regions varied between 21 and 31, averaging 25. To assess consistency, we compared the 108 regions identified from the Schaefer node order with those obtained from each of the 20 random permutations by calculating their overlap. The number of overlapping regions ranged from 87 to 94, with an average of 91. For NIBS-identified regions, the overlap ranged from 19 to 25, with an average of 22. Considering node frequency, 39 significant regions were identified, including 10 overlapping with NIBS. Across 20 permutations, the number of significant regions varied between 38 and 55 (average = 45), with NIBS-identified regions ranging from 7 to 12 (average = 9). The overlap with the reported 39 regions ranged from 27 to 34 (average = 32), and for NIBS regions, the range is from 7 to 10 (average = 8).

We further extended this analysis from RSN-to-local level as well, for the three local measures: node persistence, node frequency, and nodal PSS. For each random node permutation, we computed all three measures across the seven RSNs and obtained the total number of significantly different regions by summing over RSNs for that permutation. Considering node persistence, we identified 59 regions with significant group differences, 15 of which coincided with NIBS-identified regions (see Table S10). Across different permutations, the number of significant regions ranged between 49 and 61 (average = 57), while the subset overlapping with NIBS varied between 13 and 18 (average = 15). When comparing the 59 regions identified from the Schaefer node order with those obtained under different permutations, the overlap ranged from 48 to 56 (average = 53). For NIBS-identified regions, consistency was similarly high, with overlaps of 13 to 15 (average = 14). For node frequency, 40 regions showed significant differences, with 13 overlapping NIBS regions. The number of significant regions fluctuated between 32 and 44 (average = 39) across different permutations, with the number of NIBS-identified regions ranging from 7 to 13 (average = 10). Here as well, we compared the 40 regions identified using the Schaefer atlas node ordering with those obtained from each random permutation by calculating their overlap. The overlaps ranged from 31 to 37, with an average of 33. For NIBS-identified regions, the overlap ranged from 7 to 11, with an average of 9. Employing nodalPSS, 58 regions demonstrated significant between-group differences, with 18 overlapping with NIBS-identified regions. Within 20

random permutations, the number of significant regions ranged from 42 to 59, averaging 52. Here, NIBS-identified regions varied between 13 and 20, with an average of 16. Compared to the node ordering of the Schaefer atlas's 58 significant regions, overlaps ranged between 39 and 48 (average = 44), while NIBS overlaps ranged from 12 to 15 (average = 13). Table S14 summarizes the number of significantly different regions identified using node persistence, node frequency, and nodal PSS across the 20 permutations of node ordering, along with their overlap with NIBS-identified regions and with the regions obtained using the Schaefer atlas node order.

Supplemental methods

Simplicial complex

A simplicial complex is a higher-dimensional generalization of a graph, constructed by combining vertices, edges, triangles, tetrahedra, and their higher-dimensional counterparts. Mathematically, a simplicial complex is defined as a collection K of non-empty subsets of a finite set V that satisfies the following two conditions:

1. If $\sigma \in K$ and $\tau \subsetneq \sigma$, then τ must also be an element of K.
2. For each $v \in V$, the singleton $\{v\}$ is included in K.

The elements of V are called the *vertices* of K, while the elements of K are referred to as *simplices*. If $\tau \subsetneq \sigma$ for simplices $\tau, \sigma \in K$, then τ is termed a *face* of σ and denoted as $\tau < \sigma$. A simplex containing $p+1$ elements is known as a p-*simplex*, meaning it has dimension p. The set of all p-simplices in K is denoted by K_p. The *dimension* of the simplicial complex K is defined as the highest dimension among its simplices. To explore simplicial complexes in greater detail, we recommend standard texts[S3,S4] in algebraic topology.

Vietoris-Rips complex

Let (X, d) be a metric space and S a finite set of points in the metric space, often referred to as a point cloud. For a given radius ϵ (> 0) the Vietoris–Rips complex or Rips complex $R_\epsilon(S)$ is a simplicial complex made up of vertices from S, with simplices formed from finite subsets of S, where each pair of points in a given simplex has a distance at most 2ϵ. Formally, Rips complex $R_\epsilon(S)$ is defined as,

$$R_\epsilon(S) = \{\sigma \subseteq S \mid d(p, q) \leq 2\epsilon, \ \forall\, p, q \in \sigma\}$$

where $d(p, q)$ denotes the distance between points p and q. A 1-simplex (or edge) between two points is added if the distance between them is $\leq 2\epsilon$. Given three points, a 2-simplex (or filled triangle) is formed if the pairwise distances between them are all less than or equal to 2ϵ. In the case of higher-dimensional simplices, a k-simplex arises from a subset of $(k + 1)$ points in S if every pair of points in the subset is connected by an edge.

Rips complex is an efficient method that enables the construction of a simplicial complex on an arbitrary space. Besides Rips complex, there are other methods available, such as Čech complex and alpha complex to construct a simplicial complex[S5]. However, Rips complex is more computationally efficient than Čech complex and is often regarded as an approximation of the latter[S6–S8]. The efficiency arises from the simplicity of its construction, as determining whether a simplex $\sigma \subseteq S$ belongs to $R_\epsilon(S)$ requires only the computation of pairwise distances. For more details regarding Rips complex see e.g.[S9,S10].

Filtration of simplicial complexes

As mentioned, a simplicial complex K is a set of a finite number of simplices, and the simplices are either disjoint or intersect in a common face. A subcomplex is a subset $L \subseteq K$ of the set of simplices such that all the elements in L also satisfy the properties of a simplicial complex.

Given a simplicial complex K, a filtration of length n is a nested sequence of subcomplexes, where each subcomplex is contained within the next. Formally, it is defined as:

$$K_0 \subseteq K_1 \subseteq K_2 \subseteq \cdots \subseteq K_n = K.$$

Here, K_i represents a simplicial complex at the ith stage.

The Rips complex defined in the Methods section admits a natural filtration of simplicial complexes where each subcomplex is indexed by a real parameter ϵ (radius), corresponding to half the distance between pairs of points. Given that only a finite number of point pairs exist, there will be finitely many ϵ values at which new simplices get introduced. At the beginning of the filtration process, i.e., when ϵ is very small, the Rips complex contains only the set of points K_0 in a given metric space. Next, the radius ϵ around each point is gradually increased, and at each

step of the filtration, a simplicial complex is formed. As the radius ϵ increases, higher-dimensional simplices such as edges, triangles, and tetrahedra form. For large ϵ, all points become interconnected, forming a single large simplex. In summary, a filtration tracks how topological features (such as connected components, loops, and voids) evolve across different scales.

Homology

The construction of homology groups of a simplicial complex K begins with a p-chain. A p-chain C_p for an oriented simplicial complex K is defined by the formal linear combinations of oriented p-simplices α_i of K, expressed as:

$$C_p = \sum_{i=1}^{N} c_i \alpha_i$$

where the coefficients c_i are elements from a base field F. Under pointwise addition, the family of p-chains forms a group over the base field F, called the p-dimensional chain group of K, and this group is denoted by $C_p(K)$.

Next, the boundary operator $\partial_p : C_p \to C_{p-1}$ maps the p-chain to the sum of the $(p-1)$-dimensional faces of that p-simplex resulting in a $(p-1)$-chain. Let $\alpha = [x_0, x_1, \ldots, x_p]$ be an oriented p-simplex, and let $[x_0, \ldots, \hat{x}_i, \ldots, x_p]$ denote the $(p-1)$-simplex after the removal of the point x_i from that p-simplex. Then the boundary operator ∂_p is defined as follows:

$$\partial_p(\alpha) = \sum_{i=0}^{p} (-1)^i [x_0, \ldots, \hat{x}_i, \ldots, x_p]$$

The boundary operator satisfies the fundamental property $\partial_p \circ \partial_{p+1} = 0$, which ensures that the boundary of a boundary vanishes. Now, sequentially arranging the chain groups and boundary operators yields a chain complex:

$$\cdots \xrightarrow{\partial_{p+2}} C_{p+1} \xrightarrow{\partial_{p+1}} C_p \xrightarrow{\partial_p} C_{p-1} \xrightarrow{\partial_{p-1}} \cdots$$

Next, p-cycles and p-boundaries are defined from the boundary operator ∂_p. The kernel of the boundary operator ∂_p is called a p-cycle and is denoted by Z_p, i.e.

$$Z_p = \ker(\partial_p) = \{c \in C_p \mid \partial_p(c) = 0\}.$$

In other words, a p-cycle is a p-chain with an empty boundary. A p-boundary is a p-cycle that lies in the image of the boundary operator ∂_{p+1}. The p-boundaries form a group B_p, which is a subgroup of the p-cycles Z_p[S3], i.e.

$$B_p = \mathrm{img}(\partial_{p+1}) = \{c \in C_p \mid \exists\, b \in C_{p+1},\, \partial_{p+1}(b) = c\}.$$

Thus, the simplicial p-homology group of K is defined by the quotient group,

$$H_p(K) = \frac{Z_p(K)}{B_p(K)}.$$

Since the coefficient field is commutative, B_p is a normal subgroup, and H_p is also a group.

The elements of the p-homology group H_p are informally known as p-holes. Notably, H_p forms a vector space over the field F and the dimension of H_p defines β_p, the p-Betti number. Intuitively, this Betti number provides the number of holes formed by p-simplices. For instance, β_0 represents the number of connected components, β_1 represents the number of loops or one-dimensional holes, β_2 represents the number of voids or cavities or two-dimensional holes, and this pattern continues for higher dimensions.

Persistent homology

In the filtration of a simplicial complex K, each subcomplex K_i contains p-chains, p-cycles, p-boundaries, and a p-boundary operator acting on the p-chains. Therefore, the j-persistent p-homology of K_i is defined as,

$$H_p^{i,j} = \frac{Z_p^i}{B_p^{i+j} \cap Z_p^i}$$

where Z_p^i denotes the p-cycles in the subcomplex K_i, B_p^{i+j} denotes the p-boundaries in K_{i+j}. Therefore, the j-persistent p-Betti number of the subcomplex K_i becomes $\beta_p^{i,j} = \dim(H_p^{i,j})$.

The PH of a filtration of simplicial complexes gives more refined information than just the homology of the individual subcomplexes[S11–S13]. In particular, the PH group $H_p^{i,j}$ contains the p-cycles that form in the subcomplex K_i and become boundaries at the subcomplex K_{i+j}. This indicates that the p-cycle persists from step i to step $i+j$ as it appears (or is born) at K_i and disappears (or dies) at K_{i+j}. In simple words, persistence captures the span of the important topological features from their appearance (birth) to their disappearance (death) across the filtration process. For comprehensive details on PH, refer to[S5,S12–S16].

Persistence diagram and persistence landscape

Like barcodes, a persistence diagram also provides a visual representation of the span of the features. A persistence diagram represents a multiset of points $\{(b_i, d_i)\}$ in $\mathbb{R}^2$, representing the birth and death pairs associated with p-holes[S10]. In this diagram, each point (b_i, d_i) is plotted in the Cartesian plane, where the x and y axes represent filtration values at birth and death, respectively. Since $d_i > b_i$, all points lie above the $b = d$ line, and the farther above, the longer the span of the associated p-hole. Persistence diagrams are robust to noisy data[S17]. Distance functions, such as the Wasserstein distance, can be considered to define a metric space of persistence diagrams[S18,S19].

Next, to perform statistical analysis, a persistence diagram is transformed into a sequence of real-valued functions $\Lambda_i : \mathbb{R} \to \mathbb{R}$ for $i \geq 1$, known as the persistence landscape[S20], aggregating the essential information contained in persistence diagrams. The following method is used to construct a persistence landscape from the persistence diagram. First, a clockwise rotation of $45°$ is applied to convert the diagonal of a persistence diagram into the x-axis. Then, isosceles right triangles are drawn from each point (b_i, d_i) of the persistence diagram, considering the point as the vertex. Next, a tent function is constructed from each of the generated triangles. For instance, the second tent function represents the second-highest value at each point across all triangles. In mathematical terms, a piecewise linear function $\Lambda_i : \mathbb{R} \to [0, \infty)$ exists for every birth–death pair $\{(b_i, d_i)\}$ in the persistence diagram defined as:

$$\Lambda_i(t) = \begin{cases} t - b_i & \text{for } t \in [b_i, \frac{b_i+d_i}{2}] \\ d_i - t & \text{for } t \in [\frac{b_i+d_i}{2}, d_i] \\ 0 & \text{otherwise.} \end{cases}$$

The kth largest value among the set $\{\Lambda_i\}$ defines the persistence landscape $\lambda_k(t)$. In this study, we consider $k = 1$, as $\lambda_1(t)$ typically captures the most prominent topological features for each t. As a subset of a Banach space, the persistence landscape enables the calculation of L^p-norms $(1 \leq p \leq \infty)$, which is not the case for persistence diagrams. The L^p-norms of a persistence landscape $\lambda_1(t)$ are defined as[S21]:

$$\|\lambda_1\|_p = \left(\int_{-\infty}^{\infty} |\lambda_1(t)|^p \, dt \right)^{1/p}.$$

In the case of a single birth-death pair (b, d) in the persistence diagram, the norms of $\lambda_1(t)$ are given by: $\|\lambda_1\|_1 = \frac{1}{4}(d - b)^2$ and $\|\lambda_1\|_2 = \frac{1}{2\sqrt{3}}(d - b)^{3/2}$. For multiple non-overlapping birth-death pairs $\{(b_i, d_i)\}$, the norms are computed as: $\|\lambda_1\|_1 = \sum_i \frac{1}{4}(d_i - b_i)^2$ and $\|\lambda_1\|_2 = \sum_i \frac{1}{2\sqrt{3}}(d_i - b_i)^{3/2}$.

Moreover, we calculate the Wasserstein and bottleneck distances to measure the difference between two persistence diagrams[S10]. Let X and Y denote two persistence diagrams. The p-Wasserstein distance is the minimum total cost required to align points (birth-death pairs) from one diagram with another and is mathematically expressed as:

$$W_p(X, Y) = \left(\inf_{\gamma} \sum_{x \in X} \|x - \gamma(x)\|_{\infty}^p \right)^{1/p},$$

where γ represents a bijection between the points of X and Y, allowing points to be matched either to each other or, when necessary, to the diagonal, due to the difference in the cardinalities of X and Y. This distance quantifies disparities in topological features between two datasets. The bottleneck distance is a special case of the Wasserstein distance with parameter $p \to \infty$, defined as:

$$W_{\infty}(X, Y) = \inf_{\gamma} \sup_{x \in X} \|x - \gamma(x)\|_{\infty}.$$

Correlation of topological measures with cognitive, behavioral, and clinical scores

A correlation analysis was conducted to quantify the strength of the linear association between PH-based measures and (i) performance on cognitive/behavioral tests in the MPI-LEMON dataset, and (ii) clinical scores indicating symptom severity in the ABIDE-I dataset. We examined these correlations across all three spatial scales: (a) global scale (brain-wide changes), (b) mesoscopic scale (RSN-level changes), and (c) local scale (ROI-level changes).

The MPI-LEMON dataset includes comprehensive data on 6 cognitive tests and 21 questionnaires addressing emotional tendencies, personality traits, dietary habits, and addiction, accessible at `https://fcon_1000.projects.nitrc.org/indi/retro/MPI_LEMON.html`. We retrieved the phenotypic test scores corresponding to all 225 individuals. Subsequently, we calculated Spearman correlation of metrics derived from PH: persistent entropy, L^1-norm, L^2-norm, and node persistence, with the phenotypic scores available for each cognitive or behavioral test. We employed Spearman correlation as certain test scores are ordinal rather than continuous. Finally, we adjusted the

p-values associated with the estimated correlations using False Discovery Rate (FDR) correction. The FDR correction was implemented separately for p-values of each cognitive or behavioral test. For example, if a given cognitive or behavioral test contains more than one phenotypic score, we performed the FDR correction across all associated scores.

For the ABIDE-I dataset, the analysis was conducted solely for the ASD group. We opted for two clinical scores according to the Autism Diagnostic Interview-Revised (ADI-R) scoring criteria[S22], namely ADI-R verbal and ADI-R social. Among all the potential clinical scores, the ADI-R scores are particularly noteworthy for two main reasons. First, they are available for the largest segment of the ASD group (275 participants). Second, the ADI-R social and verbal scores provide an effective measure of symptom severity in autism[S23]. We calculated the Spearman correlation between PH-based measures and clinical scores. However, no FDR correction was applied at the global and mesoscopic scales since only two scores were considered.

Supplemental references

S1. Yadav, Y., Elumalai, P., Williams, N., Jost, J., and Samal, A. (2023). Discrete Ricci curvatures capture age-related changes in human brain functional connectivity networks. Frontiers in Aging Neuroscience *15*, 1120846. doi:10.3389/fnagi.2023.1120846.

S2. Elumalai, P., Yadav, Y., Williams, N., Saucan, E., Jost, J., and Samal, A. (2022). Graph Ricci curvatures reveal atypical functional connectivity in autism spectrum disorder. Scientific Reports *12*, 8295. doi:10.1038/s41598-022-12171-y.

S3. Munkres, J. R. Elements of Algebraic Topology. CRC press (2018).

S4. Hatcher, A. Algebraic Topology. Cambridge University Press (2002).

S5. Otter, N., Porter, M. A., Tillmann, U., Grindrod, P., and Harrington, H. A. (2017). A roadmap for the computation of persistent homology. EPJ Data Science *6*, 17. doi:10.1140/epjds/s13688-017-0109-5.

S6. Ghrist, R. (2008). Barcodes: The persistent topology of data. Bulletin of the American Mathematical Society *45*, 61–75. doi:10.1090/S0273-0979-07-01191-3.

S7. Dantchev, S., and Ivrissimtzis, I. (2012). Efficient construction of the Čech complex. Computers & Graphics *36*, 708–713. doi:10.1016/j.cag.2012.02.016.

S8. Kerber, M., and Sharathkumar, R. (2013). Approximate Čech Complex in Low and High Dimensions. In: Algorithms and Computation. Springer Berlin Heidelberg (666–676). doi:10.1007/978-3-642-45030-3_62.

S9. Vietoris, L. (1927). Über den höheren Zusammenhang kompakter Räume und eine Klasse von zusammenhangstreuen Abbildungen. Mathematische Annalen *97*, 454–472. doi:10.1007/BF01447877.

S10. Edelsbrunner, H., and Harer, J. Computational Topology: An Introduction. American Mathematical Society (2010).

S11. Robins, V. (1999). Towards computing homology from finite approximations. In: Topology Proceedings vol. 24. (503–532).

S12. Edelsbrunner, H., Letscher, D., and Zomorodian, A. (2002). Topological Persistence and Simplification. Discrete & Computational Geometry *28*, 511–533. doi:10.1007/s00454-002-2885-2.

S13. Zomorodian, A., and Carlsson, G. (2005). Computing Persistent Homology. Discrete & Computational Geometry *33*, 249–274. doi:10.1007/s00454-004-1146-y.

S14. Edelsbrunner, H., and Harer, J. (2008). Persistent Homology – a Survey. Contemporary Mathematics *453*, 257–282.

S15. Carlsson, G. (2009). Topology and data. Bulletin of the American Mathematical Society *46*, 255–308. doi:10.1090/S0273-0979-09-01249-X.

S16. Pun, C. S., Lee, S. X., and Xia, K. (2022). Persistent-homology-based machine learning: a survey and a comparative study. Artificial Intelligence Review *55*, 5169–5213. doi:10.1007/s10462-022-10146-z.

S17. Cohen-Steiner, D., Edelsbrunner, H., and Harer, J. (2005). Stability of persistence diagrams. In: Proceedings of the Twenty-First Annual Symposium on Computational Geometry. (263–271). doi:10.1145/1064092.1064133.

S18. Mileyko, Y., Mukherjee, S., and Harer, J. (2011). Probability measures on the space of persistence diagrams. Inverse Problems *27*, 124007. doi:10.1088/0266-5611/27/12/124007.

S19. Turner, K., Mileyko, Y., Mukherjee, S., and Harer, J. (2014). Fréchet Means for Distributions of Persistence Diagrams. Discrete & Computational Geometry *52*, 44–70. doi:10.1007/s00454-014-9604-7.

S20. Bubenik, P. (2015). Statistical topological data analysis using persistence landscapes. Journal of Machine Learning Research *16*, 77–102.

S21. Bubenik, P. (2020). The Persistence Landscape and Some of Its Properties. In: Topological Data Analysis. (97–117). doi:10.1007/978-3-030-43408-3_4.

S22. Lord, C., Rutter, M., and Le Couteur, A. (1994). Autism Diagnostic Interview-Revised: A revised version of a diagnostic interview for caregivers of individuals with possible pervasive developmental disorders. Journal of Autism and Developmental Disorders *24*, 659–685. doi:10.1007/BF02172145.

S23. Lefort-Besnard, J., Vogeley, K., Schilbach, L., Varoquaux, G., Thirion, B., Dumas, G., and Bzdok, D. (2020). Patterns of autism symptoms: hidden structure in the ADOS and ADI-R instruments. Translational Psychiatry *10*, 257. doi:10.1038/s41398-020-00946-8.
